# Insights on Single-Dose Espresso Coffee Capsules’ Volatile Profile: From Ground Powder Volatiles to Prediction of Espresso Brew Aroma Properties

**DOI:** 10.3390/foods10102508

**Published:** 2021-10-19

**Authors:** Guido R. Lopes, Sílvia Petronilho, Andreia S. Ferreira, Mariana Pinto, Claúdia P. Passos, Elisabete Coelho, Carla Rodrigues, Cláudia Figueira, Sílvia M. Rocha, Manuel A. Coimbra

**Affiliations:** 1 LAQV-REQUIMTE, Chemistry Department, Campus Universitário de Santiago, University of Aveiro, 3810-193 Aveiro, Portugal; guido@ua.pt (G.R.L.); a39493@ua.pt (A.S.F.); a39045@ua.pt (M.P.); cpassos@ua.pt (C.P.P.); ecoelho@ua.pt (E.C.); smrocha@ua.pt (S.M.R.); mac@ua.pt (M.A.C.); 2 Chemistry Research Centre-Vila Real, Department of Chemistry, University of Trás os-Montes and Alto Douro, Quinta de Prados, 5001-801 Vila Real, Portugal; 3 Delta Ventures, Av. Infante D. Henrique 151-A, 1950-405 Lisboa, Portugal; carla.rodrigues@grupo-nabeiro.pt; 4 Diverge, Grupo Nabeiro Innovation Center, Alameda dos Oceanos 65, 1.1, 1990-208 Lisboa, Portugal; claudia.figueira@gruponabeiro.com

**Keywords:** espresso coffee, single-dose, capsules, coffee powder, coffee brew, aroma profile, multivariate analysis

## Abstract

Single-dose coffee capsules have revolutionized the coffee market, fueling espresso coffee popularity and offering access to a wide selection of coffee blends. Nevertheless, scarce information related to coffee powder and brew’s combined volatile characterization is available. In this study, it is hypothesized that coffee brew aroma characteristics can be predicted based on coffee powder’s volatile composition. For this, headspace solid-phase microextraction (HS-SPME) combined with comprehensive two-dimensional gas chromatography with time-of-flight mass spectrometry detection (GC × GC-ToFMS) was used. The data were combined via chemometric tools to characterize in depth the volatile composition of eight blends of capsule-coffee powder and respective espresso brews, simulating the consumer’s perception. A total of 390 volatile compounds were putatively identified, 100 reported for the first time in roasted coffee or brews. Although the same chemical families were determined among the coffee powders and espresso brews, a different volatile profile was determined for each matrix. The Pearson correlation of coffee powders and respective brews allowed to identify 15 volatile compounds, mainly terpenic and esters recognized by their pleasant notes, with a strong relationship between the amounts present in both matrices. These compounds can be key markers to predict the volatile aroma potential of an espresso brew when analyzing the coffee powder.

## 1. Introduction

Coffee is a lifestyle product that has entered the daily routine of many people worldwide, meaning moments of rest and relaxation are associated with the social interaction around a coffee brew. The popularity of coffee relies also on its beneficial effects, such as the stimulating action attributed to caffeine, antioxidant properties of chlorogenic acids, and also on the unique and complex aroma, a decisive factor for coffee brews’ acceptability [1]. The aroma complexity is directly linked to the coffee’s volatile composition, which is dependent, among other things, on the species and varieties of the beans, their geographic origins, roasting conditions, and on the extraction method used to prepare the coffee brew [2,3,4].

Different gas chromatography (GC)-based approaches, mainly using mass spectrometers (MS) as detectors, have contributed to highlight the high complexity of the aroma of different coffee matrices, thus allowing identification of more than 800 volatile compounds belonging to different chemical families [5,6]; namely, pyrazines, pyridines, pyrroles, furans, volatile phenols, oxazoles, thiophenes, thiazoles, thiols and other sulfur compounds, ketones, alcohols, aldehydes, esters, lactones, alkanes/alkenes, and carboxylic acids, directly in roasted coffee beans [7,8,9] or in ground coffee [10,11,12], as well as in different kinds of coffee brews, which include espresso coffee [12,13,14,15,16,17]. Most of these aroma constituents arise during the roasting of the coffee that potentiate the occurrence of caramelization, and/or Maillard reactions derived from green bean non-volatile compounds [18]. The aroma constituents may also result from the wet fermentation process. Moreover, coffee bean varietal compounds, such as the terpenic ones, can survive the roasting process, being also present in roasted coffee beans and brews, contributing to the final complex coffee aroma [19,20,21].

Coffee brews may be obtained using different extraction methods, among which the single-dose capsules system is a device adopted worldwide [22] to prepare espresso coffee, whose use has grown hugely over the years [23]. Its popularity relies on (i) the ready-to-use capsules that simplify the preparation, avoiding the need for grinding or measuring the amount of powder to prepare the brew, bringing convenience and diminishing human variation error, (ii) the reproducibility of extraction conditions [24], and (iii) the diversity of capsule blends that offers a plurality of options to consumers regarding flavors and aromas of the brews. Thus, the consumer may perceive, in a few seconds, the flavor of the brew and the creamy foam layer of an espresso coffee brew [25]. These attributes are directly linked to the characteristics of the ground coffee powder (and consequently of the coffee beans and roasting) used to prepare a single-dose espresso brew, which is usually made of mixtures of coffee powders from different origins and roasting intensities (coffee blends). Despite single-dose capsules’ popularity, to the best of our knowledge, few studies deal with the volatile profile analysis of capsule-blend espresso coffee brews [25,26,27] and capsule-coffee powder [28], but none deal with the simultaneous analysis of both capsule-coffee powder and its respective brew. However, the different composition of single-dose coffee capsule blends should be reflected in volatile profiles that may differ when considering the analysis of capsule-coffee powder or the final espresso coffee brew. 

To overcome this food matrix complexity, a highly sensitive and high throughput technique is needed for the detection of the volatile compounds present in the matrix (i.e., capsule-coffee powder and its respective espresso coffee brew), including the trace ones. Thus, this work aims to perform an in-depth characterization of the capsule-coffee powder of eight commercial single-dose blends and respective brews using the combination of headspace solid-phase microextraction (HS-SPME) with comprehensive two-dimensional gas chromatography with time-of-flight mass spectrometry detection (GC × GC-ToFMS). The approach used should allow definition of a volatile profile for each blend to be used as a tool for prediction of coffee brew aroma characteristics based on coffee powder’s volatile composition.

## 2. Materials and Methods 

### 2.1. Coffee Capsules under Study and Espresso Coffee Preparation

Delta Q^®^ commercial single-dose coffee capsules were kindly provided by NovaDelta-Comércio e Indústria de Cafés, S.A. (Campo Maior, Portalegre, Portugal). In this study, different commercial blends were analyzed (www.mydeltaq.com/, accessed on 1 September 2020), namely a decaffeinated coffee blend (Blend Dec), blends with different labeled intensities (Blend 1—intensity 5, Blend 2—intensity 9, Blend 3—intensity 10), selected origin blends (Blend 4—Jamaica, Blend 5—Tanzania, Blend 6—Ethiopia), and a blend supplemented with natural extracts of ginseng and guarana (Blend Sup). The volatile composition of the ground powder of each capsule was analyzed immediately after opening the capsule. Moreover, all espresso coffee brews (40 ± 2 mL) were prepared by extraction with tap water of each capsule containing ca. 6 g of ground roasted coffee, using an espresso coffee machine (Delta Q^®^, QOSMO model, 19 bars, 900 W, Campo Maior, Portalegre, Portugal). The espresso coffee brews were prepared and immediately analyzed.

### 2.2. HS-SPME Experimental Conditions

The SPME holder for manual sampling and fiber coatings was purchased from Supelco (Aldrich, Bellefonte, PA, USA) and the SPME fiber DVB/CAR/PDMS (1 cm stable-flex^TM^ fused silica fiber, coated with partially cross-linked 50/30 μm divinylbenzene/carboxen/polydimethylsiloxane) was conditioned before use, according to the manufacturer’s recommendations. To avoid any cross-over contamination due to own fiber coating, blanks corresponding to the analysis of the fiber coating not submitted to any extraction procedure were run between sets of three analyses. All measurements were made with three replicates, each replicate representing the analysis of one different aliquot of each coffee sample.

- Coffee powders: the analysis of the powdered coffees was performed based on a previous study [29]. Briefly, for each HS-SPME assay, 1.2 g of sample was placed into a 12 mL glass vial that was capped with a PTFE septum and a screw cap (Chromacol, Hertfordshire, UK). The vial was placed in a thermostatic bath adjusted to 55.0 ± 0.1 °C, and the SPME fiber DVB/CAR/PDMS was inserted into the headspace for 12 min.
- Espresso coffee brew: the SPME assay used for the espresso coffee brew was defined to simulate the consumer’s perception when drinking an espresso coffee. So, based on a previously reported work [30], each espresso coffee sample (40 mL ± 2) was extracted directly into a thermostatized SPME glass vial (120 mL, 60.0 ± 0.1 °C, for 5 min), which was sealed and kept at 60 °C, at constant stirring (ca. 400 rpm), and the DVB/CAR/PDMS fiber was immediately inserted into the sample headspace for 3 min. The temperature of 60 °C was chosen since it is about the same temperature as the coffee would normally be when consumed by the consumer [17].

To establish a basis for comparison between the coffee powder and the respective brew, the conditions for the GC × GC−ToFMS analysis were the same for the two types of coffee samples. 

### 2.3. GC × GC-ToFMS Analysis

After the extraction/concentration step, the SPME fiber containing the headspace volatile compounds was manually introduced into the LECO Pegasus 4D (LECO, St. Joseph, MI, USA) GC × GC-ToFMS injection port at 250 °C and kept for 30 s for desorption. An Equity-5 column (30 m × 0.32 mm I.D., 0.25 μm film thickness, Supelco, Inc., Bellefonte, PA, USA) was used as ^1^D column and a DB-FFAP (0.79 m × 0.25 mm I.D., 0.25 μm film thickness, J&W Scientific Inc., Folsom, CA, USA) was used as a ^2^D column. The carrier gas used was helium at a constant flow rate of 2.50 mL/min and the primary oven temperature program was: initial temperature 35 °C (hold 1 min), raised to 170 °C (5 °C min^−1^), and then to 230 °C (20 °C min^−1^) (hold 2 min). The secondary oven temperature program was 20 °C offset above the primary oven. The MS transfer line and the MS source temperatures were 250 °C. The ToFMS was operated at a spectrum storage rate of 100 spectra/s. The mass spectrometer was operated in the EI mode at 70 eV using a range of *m/z* 35–350 and the voltage was −1468 V. The modulator temperature was kept at 20 °C offset (above primary oven). The modulation time was 6 s and total ion chromatograms were processed using the automated data processing software ChromaTOF at signal-to-noise threshold of 200. Contour plots were used to evaluate the separation general quality and for manual peak identification (Figure S1). For identification purposes, the mass spectrum of each detected compound was compared to those in mass spectral libraries, which included an in-house library of standards and two commercial databases (Wiley 275 and US National Institute of Science and Technology (NIST) V. 2.0—Mainlib and Replib). Mass spectral match factor (similarity >800, but >900 in 85% of the compounds) was also used to decide whether a peak was correctly identified or not. The identification was also supported by experimentally determined linear retention index (RI) values that were compared with those reported in the bibliography for chromatographic columns like the one used in the present work as the ^1^D column (Table S1 in Supplementary Material). RI values were determined using a C_8_-C_20_ *n*-alkanes series (the solvent *n*-hexane was used as C_6_ standard) and calculated according to the van den Dool and Kratz equation [31]. The DTIC (deconvoluted total ion current) GC × GC area data were used as an approach to estimate the relative content of each volatile component in single-dose espresso coffee capsules powders and respective brews and were expressed as arbitrary units (a.u.). Each sample was analyzed in triplicate corresponding to three independent extractions.

### 2.4. Data Processing

A heat map representation was used to compare the full dataset (390 volatile compounds for the 8 commercial single-dose coffee capsules powders and respective brews, independently analyzed with 3 independent replicates) thus performing principal component (PCA) and hierarchical cluster (HCA) analyses using MetaboAnalyst 5.0 (web software, The Metabolomics Innovation Centre (TMIC), Edmonton, AB, Canada) [32]. Peak areas of all compounds (390) were extracted from the chromatograms and used to build the data matrix. The data were mean-centered and divided by the standard deviation of each variable (autoscaling), using Ward’s minimum variance method for clustering analysis. A complete list of the putatively identified compounds is provided in Table S1. The representation of the data was performed using GraphPad Prism version 8 for Windows (trial version GraphPad Software, San Diego, CA, USA). Moreover, a statistical relationship between the GC × GC peak areas of coffee powders and brews for each putatively identified compound was evaluated through the Pearson’s correlation coefficient (r) using data from all the samples under study.

## 3. Results and Discussion

### 3.1. Volatile Composition of Single-Dose Espresso Coffee-Based Blends

**Figure 1:**
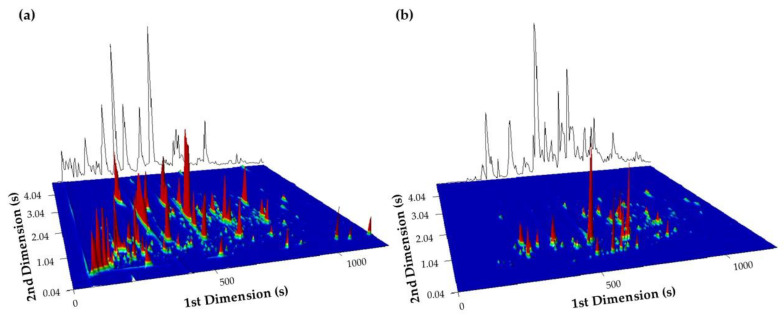
Blowup of a GC × GC-ToFMS surface plot obtained in (**a**) full-scan and (**b**) extracted ion (*m/z* 93, 161, and 204) acquisition modes for espresso coffee brew from Blend 6.

Figure 1a presents a 3D GC × GC-ToFMS chromatogram plot obtained in full scan acquisition mode of an espresso coffee brew from Blend 6 in which several hundred compounds were observed. Figure 1b shows an extracted ion chromatogram of diagnostic ions for varietal compounds (*m/z* 93, 161, and 204), as the terpenic ones, which allowed a rapid identification of a particular type of volatiles. This highlights the importance of using a highly sensitive and high throughput technique to overcome the complexity of coffee samples, detecting both the major compounds as well as the ones present in trace amounts, namely those relevant to distinguishing among coffees with different geographical origins [19].

To determine the volatile profile of single-dose espresso capsule coffee-based blends, a range of eight commercial samples was studied by HS-SPME/GC × GC-ToFMS, differing in the labeled blend intensity (Blends 1 to 3, intensity 1 < 2 < 3) and origins (Blend 4, Jamaica, Blend 5, Tanzania, and Blend 6, Ethiopia), a decaffeinated blend (Blend Dec), and a blend supplemented (Blend Sup) with natural plant extracts of guaraná (*Paullinia cupana*) and ginseng (*Panax ginseng*). A total of 390 volatile compounds (ranging from 381 to 386 in coffee powders and 380 to 387 in espresso brews), distributed over 17 chemical families, including acids (4), alcohols (12), aldehydes (26), esters (40), furan compounds (63), hydrocarbons (25), ketones (60), volatile phenols (5), oxazoles (9), pyrazines (36), pyridines (9), pyrroles (11), sulfur compounds (12), terpenic compounds (29), norisoprenoids (6), thiazoles (19), and thiophene compounds (24), was determined in the assayed single-dose coffee capsule blends (Table 1, chromatograms in Figure S1 and chromatographic details in Table S1). These chemical families were already reported in different coffee samples [5,6,9,21] commonly associated with industrial coffee production during fermentation (acids, alcohols, aldehydes, and esters) and roasting processes (furan compounds, ketones, pyrazines, pyridines, and pyrroles), as well as plant varietal compounds, thus they were already present in the green coffee beans (terpenic compounds and norisoprenoids) [19,20,21].

From the 390 volatile compounds putatively identified, ca. 26% (100 out of 390 volatile compounds) were determined for the first time in roasted coffee and/or in different types of coffee brews. These included 1 alcohol, 4 aldehydes, 18 esters, 7 furans, 10 hydrocarbons, 18 ketones, 1 volatile phenol, 3 oxazoles, 8 pyrazines, 2 pyridines, 1 sulfur compound, 12 terpenic compounds, 5 norisoprenoids, 3 thiazoles, and 7 thiophene compounds. The aroma descriptors of some of these compounds have already been noted, related mainly to fruity, sweet, and floral notes of esters, ketones, and norisoprenoids, and to herbaceous and green notes related to 2,5-diethyltetrahydrofuran and 2-isopropyl-4-methylthiazole (Table 1). This suggests that these compounds, if present in amounts higher than their odor threshold values, can contribute to the overall aroma of the coffee samples under study.

**Figure 2:**
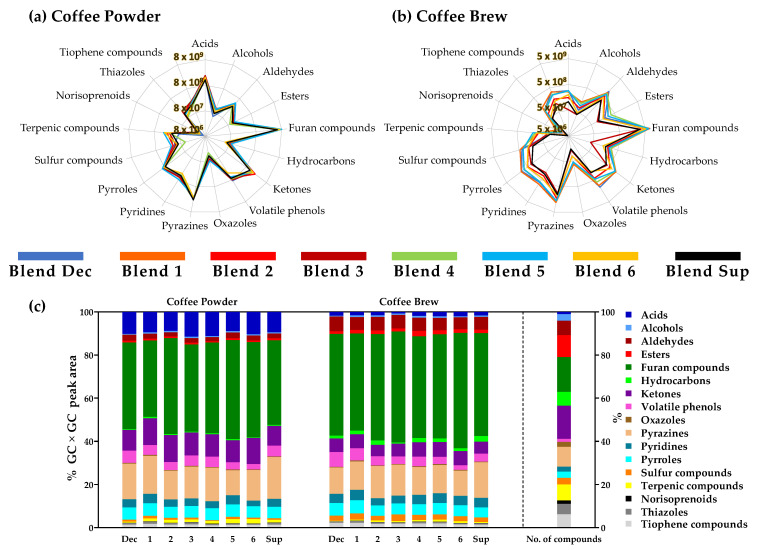
Capsule-coffee blends’ volatile profile analysis. Total GC × GC peak area grouped by chemical family of (**a**) coffee powders and (**b**) espresso coffee brews, and (**c**) contribution of each family to the total area (right column refers to the number of compounds in each chemical family).

To make a global and fast visual comparison among the volatile composition of coffee powders and espresso brews of the eight capsule-coffee blends, radar graphs with the total GC × GC peak area data for each chemical family were constructed (Figure 2a,b) based on data from Table S2. The relative proportion of GC × GC peak area for each family and the relative contribution of the number of compounds in each family is also presented (Figure 2c).

Globally, furan compounds and pyrazines were the major chemical families determined considering the GC × GC peak area on both coffee matrices, corresponding respectively to 35.5–45.9% and 11.5–19.4% in coffee powders and 45.2–53.3% and 11.7–16.5% in espresso brews of the total GC × GC peak area. The predominance of furans and pyrazines over other chemical families was already described for different coffee samples as the main coffee brew aroma contributor [15], related to sweet, fruity, spicy, coffee, nutty, toasted, and roasted notes (Table S1). Moreover, among the 36 pyrazines herein determined, alkylpyrazines such as 2-ethylpyrazine and 2-ethyl-6-methylpyrazine have been previously indicated as potent key odorants in coffee [13,67].

Radar representations in Figure 2a,b show that the volatile compounds profile was different considering GC × GC peak areas among the samples within each matrix, with the differences in the coffee espresso brews being more prominent compared to those of the powders. Indeed, a similar volatile composition was determined among the eight capsule-coffee powders under study, with the chemical families linked to the industrial roasting process having less variation; namely, pyrazines (1.3-fold), furan compounds (1.6-fold), and acids (1.5-fold). Showing a contrasting composition were volatile phenols (2.3-fold), sulfur compounds (3.7-fold), and varietal ones such as terpenic compounds (3.9-fold). Blend Dec (decaffeinated) exhibited a lower content of terpenic compounds, although with the highest areas considering volatile phenols. Regarding volatile phenols, for origin blends the lowest GC × GC peak areas were determined in Blend 6 (from Ethiopia—3.8 × 10^8^) and higher values in Blend 5 (5.8 × 10^8^—Tanzania) and Blend 4 (7.2 × 10^8^—Jamaica). This trend was also observed for these brews (Figure 2, Table S2). Volatile phenols, such as guaiacol, 4-ethylguaiacol, and 4-vinylguaiacol are well-known for their smoky, spicy, and phenolic notes (Table S1), which suggest distinct aroma properties of these origin capsule-coffees. Sulfur compounds, such as 2-furfurylthiol and 2-furylmethylsulfide, contribute with garlic and coffee notes. Thus, the powder of Blend 4 should stand out among all samples due to the lowest GC × GC peak areas of these compounds. The opposite was observed for Blend 5. Moreover, the lower relative content of varietal compounds determined in Blend Dec (terpenic and norisoprenoids compounds), well-known for their fruity, sweet, and floral notes (Table S1), could be related to the decaffeination process, similar to what has been observed for other chemical families in decaffeinated coffees [25,68].

While the variation among powder samples ranged from 1.3 to 3.9-fold (RSD (%)—relative standard deviation expressed as a percentage—of 9–38% among all samples) in the respective brews the variation increased to 2.3–6.8-fold (RSD of 29–63%). Indeed, only the chemical family of sulfur compounds varied less in brews (3.3-fold) than in coffee powders (3.7-fold). However, when analyzing all the 390 compounds, it was verified that the RSDs of the powder compounds were higher than those of the brews in 79% of the cases (Figure S2) due to solid matrix heterogeneity.

Although furan compounds and pyrazines contributed to a similar overall volatile pattern (53.1–58.7% for powders, and 58.4–65.7% for brews), the ketones (9.1–12.5% of overall intensity) and acids (8.9–11.5%) of coffee powders stand out as the representative families, whereas in the brews, aldehydes (5.5–6.9%) and ketones (5.3–5.8%) occupied these positions (Figure 2c). Esters and hydrocarbons, associated with sweet and fruity notes, also had a higher prevalence in the brews when compared to the powders. 

Figure 2b shows that the brew of Blend 3 had the lowest GC × GC peak areas of hydrocarbon and acid families. This blend also exhibited the lowest values for alcohols, esters, pyrazines, and thiazoles (Table S2). In contrast, Blend 1 showed the highest values across all samples for alcohols, hydrocarbons, ketones, oxazoles, pyrazines, pyridines, pyrroles, sulfur compounds, norisoprenoids, thiazoles, and tiophene compounds. Indeed, it was verified that the GC × GC peak areas for all chemical families followed the trend of Blend 1 (intensity 5) > Blend 2 (intensity 9) > Blend 3 (intensity 10), associating a lower labeled intensity of the espresso brews with a higher GC × GC peak area (Figure S3). It was also verified that Blend Sup (supplemented with plant extracts) exhibited the lowest GC × GC peak areas for ketones, oxazoles, pyrroles, sulfur, and terpenic compounds, norisoprenoids, and tiophene compounds. These results suggest that the addition of the extracts decreased the potential aroma of the brew, although it increased other non-volatile components such as carbohydrates and caffeine (the main goal intended by the manufacturer with the plant extracts addition) [23,69].

Considering the 390 compounds putatively identified (Figure 2c), the furans family accounted for the highest number of compounds (63), although with a quite lower relative dominance (16.2%) related to their GC × GC peak areas. Indeed, some families had a considerable number of compounds, although this did not correspond to a high relative contribution in GC × GC peak area, such as terpenic compounds (29 compounds—7.4%, but 0.6–2.0% peak area in powders and 0.2–0.9% in brews), thiophene compounds (24 compounds, <2.2% of peak area) or thiazoles (19 compounds, <1.1% of peak area). In contrast, acids and volatile phenols, although in reduced number (5 and 4 compounds, respectively) had a higher impact considering their GC × GC peak areas (2.1–6.8% in both matrices for volatile phenols and 8.9–11.5% in coffee powder for acids). 

### 3.2. Multivariate Analysis of Coffee Powders and Brews

**Figure 3:**
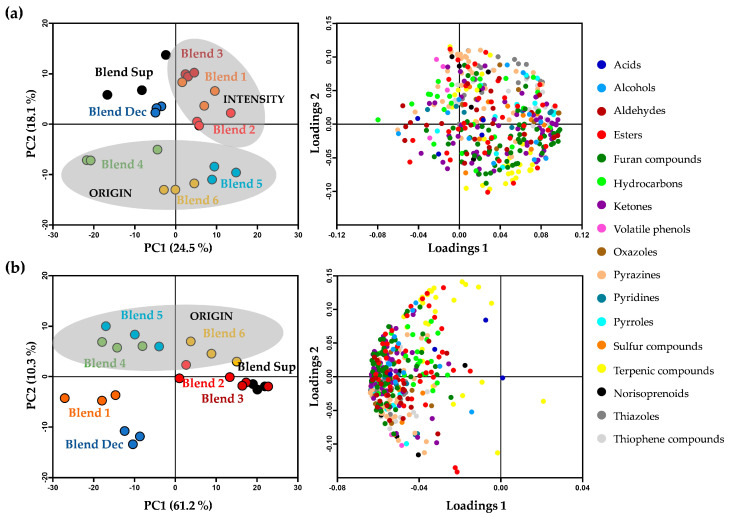
Principal component analysis (PCA) of the 390 volatile compounds, grouped by chemical families, putatively identified in (**a**) capsule-coffee powders and (**b**) espresso coffee brews, presenting the distribution of the samples (scores plot, left) and compounds (loadings plot, right).

To evaluate in depth the variability of the blends according to the respective chromatographic area of the 390 volatile compounds putatively identified, a PCA was built for each coffee sample matrix, with data scaling for attributing equal weight for all compounds (Figure 3, left). The two first PCA components of the brews explained a data variability of 71.5%, which was much higher than the variability explained by these two components in the powders’ dataset (42.6%). This may indicate a higher heterogeneity of the volatile composition of the solid matrix. Independently of the matrix type, origin coffee blends (Blends 4–6) were separated from the others, appearing exclusively on the negative side of PC2 for coffee powders or on the positive side of PC2 for espresso coffee brews. In both cases, this separation was mainly related to the terpenic compounds and some esters (Figure 3, right), recognized by their pleasant notes (Table S1). Moreover, in coffee powders, the varietal terpenic compounds were predominantly distributed in positive loading1 and negative loading2, contributing to the distinction of African Blends 5 and 6 (Tanzania and Ethiopia, respectively) from the American Blend 4 (Jamaica), which was placed on the negative side of PC1 and PC2 (Figure 3a), thus allowing to distinguish these blends according to their geographical origin. However, for the brews (Figure 3b), the Ethiopian Blend 6 was placed in a PC1 positive position, separated from the others (both in PC1 negative) mainly due to the higher abundance of α-humulene (Table S2). The distinctive aroma of Ethiopian brewed coffees in relation to Tanzanian ones is in accordance with literature [21]. 

The coffee powders of Blends 1 to 3 were separated from the others (positive PC1 and PC2), thus revealing a more similar volatile composition among them. According to the loadings plot (Figure 3a, right) this could be explained due to the higher GC × GC peak areas of thiophene compounds, thiazoles, oxazoles, norisoprenoids, and pyrazines (Table S2), thus suggesting possibly different aroma characteristics when compared to the origin, decaffeinated, and supplemented blends.

The coffee powders from Blend Sup and Blend Dec were also separated from the others (PC1 negative and PC2 positive—Figure 3a) due to their lower chromatographic areas in almost all volatile compounds determined (Table S2). This trend was also particularly evident in the espresso coffee brew of Blend Dec (Figure 3b, Table S2). Since Blend Sup had plant extracts, resulting in a smaller amount of coffee powder per gram of sample, its coffee content was lower when compared to those of the other coffee blends, which may explain its lower volatile intensity. Moreover, as the coffee beans of Blend Dec were decaffeinated before roasting and grinding, the conditions used for this process removed many other constituents present in the coffee beans besides caffeine, thus explaining its lower chromatographic areas for almost all the identified chemical families, in agreement with the literature [25,68].

**Figure 4:**
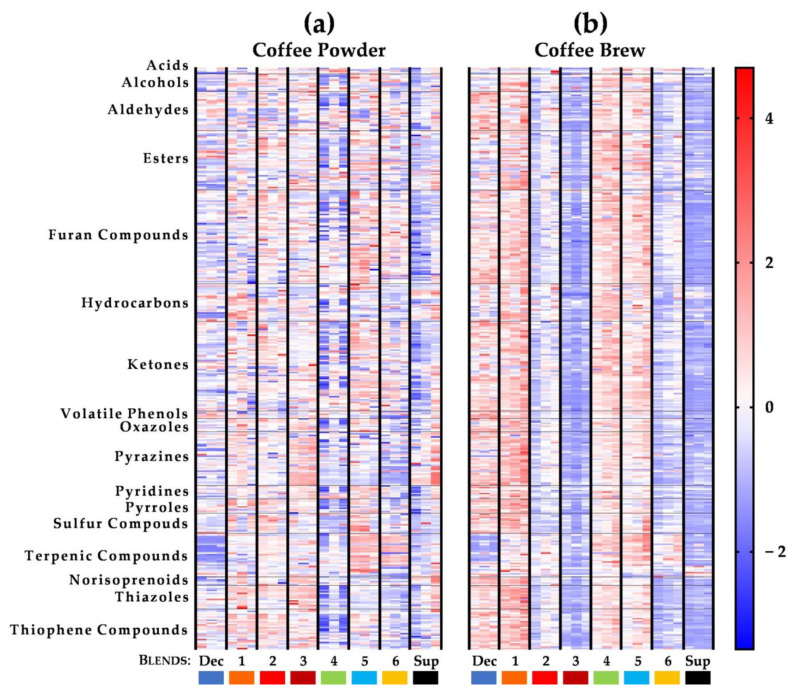
Heatmap representation corresponding to the 390 volatile compounds of the 8 capsule-coffee powders (**a**) and respective espresso brews (**b**) under study, distributed by chemical families, considering the GC × GC peak areas after mean-centering the data for each variable and dividing by the standard deviation (autoscaling). The relative content of each compound is illustrated through a chromatic scale (from dark blue, low values, to dark red, high values). Detail GC × GC peak areas data were reported in Table S2.

To deeply analyze the dataset concerning the volatile pattern of the eight coffee blends (Table S2), a heat map representation of the GC × GC peak areas for each analyzed matrix was performed (Figure 4), highlighting the differences and/or similarities among the volatile components of coffee powders and espresso brews. The representation is based on a chromatic scale (from dark blue, low values, to dark red, high values) obtained after an autoscaling treatment of the data, intending to attribute equal importance/weight for all compounds. Thus, independently of their absolute higher/lower GC × GC peak areas in both matrices, Figure 4 allows a rapid visual assessment of each capsule’s volatile profile and a relative comparison among the eight coffee samples (coffee powders and corresponding espresso brews). Moreover, the heat map shows the effect of all compounds, while the previous analysis (Figure 2) was based on the grouping of compounds by chemical families. 

Contrasting with the observed similarity among the eight coffee powders observed in Figure 2a, the heat map of the powders of Blend 5 (from Tanzania) and Blend 6 (Ethiopia), both Africa origin coffees, exhibited an apparent more intense volatile profile (predominance of red), mainly of furans, esters, ketones, and terpenic compounds, being the major apparent differences related to pyrroles and sulfur compounds that predominate in Blend 5. Esters and terpenic compounds contribute to pleasant aromas [70], such as floral, citrus, and fruity notes (Table S1), which could be translated into coffees from Blends 5 and 6 having more distinct aroma notes. The opposite was observed for the Caribbean blend (Blend 4 from Jamaica), which revealed an apparent less intense volatile profile in the compounds identified when compared to the African ones (Figure 4). Moreover, when looking for the set of coffee powders with increasing intensity, although the heat map highlights their apparent similar volatile profile, it shows an increase in the pyrazines and thiazoles content (predominance of red). This suggests that the labeled intensity, which the manufacturer used to describe its blends, presumably due to different roasting degrees, seems to modulate the pyrazines and thiazoles content, which have aroma descriptors of coffee nutty, green, and toasted notes (Table S1).

The pattern of volatile compounds of the assayed coffee brews (Figure 4b) exhibited an apparent higher color homogeneity across all compounds, highlighting the overall intensity of the blends when comparing to the more heterogeneous patterns of the powders (Figure 4a). Globally, espresso coffee brews from Blend Dec and Blends 1, 4, and 5 were those with the largest chromatographic areas (predominance of red), while Blend 3 and Blend Sup were the ones with the lowest intensity (predominance of blue color). Moreover, as observed for the analysis of the chemical families, the intensity of the volatile compounds apparently diminished gradually with the increase of labeled intensity in a great part of the volatile compounds. Assuming that labeled intensity is associated with roasting degree, it can be inferred that this trend is in accordance with the literature [20]. Furthermore, in contrast to the coffee powders, origin espresso coffee blends revealed a different volatile profile since the intensity of the GC × GC peak areas of most of the compounds diminished from Blends 4 and 5 to Blend 6. The different volatile profiles for the powders and the espresso brews reinforce the importance of considering both powder and coffee brew matrices to make reliable conclusions for each blend. Moreover, terpenic compounds were in relative greater quantity in origin espresso coffees (Blend 4: Jamaica, Blend 5: Tanzania, and Blend 6: Ethiopia) and, notably less in Blend Dec despite the overall high intensity in most of the remaining classes. These varietal compounds can contribute with floral, citrus, and fruity notes in the final aroma of coffee (Table S1), which can be translated into origin coffees with more distinct notes, contrasting to the decaffeinated blend (Blend Dec).

Since the HS-SPME extraction conditions herein used were different for the two matrices, aiming to have the perspective of the consumer when drinking an espresso coffee, and the perspective of the overall volatile composition of coffee powder, it is not possible to make a direct comparison between the GC × GC peak areas. However, it was possible to make a relative comparison among the volatile profiles of each type of capsule-coffee blend (Figure 4): (i) Blend Dec and Blend 1 revealed a similar volatile profile among their coffee powders and respective espresso brews, except for aldehydes, furan and sulfur compounds, ketones, and pyrazines which were less intense in the powder, (ii) Blend 2 and mainly Blends 3 and Sup were less intense in espresso brews, (iii) Blend 4 exhibited a higher volatiles’ intensity in the brews than in the respective powder, and (iv) Blends 5 and 6, only differing in their origins (Tanzania and Ethiopia, respectively) had a more intense volatile profile in the powder when compared to the brew, in particular for the furan compounds, ketones, pyridines, pyrroles, and terpenic compounds. The different matrix structures may explain the distinct extractability of the volatile compounds of the different blends [12,71]. Nevertheless, these results revealed that the variations in espresso coffee brews were not so perceptible when analyzing coffee powders, and the differences determined in coffee powders were not always reflected in the espresso brew. The aqueous nature of the extraction process hinders the passage of coffee powder hydrophobic compounds to the brew. Moreover, the non- or low-water soluble volatile compounds tend to migrate in a higher extent in the brew to the headspace and are perceived by the consumer [63]. As a consequence, only a few compounds relevant to the aroma of the brews are extracted from the solid matrix and perceived in the brew in the same proportion.

### 3.3. Coffee Powder Discriminant Volatiles That Predict Brews’ Profile 

**Figure 5:**
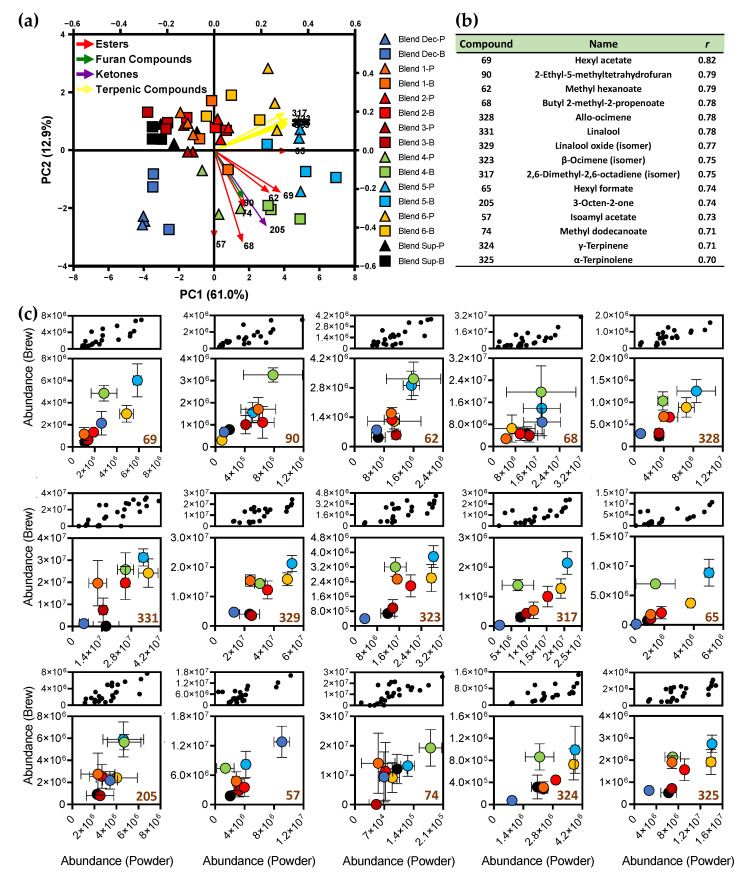
Biplot principal component analysis (PCA) of the 15 volatile compounds (**a**) with Pearson correlation > 0.7 and *p*-value < 0.0001 (Figure S4) (**b**), considering the values of GC × GC peak areas for the 8 commercial coffee powders and the respective coffee brews. The correlations are displayed in graph representations (**c**), with all values represented by black dots (above) and showing the average ± standard deviation of each sample (below). Letters “P” and “B” after the identification of each blend sample correspond to the powder and brew, respectively.

Although the heat map representation of all volatile compounds gave a profile for each blend, both in the powders and brews, it is possible that the analysis of some of these compounds in the coffee powder reflects the volatile potential in the corresponding brew. Thus, considering the Pearson correlation of coffee powders and respective brews (Figure S4), it was found that 15 compounds (hexyl acetate, 2-hexyl-5-methyltetrahydrofuran, methyl hexanoate, butyl 2-methyl-2-propenoate, allo-ocimene, linalool, linalool oxide (isomer), β-ocimene (isomer), 2,6-dimethyl-2,6-octadiene (isomer), hexyl formate, 3-octen-2-one, isoamyl acetate, methyl dodecanoate, γ-terpinene, and α-terpinolene—Figure 5b) exhibited a correlation coefficient (*r*) higher than 0.7, suggesting a strong relationship between these variables. Figure 5 shows a PCA representation of the data considering these 15 compounds (7 terpenic compounds, 6 esters, 1 furan derivative, and 1 ketone (Figure 5a), as well as the coefficients associated (Figure 5b) and the graphical representation of the data for each compound (Figure 5c). The samples grouped together regardless the matrix, powder, and brew (Figure 5a). Thus, it seems that there was a relationship between the amount of these compounds in the powder and in the respective brew. The samples were also grouped in three major clusters: Blend Dec, Sup and intensity blends (1–3), and origin blends (4–6). The distribution in Figure 5a seems to follow the distribution observed in PC2 for all brew compounds (Figure 3b). From the 390 compounds organized in 17 chemical families, terpenic compounds and esters stand out, which are compounds associated with the coffee variety, explaining the association between these compounds and origin blends. Figure 5c highlights that the pattern for all terpenic compounds was similar, contrasting with the pattern for the remaining compounds. On the other hand, intensity blends (as well as Sup) were poorly separated, suggesting they were derived from similar coffee blends, although with different roasting degrees. Thus, these seven terpenic compounds, although usually in low abundance in coffee matrices, can be used to predict the aroma potential of the brew when analyzing the coffee powder.

## 4. Conclusions

The HS-SPME/GC × GC-ToFMS analysis allowed the detailed characterization of single-dose capsule coffee-based blends, relating the volatile composition of coffee powders with the respective espresso brews. This high throughput methodology allowed the detection of a wide number of volatiles (a total of 390 volatile compounds, considering both samples from 8 single-dose commercial coffee capsules), belonging to 17 chemical families. From these, 100 volatile compounds (ca. 26%) were determined for the first time in roasted coffee or coffee brews.

The use of 2D-GC allowed defining a volatile compounds’ profile for each blend, enabling the comparison of blends in respect to the compounds present in trace amounts that could predict coffee brew aroma when analyzing the volatile compounds of the respective coffee powder. The different volatile profile of the powder and the brew from the same capsule blend suggests that both matrices should be considered when defining coffee blend characteristics and potential consumer acceptance. However, the selection of highly correlated compounds between the two matrices can be used to predict the volatile potential of an espresso brew when analyzing the coffee powder.

## Figures and Tables

**Table 1:** Volatile compounds putatively identified in 8 capsule-coffee powders and espresso brews, organized by chemical family, using HS-SPME/GC × GC-ToFMS and aroma descriptors for each compound when available in the literature.

Compound	CAS Number	Reported ^a^	Aroma Descriptor ^b^	References ^c^
**Acids**				
*Aliphatics*				
Acetic acid	64-19-7	[33]	Pungent, sour, acidic, vinegar	[20,34,35,36]
Propanoic acid	79-09-4	[33]	Pungent, rancid, sour milk, cheese, butter-like	[34,36]
Butanoic acid	107-92-6	[33]	Sour, rancid, butter-like, sweaty, rubbish	[34,36,37,38]
Isovaleric acid	503-74-2	[33]	Acidic, cheesy, herbaceous, sweaty, rancid	[34,35,36,37,39]
**Alcohols**				
*Aliphatics*				
2-Methyl-1-propanol	78-83-1	[33]	Wine-like	[36]
3-Buten-1-ol	627-27-0		-	
3-Methyl-3-buten-1-ol	763-32-6	[33]	-	
2-Methyl-1-butanol	137-32-6	[40]	Cooked, roasted with fruity or alcoholic undernotes	[36]
1-Pentanol	71-41-0	[33]	Green, chemical, fusel oil-like sweet	[34,36,41]
3-Methyl-2-buten-1-ol	556-82-1	[33]	Fresh, herbaceous-fruity-green, lavender-like, phenolic, metallic	[34,36]
2-Hexanol	626-93-7	[33]	-	
2-Heptanol	543-49-7	[33]	Fresh, lemon-like, grassy-herbaceous, sweet-floral undertone	[34,36]
1-Octen-3-ol	3391-86-4	[33]	Mushroom, herbaceous, savory, brothy, meaty	[37,41,42]
2-Ethyl-1-hexanol	104-76-7	[13]	Sweet, slightly floral rose-like	[36]
1-Octanol	111-87-5	[43]	Fresh, orange-rose, sweet	[36]
*Aromatics*				
2-Phenylethanol	60-12-8	[33]	Rose-honey-like, floral	[34,36,41]
**Aldehydes**				
*Aliphatics*				
Acetaldehyde	75-07-0	[33]	Pungent, ethereal, fruity, coffee, wine, acrid/egg	[34,36,41,44,45]
2-Methylpropanal	78-84-2	[33]	Pungent, sour, fruity, malty, buttery-oily	[34,36,46]
2-Butenal	4170-30-3	[47,48]	-	
3-Methylbutanal	590-86-3	[33]	Pungent, acrid, fruity, apple-like, almond, malty, sweaty	[34,36,39,44,46]
2-Methyl-2-butenal	1115-11-3	[33]	-	
2-Pentenal	764-39-6		Pungent, green, apple, orange, tomato	[36]
Hexanal	66-25-1	[33]	Fatty, green, grassy, fruity, rancid butter-like, nutty	[34,36,38,41,46]
4-Methyl-3-pentenal	5362-50-5		-	
2-Methyl-2-hexenal	28467-88-1		-	
2,4-Hexadienal	80466-34-8	[33]	Fresh, green, floral, citrus	[36]
4-Methylhexanal	41065-97-8		-	
Heptanal	111-71-7	[33]	Oily-fatty, rancid, pungent, fermented-fruit-like	[34,36]
2-Heptenal	2463-63-0	[43]	Pungent, green, fatty	[36]
Octanal	124-13-0	[33]	Fatty, citrus, orange-like, honey	[2,36]
2,4-Heptadienal	5910-85-0	[49]	Fatty, green	[36]
2-Octenal	2363-89-5	[37]	Green-leafy, orange, honey-like, cognac-like	[36,37]
Nonanal	124-19-6	[19,43]	Fatty, orange and rose note, soap-like, metallic	[36,50]
2-Nonenal	2463-53-8	[33]	Fatty, orris-like, waxy, dried orange peel-like, cardboard-like	[36,38]
Decanal	112-31-2	[19,49,51]	Sweet, waxy, floral, citrus, fatty	[36]
Undecanal	112-44-7	[51]	Sweet, fatty, orange and rose undertone	[36]
Dodecanal	112-54-9	[49,51]	Fatty, violet-like	[36]
*Aromatics*				
Benzaldehyde	100-52-7	[33]	Sweet, bitter almond-like, bitter	[34,36,41]
Benzeneacetaldehyde	122-78-1	[33]	Pungent-green, hyacinth-like, floral, sweet-fruity, honey-like	[34,36,39,50,52]
2-Hydroxybenzaldehyde	90-02-8	[33]	Pungent, herbaceous, spicy-floral, bitter, almond-like	[34,36]
2-Methylbenzaldehyde	529-20-4	[33]	Sweet, beany, fresh pea	[34,37]
2-Phenyl-2-butenal	4411-89-6	[33]	Musty, floral, cocoa	[4]
**Esters**				
*Aliphatics*				
Methyl acetate	79-20-9	[33]	Sweet, ethereal, fruity	[34,36]
Methyl propenoate	96-33-3		-	
Methyl propanoate	554-12-1	[33]	Ethereal-rum-like, sweet, fruity	[34,36]
Methyl glycolate	96-35-5	[47]	-	
Methyl 2-methylpropenoate	80-62-6		Acrid, fruity	[36]
Methyl butanoate	623-42-7	[33]	Sweet-ethereal, fruity, apple peel, peach-like	[34,36]
Methyl 2-butenoate	18707-60-3	[48]	-	
Methyl 2-methylbutanoate	868-57-5		Sweet, fruity, apple-like	[36]
Methyl 3-methylbutanoate	556-24-1	[33]	Ethereal, fruity, apple-like, herbaceous	[34,36]
3-Methylbutyl formate	110-45-2	[37]	Plum, fruity, black currant-like	[36,37]
Butyl acetate	123-86-4	[33]	Pear-like, ethereal, fruity, ripe/over-ripe fruits-like	[34,36]
Methyl pentanoate	624-24-8		Ethereal, green-fruity, apple-like, pineapple- like	[34,36]
Methyl 3-methyl-2-butenoate	924-50-5	[53]	roasted	[54]
Ethyl 3-methylbutanoate	108-64-5	[33]	Fruity, apple-like	[36,38,41]
Isoamyl acetate	123-92-2	[33]	Fruity, banana, sweet, apple-like	[34,36]
Methyl 3-methylpentanoate	2177-78-8		-	
3-Methyl-3-butenyl acetate	5205-07-2		Fruity	[36]
Methyl 4-methylpentanoate	2412-80-8		Sweet, pineapple-like	[36]
Pentyl acetate	628-63-7		-	
Methyl hexanoate	106-70-7	[33]	Pineapple-like, apricot-like, sweet, ethereal	[34,36]
Ethyl tiglate	5837-78-5		Fruity, caramel	[34,36]
3-Methyl-2-butenyl acetate	1191-16-8	[33]	Fresh, fruity, banana-like, bergamot-like	[34]
Hexyl formate	629-33-4		Fruity, apple-like, unripe-plum	[36]
Ethylidene acetate	542-10-9		-	
Methyl 4-Methyl-2-oxopentanoate	3682-43-7		-	
Butyl 2-methyl-2-propenoate	97-88-1		-	
Hexyl acetate	142-92-7	[55]	Fruity, apple, cherry, pear, floral	[36]
Isobutyl 3-methyl-2-butenoate	30434-54-9		-	
Methyl octanoate	111-11-5		Winy, fruity, orange-like	[36]
3-Methylbutyl 3-Methyl-2-butenoate	56922-73-7		-	
Methyl decanoate	110-42-9		-	
Methyl dodecanoate	111-82-0		Fatty, floral, wine-like	[36]
*Aromatics*				
Phenyl acetate	122-79-2	[56,57]	Floral, rosy, dark chocolate-like	[4]
Benzyl formate	104-57-4	[33]	Fruity, green, herbaceous, earthy, floral	[34,36]
Methyl benzoate	93-58-3	[33]	Fruity, cananga-like	[36]
Methyl phenylacetate	101-41-7	[33]	Honey, musky, jasmine, floral	[34,36]
2-Phenylethyl formate	104-62-1	[33]	Green, herbaceous, rosy, hyacinth, chrysanthemum, watercress-foliage	[34,36]
Methyl 2-hydroxybenzoate	119-36-8	[33]	Sweet, rooty-fruity, minty, spicy, wintergreen-like	[34,36]
2-Phenylethyl acetate	103-45-7	[58]	Floral, rose, honey-like	[36]
Ethyl 2-hydroxybenzoate	118-61-6	[48]	Wintergreen	[36]
**Furan compounds**				
Furan	110-00-9	[33]	Spicy-smoky, cinnamon-like	[34]
2-Methylfuran	534-22-5	[33]	Ethereal, sickly	[20]
Tetrahydrofuran	109-99-9	[33]	Sweet-gassy, bread-like	[34]
2,5-Dimethylfuran	625-86-5	[33]	coffee	[20]
2,4-Dimethylfuran	3710-43-8	[48]	-	
2-Propylfuran	4229-91-8	[33]	-	
2-Ethyl-5-methylfuran	1703-52-2	[33]	-	
2-Ethyl-5-methyltetrahydrofuran	931-39-5		-	
2-Furancarbonitrile	617-90-3		-	
Dihydro-2-methyl-3-furanone	3188-00-9	[33]	Bread-like, buttery, nutty	[20,36]
2,3,5-Trimethylfuran	10504-04-8	[33]	-	
3-Furaldehyde	498-60-2	[33]	-	
2-Vinyl-5-methylfuran	10504-13-9	[33]	Coffee	[20]
2-(Methoxymethyl)furan	13679-46-4	[33]	Burnt, herbal, potato-like	[2,37]
2,3,4-Trimethylfuran	10599-57-2	[33]	-	
Furfural	98-01-1	[33]	Sweet, bread-like, caramel-like, cinnamon-almond-like, bitter	[20,34]
2-(2-Propenyl)furan	75135-41-0	[33]	-	
5-Methyl-2(3H)-furanone	591-12-8	[37,59]	Sweet, herbaceous, tobacco-like, coffee, earthy, raw potato skin	[20,36,37]
2-Furanmethanol	98-00-0	[33]	Slightly caramel-like, warm, oily, burnt, bitter	[34,36,37,41]
2,5-Diethyltetrahydrofuran	41239-48-9		Sweet, herbaceous, caramel-like	[36]
2-Butylfuran	4466-24-4	[33]	-	
Furfuryl formate	13493-97-5	[33]	Floral	[20]
2-Acetylfuran	1192-62-7	[33]	Balsamic-sweet, tobacco-like, floral, balsamic-cinnamic, spicy, roasty	[2,20,34,36]
γ-Butyrolactone	96-48-0	[33]	Sweet, slightly buttery	[20,34,36]
2,3,4,5-Tetramethylfuran	10599-58-3	[33]	-	
3-(1,1-Dimethylethyl)-2,3-dihydrofuran	34314-82-4		-	
1-(2-Furyl)-2-propanone	6975-60-6	[33]	Sweet, fruity-caramel-like, spicy, radish	[34,36]
2-Methyl-5-propenylfuran	5555-95-3	[33]	Candy, fruity, sweet	[37]
Dihydro- 5-methyl-2(3H)-furanone	108-29-2	[33]	Sweet, hay-like, tobacco-like, herbaceous	[34,36]
5-Methylfurfural	620-02-0	[33]	Sweet, spicy, caramel	[20,34,36,41]
2-Acetyl-5-methylfuran	1193-79-9	[33]	Nutty	[36]
Methyl 2-furoate	611-13-2	[33]	Berry-like, fruity, winey, mushrooms-like, fungus-like, tobacco-like	[34,36]
2-Pentylfuran	3777-69-3	[33]	Fruity, green bean, metallic, vegetable	[36]
Benzofuran	271-89-6	[33]	-	
Furfuryl acetate	623-17-6	[33]	Ethereal-floral, herbal-spicy, fruity, banana, nutty	[20,34,36]
2,5-Dihydro-3,5-dimethyl-2-furanone	5584-69-0	[19,21]	-	
1-(2-Furanyl)-1-propanone	3194-15-8	[33]	-	
2,2’-Bifuran	5905-00-0	[33]	-	
3,4-Dimethyl-2,5-furandione	766-39-2	[33]	-	
1-(5-Methyl-2-furyl)-2-propanone	13678-74-5	[33]	-	
1-(2-Furanyl)-2-butanone	4208-63-3	[33]	-	
5-Ethyl-2-furaldehyde	23074-10-4	[33]	-	
1-(2-Furanyl)-3-butanone	699-17-2	[33]	-	
2,2′-Methylenebisfuran	1197-40-6	[33]	-	
3-Acetyl-2,5-dimethylfuran	10599-70-9		-	
Furfuryl propanoate	623-19-8	[33]	Spicy, floral, fruity	[36,60]
1-(2-Furanyl)-1-butanone	4208-57-5	[33]	-	
2-Methylbenzofuran	4265-25-2	[33]	-	
2-Methyl-5-propionylfuran	10599-69-6	[33]	-	
1-(5-Methyl-2-furanyl)-2-butanone	13678-70-1	[33]	-	
2-Heptylfuran	3777-71-7	[33]	Roasted, nutty	[36]
Furfuryl butanoate	623-21-2	[33]	-	
2-(2-Furanylmethyl)-5-methylfuran	13678-51-8	[33]	-	
*n*-Furfuryl pyrrole	1438-94-4	[33]	Vegetable, green, earthy, horseradish, mushroom-like	[36,60]
2-Methyl-3(2-furyl)acrolein	874-66-8		-	
4-(2-Furanyl)-3-buten-2-one	623-15-4	[33]	Spicy-woody, sweet, cinnamon-like, balsamic, vanilla, woody	[20,36]
4,7-Dimethylbenzofuran	28715-26-6		-	
2-Furyl pyrazine	32736-95-1	[33]	-	
2,2′-Methylenebis(5-methylfuran)	13679-43-1	[33]	-	
1-(5-Methylfurfuryl)pyrrole	13678-52-9	[33]	Mushroom-like, green, pharmaceutical, roasty	[2,60]
Difurfuryl ether	4437-22-3	[33]	-	
1-Furfuryl-2-formylpyrrole	13788-32-4	[33]	green, minty	[20]
2-Acetyl-1-furfurylpyrrole	13678-73-4	[33]	-	
**Hydrocarbons**				
*Aliphatics*				
Nonane	111-84-2	[33]	-	
1,3-Nonadiene	-		-	
Decane	124-18-5	[33]	-	
Undecane	1120-21-4	[33]	-	
1-Dodecene	112-41-4		-	
Dodecane	112-40-3	[33]	-	
Tridecane	629-50-5	[33]	-	
Tetradecane	629-59-4	[33]	-	
Pentadecane	629-62-9	[33]	-	
Hexadecane	544-76-3	[33]	-	
*Aromatics*				
Methylbenzene	108-88-3	[33]	Sweet-gassy	[34]
Ethylbenzene	100-41-4	[33]	Sweet-gassy	[34]
1,3-Dimethylbenzene	108-38-3	[33]	-	
Phenylethylene	100-42-5	[33]	Sweet-gassy, balsamic, floral	[34,36]
1-Methylethylbenzene	98-82-8		-	
1-Ethyl-4-methylbenzene	622-96-8	[33]	-	
1,2,3-Trimethylbenzene	526-73-8		-	
1-Methyl-3-propylbenzene	1074-43-7		-	
Butylbenzene	104-51-8		-	
4-Ethyl-1,2-dimethylbenzene	934-80-5		-	
1-Ethyl-2,3-dimethylbenzene	933-98-2		-	
1,2,4,5-Tetramethylbenzene	95-93-2	[33]	-	
1,2,3,4-Tetramethylbenzene	488-23-3	[61]	-	
Pentylbenzene	538-68-1		-	
1-Butylheptylbenzene	4537-15-9		-	
**Ketones**				
*Aliphatics*				
2-Propanone	67-64-1	[33]	Ethereal, lemon	[34,41]
2-Butanone	78-93-3	[33]	Ethereal, sweet apricot-like	[34,36]
2,3-Butanedione (Diacetyl)	431-03-8	[33]	Buttery	[34,35,36,44,46]
2-Pentanone	107-87-9	[33]	Ethereal-fruity, wine	[34,36]
2,3-Pentanedione	600-14-6	[33]	Buttery, oily, sweet, caramel-like, milky	[2,20,34,35,36,37,44]
1-Hydroxy-2-propanone	116-09-6	[33]	Sweet-caramel-like, mushroom, earthy, nutty	[20,34,37]
4-Methyl-2-pentanone	108-10-1	[33]	Ethereal-fruity, spicy	[34,36]
3-Penten-2-one	625-33-2	[33]	Fruity	[36]
3-Hydroxy-2-butanone (Acetoin)	513-86-0	[33]	Creamy-fatty-buttery, woody, yogurt	[20,34,36,41]
2-Methyl-1-penten-3-one	25044-01-3		-	
2,4-Pentanedione	123-54-6	[33]	Ethereal-minty, metallic	[34]
3-Hexanone	589-38-8	[33]	Ethereal, grape, wine-like	[37]
2,4-Dimethyl-3-pentanone	565-80-0	[33]	-	
2-Hexanone	591-78-6	[33]	-	
3,4-Hexanedione	4437-51-8	[33]	Buttery, toasty, burnt, nutty, caramel-eggy	[34,36]
1-Hydroxy-2-butanone	5077-67-8	[33]	Toasted	[20]
3-Hydroxy-2-pentanone	3142-66-3	[33]	Earthy, aged	[37]
2-Hydroxy-3-pentanone	5704-20-1	[33]	-	
3-Hexen-2-one	763-93-9		-	
3-Hexene-2,5-dione	4436-75-3		-	
4-Methyl-2-hexanone	105-42-0		-	
4-Heptanone	123-19-3	[33]	Ethereal-fruity, pineapple-like, strawberry-like	[34]
1-(Acetyloxy)-2-propanone	592-20-1	[33]	Fruity-buttery, sour	[34]
3-Heptanone	106-35-4	[33]	Green, fatty, fruity, sweet, ethereal	[34,36]
2-Heptanone	110-43-0	[33]	Fruity, spicy, cinnamon, banana, spicy	[36]
3-Hepten-2-one	1119-44-4	[37]	Green-grassy	[36]
2,5-Hexanedione	110-13-4	[33]	Sweet-ethereal	[34]
6-Methyl-3-heptanone	624-42-0		-	
6-Methyl-2-heptanone	928-68-7		-	
5-Methyl-2-heptanone	18217-12-4		-	
1-Octen-3-one	4312-99-6	[33]	Mushroom	[36,38,45,62]
2,3-Octanedione	585-25-1	[33]	Warmed-over	[36]
6-Methyl-5-hepten-2-one	110-93-0	[33]	Strong, fatty, green, citrus-like	[36]
2-Octanone	111-13-7	[33]	Floral, bitter-green, musty-herbaceous, unripe-apple fruity	[34,36]
3-Octen-2-one	1669-44-9	[33]	Fruity, lemon	[36]
2-Nonanone	821-55-6	[33]	Fruity-floral, fatty, herbaceous, rue	[36,60]
3-Nonen-2-one	14309-57-0		Fruity	[36]
*Cyclics*				
2-Cyclopenten-1-one	930-30-3	[33]	-	
Cyclohexanone	108-94-1		Peppermint, acetone-like	[36]
4-Cyclopentene-1,3-dione	930-60-9	[20,63]	-	
2-Methyl-2-cyclopenten-1-one	1120-73-6	[33]	-	
2-Cyclohexen-1-one	930-68-7	[33]	Gassy-mint	[34]
5-Ethylcyclopent-2-en-1-one	34094-63-8		-	
6-Methylenebicyclo[3.2.0]hept-3-en-2-one	-		-	
3-Methyl-2-cyclohexen-1-one	1193-18-6	[33]	Caramel-like, phenolic, mild cherry	[36,60]
2,2,6-Trimethylcyclohexanone	2408-37-9		-	
2-Cyclohexene-1,4-dione	4505-38-8		-	
3,5-Dimethyl-2-cyclohexen-1-one	1123-09-7		-	
2-Hydroxy-3-methyl-2-cyclopenten-1-one	80-71-7	[33]	Sweet, caramel-like-spicy, walnut, maple, licorice, celery, tobacco	[34,35,52]
2,3,4-Trimethyl-2-cyclopenten-1-one	28790-86-5	[29]	-	
3,5-Dimethyl-1,2-cyclopentanedione	13494-07-0	[33]	-	
3-Ethyl-2-hydroxy-2-cyclopenten-1-one	21835-01-8	[33]	Caramel-like, sweet, sugary	[36,60]
*Aromatics*				
Acetophenone	98-86-2	[33]	Sweet	[36]
1-Phenyl-2-propanone	103-79-7		-	
*o*-Hydroxyacetophenone	118-93-4	[33]	Sweet, heavy-floral, herbaceous, new-mown hay-like, mimosa-like	[34]
1-Phenyl-1,2-propanedione	579-07-7	[33]	Warm-floral, herbaceous, plastic	[34,36]
*p*-Methylacetophenone	122-00-9		Fruity, floral	[36]
1-Phenyl-2-butanone	1007-32-5		-	
1-(4-Hydroxyphenyl)-1-propanone	70-70-2		-	
4-Hydroxy-3-methylacetophenone	876-02-8	[64]	-	
**Volatile phenols**				
2-methoxyphenol (Guaiacol)	90-05-1	[33]	Smoke-like, phenolic, burnt, spicy, woody, meaty, sweet	[20,35,36,39,44,60]
2,6-Dimethylphenol	576-26-1	[33]	Ground-coffee, phenolic	[50,60]
2-Allylphenol	1745-81-9		-	
4-Ethyl-2-methoxyphenol (4-Ethylguaiacol)	2785-89-9	[33]	Smoky, clove-like, spicy, burnt, vanilla-like, sweet, ethereal, green	[20,36,39,44,60]
2-Methoxy-4-vinylphenol (4-Vinylguaiacol)	7786-61-0	[33]	Spicy, clove-like, phenolic, apple, rum, roasted peanut	[20,34,36,39,44,60]
**Oxazoles**				
4-Methyloxazole	693-93-6		-	
4,5-Dimethyloxazole	20662-83-3	[33]	-	
Trimethyloxazole	20662-84-4	[33]	-	
4-Ethyl-2,5-dimethyloxazole	30408-61-8	[33]	-	
2-Ethyl-4-methyl-5-propyloxazole	102586-53-8		-	
4,5-Dimethyl-2-propyloxazole	53833-32-2	[33]	-	
4,5-Dimethyl-2-isobutyloxazole	26131-91-9		-	
Benzoxazole	273-53-0	[33]	-	
2-Methylbenzoxazole	95-21-6	[33]	Sweet, gassy-pungent, floral-sweet, tobacco	[34,60]
**Pyrazines**				
Pyrazine	290-37-9	[33]	Pungent, sweet, floral, coffee	[20,34,54]
Methylpyrazine	109-08-0	[33]	Nutty, cocoa, green, roasted, chocolate, meaty, toasted	[20,34,54]
2,5-Dimethylpyrazine	123-32-0	[33]	Chocolate, roasted nuts, earthy, grassy, roasted, nutty	[36,54,60]
2-Ethylpyrazine	13925-00-3	[33]	Peanut butter, musty, nutty, woody, buttery, roasted, green, sweet	[36,60]
2,3-Dimethylpyrazine	5910-89-4	[33]	Nutty, cocoa-like odor, green note, toasted, roasted	[20,36,54]
Vinylpyrazine	4177-16-6	[33]	-	
2-Isopropylpyrazine	9820-90-0		-	
2-Ethyl-6-methylpyrazine	13925-03-6	[33]	Toasted, flowery, fruity, hazelnut-like	[2,20,54]
2-Ethyl-3-methylpyrazine	15707-23-0	[33]	Raw-potato, roasted, earthy, nutty, peanut-like, coffee-like	[20,36,54]
2-Propylpyrazine	18138-03-9	[33]	Green, vegetable, herbal	[2,36]
2-Vinyl-6-methylpyrazine	13925-09-2	[33]	Coffee	[20]
Acetylpyrazine	22047-25-2	[33]	Toasted	[20]
2-Methyl-3-isopropylpyrazine	15986-81-9		-	
Isobutylpyrazine	29460-92-2		-	
Isopropenylpyrazine	38713-41-6	[33]	-	
2,6-Diethylpyrazine	13067-27-1	[33]	Toasted, potato-like, roasted	[2,20,54]
2-Isopropyl-3-methoxypyrazine	25773-40-4	[33]	Vegetable-like, earthy, bell pepper, raw potato, galbanum, roasty, peasy	[36,38,39,60]
6,7-Dihydro-5*H*-cyclopentapyrazine	23747-47-9	[33]	Green, phenolic, nutty, roast	[35,60]
2-Acetyl-3-methylpyrazine	23787-80-6	[33]	Cereal, roasted bean, roasted, nutty, grain-roasted potato	[36,60]
2-Isobutyl-3-methylpyrazine	13925-06-9	[33]	Herbaceous green earthy notes, green bell peppers notes	[36]
5*H*-5-Methyl-6,7-dihydrocyclopentapyrazine	23747-48-0	[33]	Earthy, baked potato, peanut, roasted, nutty	[35,36,60]
2-Methyl-6-(1-propenyl)pyrazine (isomer)	104638-11-1	[33]	-	
2,3-Diethyl-5-methylpyrazine	18138-04-0	[33]	Nutty, meaty, roasted hazelnut, earthy, roasty	[35,36,44,45,52]
2-Methyl-6-(1-propenyl)pyrazine (isomer)	104638-11-1	[33]	-	
2-Acetyl-3-ethylpyrazine	32974-92-8	[29]	-	
2-Isoamylpyrazine	40790-22-5		-	
2-Isobutyl-3-methoxypyrazine	24683-00-9	[33]	Green bell-pepper note, galbanum oil, red pepper, green, earthy	[4,36,44,60]
2-Butyl-3-methylpyrazine	15987-00-5	[33]	-	
2,5-Dimethyl-3-isobutylpyrazine	32736-94-0	[33]	-	
1-Methylpyrrolo(1,2-a)pyrazine	64608-59-9	[33]	-	
6,7-Dihydro-2,5-dimethyl-5*H*-cyclopentapyrazine	38917-61-2	[33]	-	
2,5-Diethyl-3,6-dimethylpyrazine	18903-30-5	[33]	-	
2-Methyl-6-isopentylpyrazine	91010-41-2		-	
2,6-Dimethyl-3(2-methyl-1-butyl)pyrazine	56617-70-0		-	
2,5-Dimethyl-3-isoamylpyrazine	18433-98-2		-	
2,3-Dimethyl-5-isopentylpyrazine	18450-01-6		-	
**Pyridines**				
Pyridine	110-86-1	[33]	Pungent, nauseating, warm, burnt, smoky, coffee-like	[20,34,36,54]
2-Methylpyridine	109-06-8	[33]	Roasted popcorn, coffee	[20,60]
2,6-Dimethylpyridine	108-48-5	[33]	Minty-tarry, pyridine, peppermint	[36]
2-Ethylpyridine	100-71-0	[33]	-	
3-Ethylpyridine	536-78-7	[33]	Tobacco, caramel, burnt, coffee-like, toasted	[20,36,54,60]
3-Vinylpyridine	1121-55-7		-	
2-Acetylpyridine	1122-62-9	[33]	Popcorn, bready, tobacco, cracker-like, roasted barley	[36,60]
Methyl 3-pyridinecarboxylate	93-60-7	[33]	Nauseating, sweet-herbaceous, mildly tobacco-like, fresh, caramel nutty	[34,36]
2-Pentylpyridine	2294-76-0		Tallowy-like	[36]
**Pyrroles**				
1-Methylpyrrole	96-54-8	[33]	Smoky-tarry, sweet, woody-herbaceous, animal, coffee	[20,34]
Pyrrole	109-97-7	[33]	Warm, slightly pungent, hay-like herbaceous, sweet, green, toasted	[20,34,36,54]
1-Ethylpyrrole	617-92-5	[33]	-	
2,5-Dimethylpyrrole	625-84-3	[33]	-	
2-Ethyl-4-methylpyrrole	69687-77-0	[37]	-	
1-Acetylpyrrole	609-41-6	[33]	-	
3-Ethyl-2,4-dimethylpyrrole	517-22-6	[33]	-	
1-Methyl-2-formylpyrrole	1192-58-1	[33]	Cracker-popcorn, burnt	[54,60]
1-Ethyl-2-formylpyrrole	2167-14-8	[33]	-	
2-Acetyl-1-methylpyrrole	932-16-1	[33]	-	
2-Acetylpyrrole	1072-83-9	[33]	Bread, walnut, licorice, cracker, popcorn-like	[20,36,60]
**Sulfur compounds**				
Dimethyl disulfide	624-92-0	[33]	Onion	[34,36]
Methylthio-2-propanone	14109-72-9		Melon	[36]
2-Furylmethylsulfide	13129-38-9	[33]	Garlic-like	[60]
2-Furfurylthiol	98-02-2	[33]	Coffee-like, burnt-caramel-like, sweet, roasty, sulfury	[4,34,39,44,54]
3-(Methylthio)propanal (Methional)	3268-49-3	[33]	Onion, meat-like, bouillon-like, soup-like, cooked potato-like	[4,34,36,38,46]
1-(Methylthio)-2-butanone	13678-58-5	[33]	Mushroom, garlic	[36,60]
Dimethyl trisulfide	3658-80-8	[33]	Fresh onion, cabbage-like, brothy, sulfury, pungent	[4,34,36,37]
3-Mercapto-3-methyl-1-butanol	34300-94-2	[33]	Sweet, soup-like, cooked meat, spicy, smoke-roast, meat, chicken brothy	[37,52,60]
2-Furfuryl methyl sulfide	1438-91-1	[33]	Coffee-like, onion, garlic, burnt, sulfury, cooked cabbage	[20,36,37,60]
3-Mercapto-3-methylbutyl formate	50746-10-6	[33]	Sweaty, fruity, blackcurrant-like, catty, orange flowers, roasty	[4,39,44,52,60]
3-Mercapto-3-methylbutyl acetate	50746-09-3	[65]	-	
Furfuryl methyl disulfide	57500-00-2	[33]	Fresh white bread crust	[60]
**Terpenic compounds**				
*Monoterpenes*				
α-Pinene	80-56-8	[19]	Pine, turpentine-like	[36]
β-Pinene	127-91-3	[19]	Turpentine, dry, woody, resinous	[36]
2,6-Dimethyl-2,6-octadiene (isomer)	2792-39-4		-	
β-Myrcene	123-35-3	[33]	Sweet, balsamic, plastic, sweet-balsamic-resinous gum	[34,36]
2,6-Dimethyl-2,6-octadiene (isomer)	2792-39-4		-	
α-Phellandrene	99-83-2	[66]	Fresh, citrus, peppery, discrete mint, minty, herbaceous note	[36]
α-Terpinene	99-86-5	[19]	Woody, terpene, lemon	[36]
*p*-Cymene	99-87-6	[33]	Carrot-like, kerosene-like	[34,36]
Limonene	138-86-3	[33]	Citrusy, lemon-like, fresh, sweet	[60]
β-Ocimene (isomer)	13877-91-3	[49]	Warm herbaceous	[36]
β-Ocimene (isomer)	13877-91-3	[49]	Warm herbaceous	[36]
γ-Terpinene	99-85-4	[19]	Lemon	[36]
α-Terpinolene	586-62-9		Sweet, pine	[36]
*p*-Cymenene	1195-32-0	[33]	Citrusy-lemon-like, gassy	[34]
Cosmene	460-01-5		-	
*Allo*-ocimene	673-84-7		-	
*Monoterpenoids*				
Linalool oxide (isomer)	1365-19-1	[33]	Sweet, woody, floral, woody-earthy undertone, pungent	[34,36]
Linalool oxide (isomer)	1365-19-1	[33]	Sweet, woody, floral, woody-earthy undertone, pungent	[34,36]
Linalool	78-70-6	[33]	Floral-woody, faintly citrusy note, floral, sweet-fruity	[34,35,36,39,50,52]
α-Terpineol	98-55-5	[33]	Floral, lilac	[60]
Safranal	116-26-7		Saffron-like	[36]
*p*-Menth-1-en-9-al	29548-14-9		-	
*Sesquiterpenes*				
α-Cubebene	17699-14-8		-	
α-Copaene	3856-25-5	[29]	-	
Longifolene	475-20-7		-	
β-Caryophyllene	87-44-5		Cloves, turpentine	[36]
α-Humulene	6753-98-6		-	
α-Muurolene	31983-22-9		-	
δ-Cadinene	483-76-1	[29]	-	
**Norisoprenoids**				
Vitispirane (C_13_)	65416-59-3		-	
Theaspirane (C_13_)	36431-72-8		Fruity, woody, sweetish	[36]
1,2-Dihydro-1,1,6-trimethylnaphthalene (C_13_)	30364-38-6		-	
β-Damascenone (C_13_)	23726-93-4	[33]	Tea-like, fruity, honey-like, fruity, sweet-fruity	[2,39,44,50,52,60,62]
α-Ionone (C_13_)	127-41-3		Warm, woody, berry characteristic violet-like	[36]
Geranyl acetone (C_13_)	689-67-8		Green, rosy floral, fresh-floral, sweet-rosy, slightly green magnolia-like	[36]
**Thiazoles**				
Thiazole	288-47-1	[33]	Green, sweet, nutty, tomato, toasted	[20,36]
2-Methylthiazole	3581-87-1	[33]	-	
4-Methylthiazole	693-95-8	[33]	Nutty, green, roasted	[36,54]
5-Methylthiazole	3581-89-3	[33]	-	
2,4-Dimethylthiazole	541-58-2	[33]	Salty, sulfury, burnt, rubber	[37]
2,5-Dimethylthiazole	4175-66-0	[33]	-	
4,5-Dimethylthiazole	3581-91-7	[33]	Roasted nutty, boiled poultry	[36]
5-Ethylthiazole	17626-73-2	[33]	-	
2-Ethyl-4-methylthiazole	15679-12-6	[33]	Nutty, green	[36]
4-Propylthiazole	41981-60-6		-	
5-Ethyl-2-methylthiazole	19961-52-5	[33]	Rubber-like	[2]
2-Isopropyl-4-methylthiazole	15679-13-7		Green, vegetable, nutty, rooty, earthy	[36]
5-Ethyl-4-methylthiazole	31883-01-9	[33]	Nutty, green, earthy	[60]
2-Acetylthiazole	24295-03-2	[33]	Green onion, herbal, grassy	[36]
4-Ethyl-2,5-dimethylthiazole	32272-57-4	[33]	-	
5-Ethyl-2,4-dimethylthiazole	38205-61-7	[33]	Earthy, roasty	[39]
2-Acetyl-4-methylthiazole	7533-07-5	[33]	-	
2-Propanoyl-thiazole	43039-98-1		-	
Benzothiazole	95-16-9	[33]	Delicate, persistent, rose-like	[36]
**Tiophene compounds**				
Thiophene	110-02-1	[33]	-	
2-Methylthiophene	554-14-3	[33]	Onion, sulfury	[60]
3-Methylthiophene	616-44-4	[33]	-	
2-Ethylthiophene	872-55-9	[47,49]	-	
2,5-Dimethylthiophene	638-02-8	[37]	-	
2,4-Dimethylthiophene	638-00-6		-	
2,3-Dimethylthiophene	632-16-6	[37]	-	
2-Vinylthiophene	1918-82-7	[37]	-	
3-Methoxythiophene	17573-92-1		-	
3-Thiophanone	1003-04-9	[33]	Garlic meaty, green vegetable, buttery	[36]
2-Isopropylthiophene	4095-22-1		-	
2,3,4-Trimethylthiophene	1795-04-6		-	
Dihydro- 2-methyl-3(2H)-thiophenone	13679-85-1	[33]	-	
3-Thiophenecarboxaldehyde	498-62-4		-	
Dihydro-2(3H)-thiophenone	1003-10-7	[33]	-	
2-Thiophenecarboxaldehyde	98-03-3	[33]	Coffee	[20]
3-Methyl-2-thiophenecarboxaldehyde	5834-16-2	[33]	-	
3-Acetylthiophene	1468-83-3	[33]	-	
2-Acetylthiophene	88-15-3	[33]	-	
2,5-Diethylthiophene	5069-23-8		-	
Methyl-2-thiophene carboxylate	5380-42-7	[33]	-	
5-Methyl-2-thiophenecarboxaldehyde	13679-70-4	[33]	-	
2-Pentylthiophene	4861-58-9		-	
2-Propionylthiophene	13679-75-9	[33]	-	

^a^ Compounds previously identified in roasted coffee and/or brews. ^b^ Aroma descriptors and ^c^ corresponding references of each volatile compound, determined in aqueous conditions.

## Supplementary Materials

The following are available online at www.mdpi.com/article/10.3390/foods10102508/s1, Figure S1: Blow-up of the GC × GC-ToFMS chromatograms contour plot obtained in full-scan acquisition mode for the 8 capsule coffee powders (left) and respective espresso brews (right) under study, Figure S2: Mean values of GC × GC peak areas for the compounds analyzed (ordered by chemical family as in Table 1 and Table S1) and corresponding relative standard deviation (RSD) among all samples (8 blends), Figure S3: GC × GC peak areas for the different chemical families determined in the coffee brews considering the blends labeled with different intensities: Blend 1 (intensity 5); Blend 2 (intensity 9), and Blend 3 (intensity 10), Figure S4: Representation of the correlation between coffee powder and coffee brew GC × GC peak areas of the 390 compounds putatively identified through Pearson’s correlation coefficients and p-values associated. Column 1 shows the correlation coefficients of each compound, grouped by its chemical family, illustrated through a chromatic scale shown in column 2, from dark blue (r low values), to dark red (r high values). Column 3 shows the *p*-values of each compound, grouped by its chemical family, illustrated through a chromatic scale shown in column 4, from light green (*p* low values), to dark green (*p* high values), Table S1: Chromatographic details and aroma descriptors of 390 volatile compounds putatively identified in 8 capsule-coffee powders and espresso brews using HS-SPME/GC × GC-ToFMS, Table S2: GC × GC peak areas of 390 volatile compounds putatively identified in 8 roasted ground capsule-coffee powders and respective espresso brews, using HS-SPME/GC × GC-ToFMS.
