# Supplementary material for: Insights on Single-Dose Espresso Coffee Capsules’ Volatile Profile: From Ground Powder Volatiles to Prediction of Espresso Brew Aroma Properties
Source: Foods. 2021 Oct 19;10(10):2508. doi: 10.3390/foods10102508 (PMC8535219; doi:10.3390/foods10102508)
Supplement: Supplementary file 1 [file foods-10-02508-s001.zip › Supplementary Material/Supplementary Material-Figures.pdf]

Supplementary Figures
(Foods)

Insights on single-dose espresso coffee capsules' volatile profile: from ground powder volatiles to prediction of espresso brew aroma properties

Guido R. Lopes [1,†], Sílvia Petronilho [1,2,*,†], Andreia S. Ferreira [1], Mariana Pinto [1], Claúdia P. Passos [1], Elisabete Coelho [1], Carla Rodrigues [3], Cláudia Figueira [4], Sílvia M. Rocha [1] and Manuel A. Coimbra [1]

[1] LAQV-REQUIMTE, Chemistry Department, Campus Universitário de Santiago, University of Aveiro, 3810-193 Aveiro, Portugal; guido@ua.pt (G.R.L.); silviapetronilho@ua.pt (S.P.); a39493@ua.pt (A.S.F.); a39045@ua.pt (M.P.);
cpassos@ua.pt (C.P.P.); ecoelho@ua.pt (E.C.); smrocha@ua.pt (S.M.R.); mac@ua.pt (M.A.C.)

[2] Chemistry Research Centre-Vila Real, Department of Chemistry, University of Trás os-Montes and Alto Douro, Quinta de Prados, 5001-801 Vila Real, Portugal

[3] Delta Ventures, Av. Infante D. Henrique 151-A 1950-405 Lisboa, Portugal; carla.rodrigues@grupo-nabeiro.pt

[4] Diverge, Grupo Nabeiro Innovation Center, Alameda dos Oceanos 65, 1.1, 1990-208 Lisboa, Portugal; claudia.figueira@gruponabeiro.com

* Correspondence: silviapetronilho@ua.pt

† G.R.L. and S.P. contributed equally as first authors.

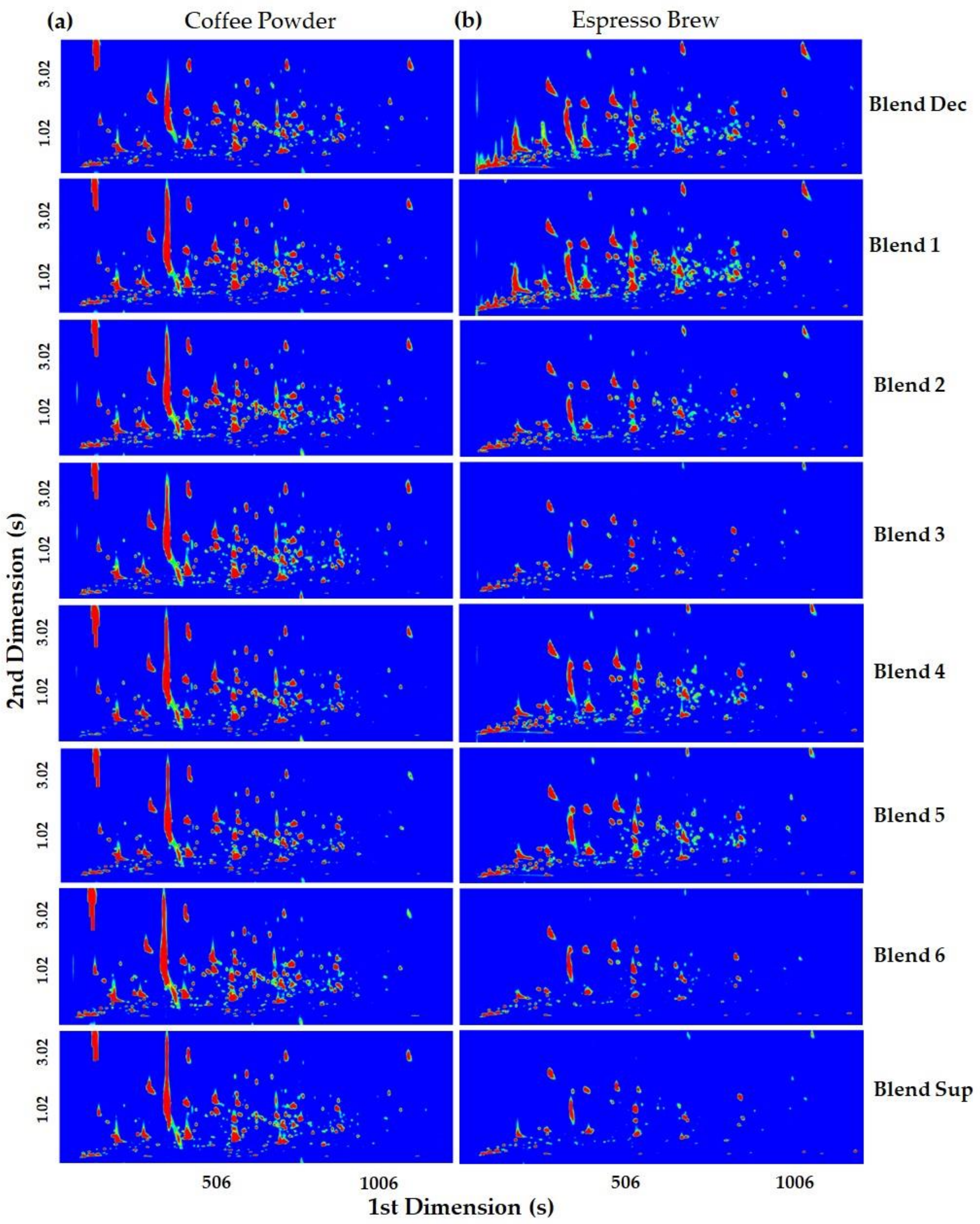

Figure S1. Blow-up of the GC × GC-ToFMS chromatograms contour plot obtained in full-scan acquisition mode for the 8 capsule coffee powders (a) and respective espresso brews (b) under study.

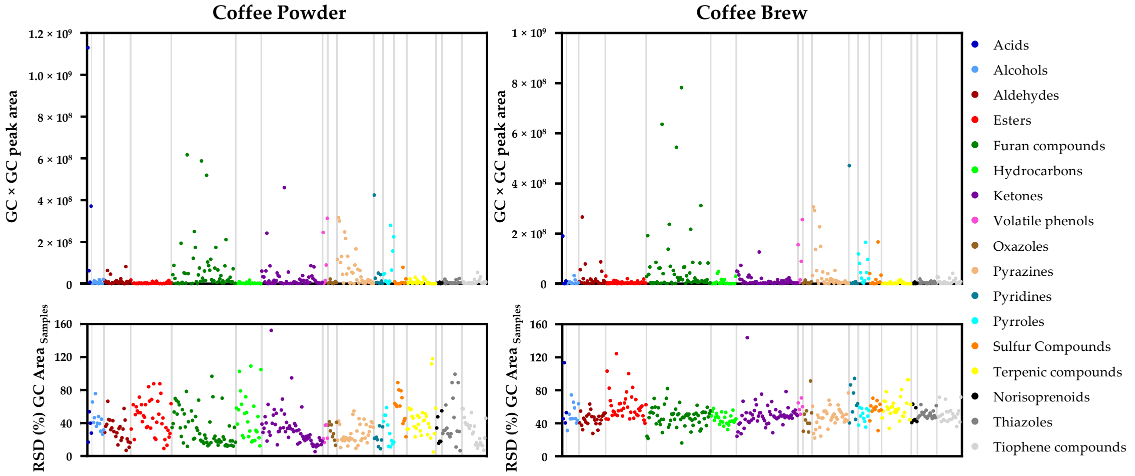

Figure S2. Mean values of GC × GC peak areas for the compounds analyzed (ordered by chemical family as in Table 1 and S1) and corresponding relative standard deviation (RSD) among all samples (8 blends).

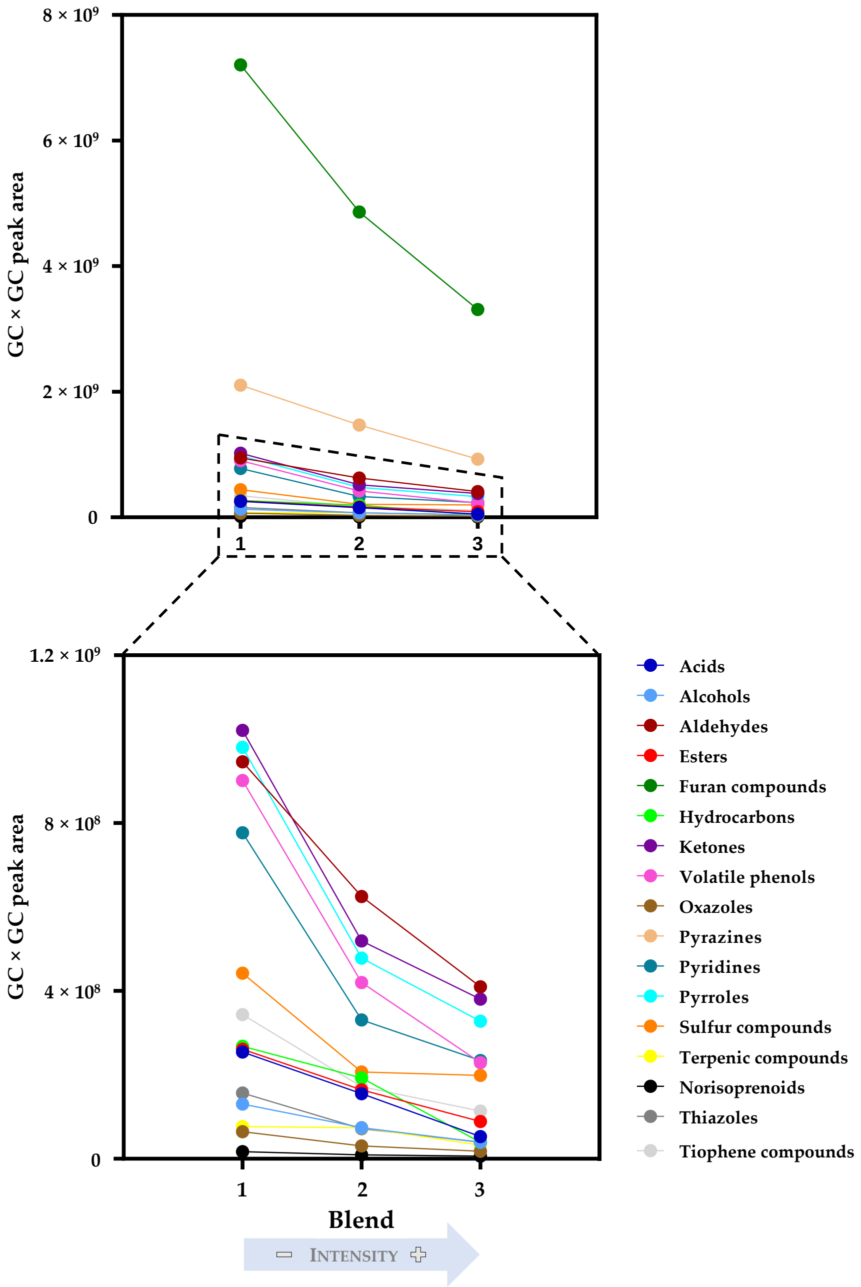

Figure S3. GC × GC peak areas for the different chemical families determined in the coffee brews considering the blends labeled with different intensities: Blend 1 (intensity 5); Blend 2 (intensity 9), and Blend 3 (intensity 10).

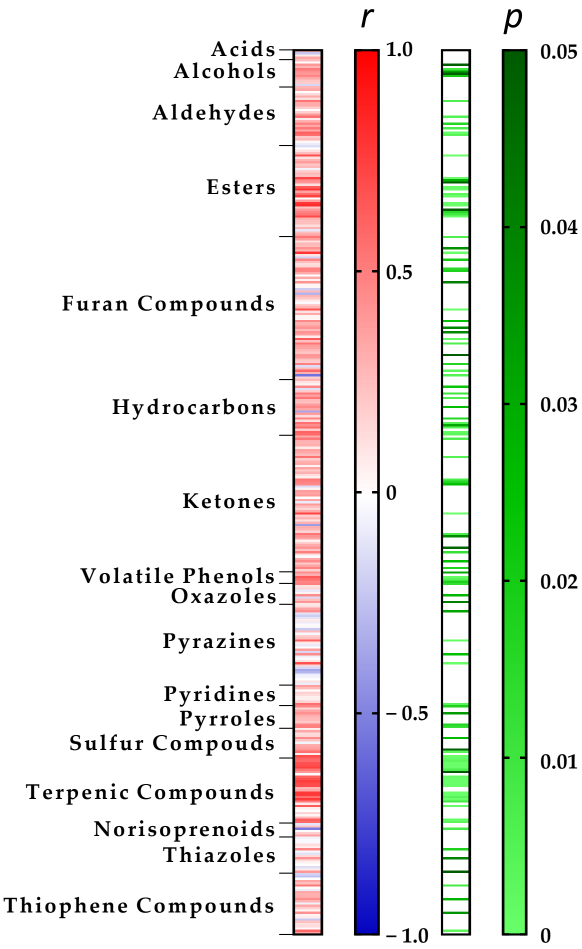

Figure S4. Representation of the correlation between coffee powder and coffee brew GC × GC peak areas of the 390 compounds putatively identified through Pearson's correlation coefficients and *p*-values associated. Column 1 shows the correlation coefficients of each compound, grouped by its chemical family, illustrated through a chromatic scale shown in column 2, from dark blue (*r* low values), to dark red (*r* high values). Column 3 shows the *p*-values of each compound, grouped by its chemical family, illustrated through a chromatic scale shown in column 4, from light green (*p* low values), to dark green (*p* high values).
