# Supplementary material for: Insights on Single-Dose Espresso Coffee Capsules’ Volatile Profile: From Ground Powder Volatiles to Prediction of Espresso Brew Aroma Properties
Source: Foods. 2021 Oct 19;10(10):2508. doi: 10.3390/foods10102508 (PMC8535219; doi:10.3390/foods10102508)
Supplement: Supplementary file 1 [file foods-10-02508-s001.zip › Supplementary Material/Supplementary Material-Table S1.pdf]

Supplementary Table S1

(Foods)

Insights on single-dose espresso coffee capsules' volatile profile: from ground powder volatiles to prediction of espresso brew aroma properties

Guido R. Lopes [1,†], Sílvia Petronilho [1,2,*,†], Andreia S. Ferreira [1], Mariana Pinto [1], Claúdia P. Passos [1], Elisabete Coelho [1], Carla Rodrigues [3], Cláudia Figueira [4], Sílvia M. Rocha [1] and Manuel A. Coimbra [1]

[1] LAQV-REQUIMTE, Chemistry Department, Campus Universitário de Santiago, University of Aveiro, 3810-193 Aveiro, Portugal; guido@ua.pt (G.R.L.); silviapetronilho@ua.pt (S.P.); a39493@ua.pt (A.S.F.); a39045@ua.pt (M.P.);
cpassos@ua.pt (C.P.P.); ecoelho@ua.pt (E.C.); smrocha@ua.pt (S.M.R.); mac@ua.pt (M.A.C.)

[2] Chemistry Research Centre-Vila Real, Department of Chemistry, University of Trás os-Montes and Alto Douro, Quinta de Prados, 5001-801 Vila Real, Portugal

[3] Delta Ventures, Av. Infante D. Henrique 151-A 1950-405 Lisboa, Portugal; carla.rodrigues@grupo-nabeiro.pt

[4] Diverge, Grupo Nabeiro Innovation Center, Alameda dos Oceanos 65, 1.1, 1990-208 Lisboa, Portugal; claudia.figueira@gruponabeiro.com

* Correspondence: silviapetronilho@ua.pt

† G.R.L. and S.P. contributed equally as first authors.

Table S1. Chromatographic details and aroma descriptors of 390 volatile compounds putatively identified in 8 capsule-coffee powders and espresso brews using HS-SPME/GC × GC-ToFMS.

Peak num	$^{1}t_R$ (s) [a]	$^{2}t_R$ (s) [a]	Compound	CAS number	Formula	RI_{Calc} [b]	RI_{Lit} [c]	RI Ref.	Previously reported[d]	Aroma descriptor[e]	Aroma descriptor Ref.
			**Acids**								
			Aliphatics								
1	144	5.370	Acetic acid	64-19-7	$C_2H_4O_2$	672	648	[1]	[91]	Pungent, sour, acidic, vinegar	[109, 114-116]
2	180	1.660	Propanoic acid	79-09-4	$C_3H_6O_2$	721	699	[2]	[91]	Pungent, rancid, sour milk, cheese, butter-like	[114, 116]
3	270	2.980	Butanoic acid	107-92-6	$C_4H_8O_2$	828	815	[1]	[91]	Sour, rancid, butter-like, sweaty, rubbish	[98, 114, 116, 117]
4	342	2.030	Isovaleric acid	503-74-2	$C_5H_{10}O_2$	880	880	[3]	[91]	Acidic, cheesy, herbaceous, sweaty, rancid	[98, 114-116, 118]
			**Alcohols**								
			Aliphatics								
5	108	0.850	2-Methyl-1-propanol	78-83-1	$C_4H_{10}O$	610	612	[4]	[91]	Wine-like	[116]
6	114	1.240	3-Buten-1-ol	627-27-0	C_4H_8O	620	-	-		-	
7	174	1.460	3-Methyl-3-buten-1-ol	763-32-6	$C_5H_{10}O$	711	708	[4]	[91]	-	
8	180	1.180	2-Methyl-1-butanol	137-32-6	$C_5H_{12}O$	720	728	[1]	[92]	Cooked, roasted with fruity or alcoholic undernotes	[116]
9	210	1.340	1-Pentanol	71-41-0	$C_5H_{12}O$	766	767	[3]	[91]	Green, chemical, fusel oil-like sweet	[114, 116, 119]
10	222	1.800	3-Methyl-2-buten-1-ol	556-82-1	$C_5H_{10}O$	785	782	[2]	[91]	Fresh, herbaceous-fruity-green, lavender-like, phenolic, metallic	[114, 116]
11	246	1.060	2-Hexanol	626-93-7	$C_6H_{14}O$	809	813	[5]	[91]	-	
12	384	1.110	2-Heptanol	543-49-7	$C_7H_{16}O$	908	906	[2]	[91]	Fresh, lemon-like, grassy-herbaceous, sweet-floral undertone	[114, 116]
13	516	1.360	1-Octen-3-ol	3391-86-4	$C_8H_{16}O$	984	980	[4]	[91]	Mushroom, herbaceous, savoury, brothy, meaty	[98, 119, 120]
14	600	1.280	2-Ethyl-1-hexanol	104-76-7	$C_8H_{18}O$	1032	1033	[2]	[93]	Sweet, slightly floral rose-like	[116]
15	678	1.360	1-Octanol	111-87-5	$C_8H_{18}O$	1077	1079	[4]	[94]	Fresh, orange-rose, sweet	[116]
			Aromatics								
16	756	4.550	2-Phenylethanol	60-12-8	$C_8H_{10}O$	1124	1121	[2]	[91]	Rose-honey-like, floral	[114, 116, 119]
			**Aldehydes**								
			Aliphatics								
17	66	0.350	Acetaldehyde	75-07-0	C_2H_4O	546	548	[4]	[91]	Pungent, ethereal, fruity, coffee, wine, acrid/egg,	[114, 116, 119, 121, 122]
18	90	0.430	2-Methylpropanal	78-84-2	C_4H_8O	582	582	[6]	[91]	Pungent, sour, fruity, malty, buttery-oily	[114, 116, 123]
19	120	0.710	2-Butenal	4170-30-3	C_4H_6O	628	633	[4]	[95, 96]	-	
20	126	0.460	3-Methylbutanal	590-86-3	$C_5H_{10}O$	637	633	[4]	[91]	Pungent, acrid, fruity, apple-like, almond, malty, sweaty	[114, 116, 118, 121, 123]
21	180	0.770	2-Methyl-2-butenal	1115-11-3	C_5H_8O	719	715	[7]	[91]	-	
22	192	0.850	2-Pentenal	764-39-6	C_5H_8O	738	726	[8]		Pungent, green, apple, orange, tomato	[116]
23	240	0.660	Hexanal	66-25-1	$C_6H_{12}O$	805	801	[4]	[91]	Fatty, green, grassy, fruity, rancid butter-like, nutty	[114, 116, 117, 119, 123]
24	258	0.800	4-Methyl-3-pentenal	5362-50-5	$C_6H_{10}O$	818	-	-		-	
25	336	0.750	2-Methyl-2-hexenal	28467-88-1	$C_7H_{12}O$	874	884	[9]		-	
26	336	1.350	2,4-Hexadienal	80466-34-8	C_6H_8O	875	879	[10]	[91]	Fresh, green, floral, citrus	[116]
27	342	0.690	4-Methylhexanal	41065-97-8	$C_7H_{14}O$	879	889	[9]		-	
28	378	0.710	Heptanal	111-71-7	$C_7H_{14}O$	904	906	[3]	[91]	Oily-fatty, rancid, pungent, fermented-fruit-like	[114, 116]
29	474	0.960	2-Heptenal	2463-63-0	$C_7H_{12}O$	959	959	[4]	[94]	Pungent, green, fatty	[116]
30	552	0.740	Octanal	124-13-0	$C_8H_{16}O$	1004	1001	[4]	[91]	Fatty, citrus, orange-like, honey	[116, 126]
31	594	1.310	2,4-Heptadienal	5910-85-0	$C_7H_{10}O$	1028	1043	[11]	[97]	Fatty, green ((*E,E*)-isomer)	[116]
32	648	1.000	2-Octenal	2363-89-5	$C_8H_{14}O$	1059	1059	[2]	[98]	Green-leafy, orange, honey-like, cognac-like	[98, 116]
33	726	0.770	Nonanal	124-19-6	$C_9H_{18}O$	1104	1106	[4]	[94, 99]	Fatty, orange and rose note, soap-like, metallic	[116, 125]
34	822	0.950	2-Nonenal	2463-53-8	$C_9H_{16}O$	1161	1162	[4]	[91]	Fatty, orris-like, waxy, dried orange peels-like, cardboard-like	[116, 117]
35	900	0.780	Decanal	112-31-2	$C_{10}H_{20}O$	1208	1207	[4]	[97, 99, 100]	Sweet, waxy, floral, citrus, fatty	[116]
36	1068	0.730	Undecanal	112-44-7	$C_{11}H_{22}O$	1309	1307	[4]	[100]	Sweet, fatty, orange and rose undertone	[116]
37	1224	0.780	Dodecanal	112-54-9	$C_{12}H_{24}O$	1413	1407	[4]	[97, 100]	Fatty, violet-like	[116]
			Aromatics								
38	474	2.150	Benzaldehyde	100-52-7	C_7H_6O	960	965	[4]	[91]	Sweet, bitter almond-like, bitter	[114, 116, 119]
39	618	2.380	Benzeneacetaldehyde	122-78-1	C_8H_8O	1043	1046	[4]	[91]	Pungent-green, hyacinth-like, floral, sweet-fruity, honey-like	[114, 116, 118, 124, 125]
40	618	2.750	2-Hydroxybenzaldehyde	90-02-8	$C_7H_6O_2$	1043	1044	[2]	[91]	Pungent, herbaceous, spicy-floral, bitter, almond-like	[114, 116]
41	660	1.960	2-Methylbenzaldehyde	529-20-4	C_8H_8O	1067	1074	[4]	[91]	Sweet, beany, fresh pea	[98, 114]
42	1008	2.260	2-Phenyl-2-butenal	4411-89-6	$C_{10}H_{10}O$	1273	1278	[2]	[91]	Musty, floral, cocoa	[128]
			**Esters**								
			Aliphatics								
43	84	0.420	Methyl acetate	79-20-9	$C_3H_6O_2$	573	566	[3]	[91]	Sweet, ethereal, fruity	[114, 116]
44	102	0.500	Methyl propenoate	96-33-3	$C_4H_6O_2$	601	607	[12]		-	
45	108	0.470	Methyl propanoate	554-12-1	$C_4H_8O_2$	610	615	[13]	[91]	Ethereal-rum-like, sweet, fruity	[114, 116]
46	150	3.410	Methyl glycolate	96-35-5	$C_3H_6O_3$	678	-	-	[95]	-	
47	156	0.570	Methyl 2-methylpropenoate	80-62-6	$C_5H_8O_2$	683	685	[4]		Acrid, fruity	[116]
48	162	0.540	Methyl butanoate	623-42-7	$C_5H_{10}O_2$	692	720	[13]	[91]	Sweet-ethereal, fruity, apple peel, peach-like	[114, 116]

49	198	0.770	Methyl 2-butenoate	18707-60-3	$C_5H_8O_2$	747	755	[14]	[96]	-	
50	210	0.530	Methyl 2-methylbutanoate	868-57-5	$C_6H_{12}O_2$	764	770	[15]		Sweet, fruity, apple-like	[116]
51	216	0.570	Methyl 3-methylbutanoate	556-24-1	$C_6H_{12}O_2$	774	775	[12]	[91]	Ethereal, fruity, apple-like, herbaceous	[114, 116]
52	228	0.650	3-Methylbutyl formate	110-45-2	$C_6H_{12}O_2$	792	791	[2]	[98]	Plum, fruity, black currant-like	[98, 116]
53	258	0.620	Butyl acetate	123-86-4	$C_6H_{12}O_2$	818	818	[4]	[91]	Pear-like, ethereal, fruity, ripe/over-ripe fruits-like	[114, 116]
54	270	0.610	Methyl pentanoate	624-24-8	$C_6H_{12}O_2$	827	829	[5]		Ethereal, green-fruity, apple-like, pineapple- like	[114, 116]
55	294	0.810	Methyl 3-methyl-2-butenoate	924-50-5	$C_6H_{10}O_2$	844	842	[16]	[101]	roasted	[129]
56	312	0.570	Ethyl 3-methylbutanoate	108-64-5	$C_7H_{14}O_2$	857	857	[3]	[91]	Fruity, apple-like	[116, 117, 119]
57	348	0.630	Isoamyl acetate	123-92-2	$C_7H_{14}O_2$	883	882	[3]	[91]	Fruity, banana, sweet, apple-like	[114, 116]
58	354	0.620	Methyl 3-methylpentanoate	2177-78-8	$C_7H_{14}O_2$	887	-	-		-	
59	360	0.780	3-Methyl-3-butenyl acetate	5205-07-2	$C_7H_{12}O_2$	892	881	[17]		Fruity	[116]
60	366	0.630	Methyl 4-methylpentanoate	2412-80-8	$C_7H_{14}O_2$	896	900	[18]		Sweet, pineapple-like	[116]
61	402	0.660	Pentyl acetate	628-63-7	$C_7H_{14}O_2$	918	916	[3]		-	
62	420	0.660	Methyl hexanoate	106-70-7	$C_7H_{14}O_2$	928	927	[3]	[91]	Pineapple-like, apricot-like, sweet, ethereal	[114, 116]
63	420	0.750	Ethyl tiglate	5837-78-5	$C_7H_{12}O_2$	928	929	[19]		Fruity, caramel	[114, 116]
64	420	0.820	3-Methyl-2-butenyl acetate	1191-16-8	$C_7H_{12}O_2$	928	929	[20]	[91]	Fresh, fruity, banana-like, bergamot-like	[114]
65	426	0.730	Hexyl formate	629-33-4	$C_7H_{14}O_2$	931	927	[21]		Fruity, apple-like, unripe-plum	[116]
66	456	1.530	Ethylidene acetate	542-10-9	$C_6H_{10}O_4$	949	-	-		-	
67	504	1.190	Methyl 4-methyl-2-oxopentanoate	3682-43-7	$C_7H_{12}O_3$	977	-	-		-	
68	516	0.640	Butyl 2-methyl-2-propenoate	97-88-1	$C_8H_{14}O_2$	983	989	[22]		-	
69	570	0.680	Hexyl acetate	142-92-7	$C_8H_{16}O_2$	1014	1006	[3]	[102]	Fruity, apple, cherry, pear, floral	[116]
70	684	0.710	Isobutyl 3-methyl-2-butenoate	30434-54-9	$C_9H_{16}O_2$	1080	-	-		-	
71	768	0.720	Methyl octanoate	111-11-5	$C_9H_{18}O_2$	1129	1130	[3]		Winy, fruity, orange-like	[116]
72	864	0.720	3-Methylbutyl 3-methyl-2-butenoate	56922-73-7	$C_{10}H_{18}O_2$	1186	1184	[23]		-	
73	1092	0.680	Methyl decanoate	110-42-9	$C_{11}H_{22}O_2$	1325	1332	[3]		-	
74	1392	0.680	Methyl dodecanoate	111-82-0	$C_{13}H_{26}O_2$	1531	1527	[5]		Fatty, floral, wine-like	[116]
			Aromatics								
75	654	1.990	Phenyl acetate	122-79-2	$C_8H_8O_2$	1063	1068	[18]	[103, 104]	Floral, rosy, dark chocolate-like	[128]
76	678	2.310	Benzyl formate	104-57-4	$C_8H_8O_2$	1077	1077	[5]	[91]	Fruity, green, herbaceous, earthy, floral	[114, 116]
77	708	1.710	Methyl benzoate	93-58-3	$C_8H_8O_2$	1094	1096	[2]	[91]	Fruity, cananga-like	[116]
78	852	1.920	Methyl phenylacetate	101-41-7	$C_9H_{10}O_2$	1180	1179	[5]	[91]	Honey, musky, jasmine, floral	[114, 116]
79	852	2.110	2-Phenylethyl formate	104-62-1	$C_9H_{10}O_2$	1180	1176	[5]	[91]	Green, herbaceous, rosy, hyacinth, chrysanthemum, watercress-	[114, 116]
80	882	1.880	Methyl 2-hydroxybenzoate	119-36-8	$C_8H_8O_3$	1198	1207	[3]	[91]	Sweet, rooty-fruity, minty, spicy, wintergreen-like	[114, 116]
81	990	1.540	2-Phenylethyl acetate	103-45-7	$C_{10}H_{12}O_2$	1262	1260	[4]	[105]	Floral, rose, honey-like	[116]
82	1002	1.550	Ethyl 2-hydroxybenzoate	118-61-6	$C_9H_{10}O_3$	1269	1280	[24]	[96]	Wintergreen	[116]
			**Furan compounds**								
83	78	0.380	Furan	110-00-9	C_4H_4O	564	567	[25]	[91]	Spicy-smokey, cinnamon-like	[114]
84	102	0.460	2-Methylfuran	534-22-5	C_5H_6O	601	601	[26]	[91]	Ethereal, sickly	[109]
85	108	0.420	Tetrahydrofuran	109-99-9	C_4H_8O	610	634	[27]	[91]	Sweet-gassy, bread-like	[114]
86	150	0.500	2,5-Dimethylfuran	625-86-5	C_6H_8O	703	704	[28]	[91]	coffee	[109]
87	174	0.580	2,4-Dimethylfuran	3710-43-8	C_6H_8O	710	735	[29]	[96]	-	
88	228	0.570	2-Propylfuran	4229-91-8	$C_7H_{10}O$	792	788	[12]	[91]	-	
89	240	0.570	2-Ethyl-5-methylfuran	1703-52-2	$C_7H_{10}O$	805	799	[28]	[91]	-	
90	246	0.470	2-Ethyl-5-methyltetrahydrofuran	931-39-5	$C_7H_{14}O$	809	-	-		-	
91	246	2.470	2-Furancarbonitrile	617-90-3	C_5H_3NO	810	822	[30]		-	
92	252	1.290	Dihydro-2-methyl-3-furanone	3188-00-9	$C_5H_8O_2$	814	813	[2]	[91]	Bread-like, buttery, nutty	[109, 116]
93	258	0.590	2,3,5-Trimethylfuran	10504-04-8	$C_7H_{10}O$	818	817	[31]	[91]	-	
94	264	2.840	3-Furaldehyde	498-60-2	$C_5H_4O_2$	824	831	[32]	[91]	-	
95	276	0.760	2-Vinyl-5-methylfuran	10504-13-9	C_7H_8O	831	841	[31]	[91]	Coffee	[109]
96	276	1.100	2-(Methoxymethyl)furan	13679-46-4	$C_6H_8O_2$	831	-	-	[91]	Burnt, herbal, potato-like	[98, 126]
97	282	0.630	2,3,4-Trimethylfuran	10599-57-2	$C_7H_{10}O$	835	-	-	[91]	-	
98	282	3.300	Furfural	98-01-1	$C_5H_4O_2$	837	839	[1]	[91]	Sweet, bread-like, caramellic, cinnamon-almond-like, bitter	[109, 114]
99	312	0.900	2-(2-Propenyl)furan	75135-41-0	C_7H_8O	857	856	[28]	[91]	-	
100	336	2.190	5-Methyl-2(3H)-furanone	591-12-8	$C_5H_6O_2$	876	874	[33]	[98, 106]	Sweet, herbaceous, tobacco-like, coffee, earthy, raw potato skin	[98, 109, 116]
101	342	2.010	2-Furanmethanol	98-00-0	$C_5H_6O_2$	880	867	[3]	[91]	Slightly caramellic, warm, oily, burnt, bitter	[98, 114, 116, 119]
102	360	0.470	2,5-Diethyltetrahydrofuran	41239-48-9	$C_8H_{16}O$	892	896	[16]		Sweet, herbaceous, caramellic	[116]
103	366	0.610	2-Butylfuran	4466-24-4	$C_8H_{12}O$	896	898	[29]	[91]	-	
104	390	2.700	Furfuryl formate	13493-97-5	$C_6H_6O_3$	912	902	[34]	[91]	Floral	[109]
105	402	2.530	2-Acetylfuran	1192-62-7	$C_6H_6O_2$	919	917	[2]	[91]	Balsamic-sweet, tobacco-like, floral, balsamic-cinnamic, spicy,	[109, 114, 116, 126]
106	414	4.220	γ-Butyrolactone	96-48-0	$C_4H_6O_2$	927	924	[4]	[91]	Sweet, slightly buttery	[109, 114, 116]
107	432	0.660	2,3,4,5-Tetramethylfuran	10599-58-3	$C_8H_{12}O$	935	-	-	[91]	-	
108	450	1.100	3-(1,1-Dimethylethyl)-2,3-dihydrofuran	34314-82-4	$C_8H_{14}O$	945	-	-		-	
109	468	2.200	1-(2-Furyl)-2-propanone	6975-60-6	$C_7H_8O_2$	956	963	[26]	[91]	Sweet, fruity-caramellic, spicy, radish	[114, 116]

110	474	0.840	2-Methyl-5-propenylfuran	5555-95-3	$C_8H_{10}O$	959	972	[31]	[91]	Candy, fruity, sweet	[98]
111	480	3.090	Dihydro-5-methyl-2(3H)-furanone	108-29-2	$C_5H_8O_2$	964	965	[30]	[91]	Sweet, hay-like, tobacco-like, herbaceous	[114, 116]
112	486	2.700	5-Methylfurfural	620-02-0	$C_6H_6O_2$	967	965	[3]	[91]	Sweet, spicy, caramel	[109, 114, 116, 119]
113	504	1.610	2-Acetyl-5-methylfuran	1193-79-9	$C_7H_8O_2$	977	977	[35]	[91]	Nutty	[116]
114	510	2.510	Methyl 2-furoate	611-13-2	$C_6H_6O_3$	981	983	[36]	[91]	Berry-like, fruity, winey, mushrooms-like, fungus-like, tobacco-like	[114, 116]
115	534	0.660	2-Pentylfuran	3777-69-3	$C_9H_{14}O$	993	992	[36]	[91]	Fruity, green bean, metallic, vegetable	[116]
116	534	1.670	Benzofuran	271-89-6	C_8H_6O	994	996	[3]	[91]	-	
117	540	1.940	Furfuryl acetate	623-17-6	$C_7H_8O_3$	998	998	[37]	[91]	Ethereal-floral, herbal-spicy, fruity, banana, nutty	[109, 114, 116]
118	552	2.930	2,5-Dihydro-3,5-dimethyl-2-furanone	5584-69-0	$C_6H_8O_2$	1005	993	[38]	[99, 107]	-	
119	564	2.070	1-(2-Furanyl)-1-propanone	3194-15-8	$C_7H_8O_2$	1012	1014	[27]	[91]	-	
120	612	2.030	2,2'-Bifuran	5905-00-0	$C_8H_6O_2$	1039	1044	[28]	[91]	-	
121	624	1.670	3,4-Dimethyl-2,5-furandione	766-39-2	$C_6H_6O_3$	1046	1046	[27]	[91]	-	
122	630	1.720	1-(5-Methyl-2-furyl)-2-propanone	13678-74-5	$C_8H_{10}O_2$	1049	1047	[39]	[91]	-	
123	642	1.810	1-(2-Furanyl)-2-butanone	4208-63-3	$C_8H_{10}O_2$	1056	1063	[26]	[91]	-	
124	648	2.100	5-Ethyl-2-furaldehyde	23074-10-4	$C_7H_8O_2$	1060	1058	[27]	[91]	-	
125	678	2.050	1-(2-Furanyl)-3-butanone	699-17-2	$C_8H_{10}O_2$	1077	1077	[33]	[91]	-	
126	690	1.720	2,2′-Methylenebisfuran	1197-40-6	$C_9H_8O_2$	1084	1084	[33]	[91]	-	
127	696	1.480	3-Acetyl-2,5-dimethylfuran	10599-70-9	$C_8H_{10}O_2$	1087	1089	[27]		-	
128	702	1.580	Furfuryl propanoate	623-19-8	$C_8H_{10}O_3$	1091	1096	[28]	[91]	Spicy, floral, fruity	[116, 130]
129	720	1.810	1-(2-Furanyl)-1-butanone	4208-57-5	$C_8H_{10}O_2$	1101	-	-	[91]	-	
130	732	1.410	2-Methylbenzofuran	4265-25-2	C_9H_8O	1108	1108	[27]	[91]	-	
131	774	1.730	2-Methyl-5-propionylfuran	10599-69-6	$C_8H_{10}O_2$	1133	1136	[27]	[91]	-	
132	798	1.450	1-(5-Methyl-2-furanyl)-2-butanone	13678-70-1	$C_9H_{12}O_2$	1147	-	-	[91]	-	
133	834	2.220	2-Heptylfuran	3777-71-7	$C_{11}H_{18}O$	1169	1154	[3]	[91]	Roasted, nutty	[116]
134	846	1.410	Furfuryl butanoate	623-21-2	$C_9H_{12}O_3$	1173	1171	[40]	[91]	-	
135	858	1.430	2-(2-Furanylmethyl)-5-methylfuran	13678-51-8	$C_{10}H_{10}O_2$	1183	1179	[33]	[91]	-	
136	864	2.410	*n*-Furfuryl pyrrole	1438-94-4	C_9H_9NO	1187	1189	[28]	[91]	Vegetable, green, earthy, horseradish, mushroom-like	[116, 130]
137	876	2.640	2-Methyl-3(2-furyl)acrolein	874-66-8	$C_8H_8O_2$	1194	1196	[25]		-	
138	876	2.970	4-(2-Furanyl)-3-buten-2-one	623-15-4	$C_8H_8O_2$	1195	1191	[25]	[91]	Spicy-woody, sweet, cinnamon-like, balsamic, vanilla, woody	[109, 116]
139	906	1.340	4,7-Dimethylbenzofuran	28715-26-6	$C_{10}H_{10}O$	1212	1218	[25]		-	
140	990	2.780	2-Furyl pyrazine	32736-95-1	$C_8H_6N_2O$	1262	-	-	[91]	-	
141	1020	1.220	2,2′-Methylenebis(5-methylfuran)	13679-43-1	$C_{11}H_{12}O_2$	1279	1277	[33]	[91]	-	
142	1020	1.870	1-(5-Methylfurfuryl)pyrrole	13678-52-9	$C_{10}H_{11}NO$	1280	-	-	[91]	Mushroom-like, green, pharmaceutical, roasty	[126, 130]
143	1056	2.410	Difurfuryl ether	4437-22-3	$C_{10}H_{10}O_3$	1302	1305	[28]	[91]	-	
144	1152	1.700	1-Furfuryl-2-formylpyrrole	13788-32-4	$C_{10}H_9NO_2$	1365	-	-	[91]	green, minty	[109]
145	1326	2.540	2-Acetyl-1-furfurylpyrrole	13678-73-4	$C_{11}H_{11}NO_2$	1485	-	-	[91]	-	
			**Hydrocarbons**								
			Aliphatics								
146	378	0.380	Nonane	111-84-2	C_9H_{20}	904	900	[4]	[91]	-	
147	420	0.500	1,3-Nonadiene	-	C_9H_{16}	928	930	[41]		-	
148	546	0.400	Decane	124-18-5	$C_{10}H_{22}$	1000	1000	[4]	[91]	-	
149	720	0.410	Undecane	1120-21-4	$C_{11}H_{24}$	1100	1101	[2]	[91]	-	
150	882	0.750	1-Dodecene	112-41-4	$C_{12}H_{24}$	1197	1192	[42]		-	
151	894	0.430	Dodecane	112-40-3	$C_{12}H_{26}$	1204	1201	[4]	[91]	-	
152	1056	0.460	Tridecane	629-50-5	$C_{13}H_{28}$	1300	1301	[4]	[91]	-	
153	1212	0.470	Tetradecane	629-59-4	$C_{14}H_{30}$	1405	1401	[4]	[91]	-	
154	1356	0.450	Pentadecane	629-62-9	$C_{15}H_{32}$	1505	1501	[4]	[91]	-	
155	1494	0.470	Hexadecane	544-76-3	$C_{16}H_{34}$	1605	1601	[4]	[91]	-	
			Aromatics								
156	204	0.600	Methylbenzene	108-88-3	C_7H_8	755	759	[4]	[91]	Sweet-gassy	[114]
157	318	0.680	Ethylbenzene	100-41-4	C_8H_{10}	861	860	[4]	[91]	Sweet-gassy	[114]
158	330	0.690	1,3-Dimethylbenzene	108-38-3	C_8H_{10}	870	871	[4]	[91]	-	
159	360	0.950	Phenylethylene	100-42-5	C_8H_8	892	895	[2]	[91]	Sweet-gassy, balsamic, floral	[114, 116]
160	414	0.640	1-Methylethylbenzene	98-82-8	C_9H_{12}	925	927	[4]		-	
161	474	0.690	1-Ethyl-4-methylbenzene	622-96-8	C_9H_{12}	959	964	[4]	[91]	-	
162	582	0.820	1,2,3-Trimethylbenzene	526-73-8	C_9H_{12}	1021	1021	[2]		-	
163	630	0.700	1-Methyl-3-propylbenzene	1074-43-7	$C_{10}H_{14}$	1049	1056	[4]		-	
164	642	0.690	Butylbenzene	104-51-8	$C_{10}H_{14}$	1056	1054	[21]		-	
165	678	0.740	4-Ethyl-1,2-dimethylbenzene	934-80-5	$C_{10}H_{14}$	1076	1082	[43]		-	
166	690	0.780	1-Ethyl-2,3-dimethylbenzene	933-98-2	$C_{10}H_{14}$	1083	1085	[2]		-	
167	750	0.820	1,2,4,5-Tetramethylbenzene	95-93-2	$C_{10}H_{14}$	1118	1123	[4]	[91]	-	
168	804	0.870	1,2,3,4-Tetramethylbenzene	488-23-3	$C_{10}H_{14}$	1151	1150	[44]	[108]	-	
169	816	0.690	Pentylbenzene	538-68-1	$C_{11}H_{16}$	1158	1154	[21]		-	

170	1542	0.600	1-Butylheptylbenzene	4537-15-9	$C_{17}H_{28}$	1641	1633	[11]		-	
			**Ketones**								
			Aliphatics								
171	78	0.410	2-Propanone	67-64-1	C_3H_6O	564	559	[4]	[91]	Ethereal, lemon	[114, 119]
172	96	0.500	2-Butanone	78-93-3	C_4H_8O	592	590	[4]	[91]	Ethereal, sweet apricot-like	[114, 116]
173	96	0.600	2,3-Butanedione (Diacetyl)	431-03-8	$C_4H_6O_2$	592	591	[4]	[91]	Buttery	[114-116, 121, 123]
174	138	0.560	2-Pentanone	107-87-9	$C_5H_{10}O$	655	664	[4]	[91]	Ethereal-fruity, wine	[114, 116]
175	144	0.710	2,3-Pentanedione	600-14-6	$C_5H_8O_2$	665	665	[4]	[91]	Buttery, oily, sweet, caramel-like, milky	[98, 109, 114-116, 121, 126]
176	168	1.910	1-Hydroxy-2-propanone	116-09-6	$C_3H_6O_2$	703	698	[45]	[91]	Sweet-caramellic, mushroom, earthy, nutty	[98, 109, 114]
177	180	0.560	4-Methyl-2-pentanone	108-10-1	$C_6H_{12}O$	719	721	[2]	[91]	Ethereal-fruity, spicy	[114, 116]
178	180	0.860	3-Penten-2-one	625-33-2	C_5H_8O	719	717	[4]	[91]	Fruity	[116]
179	192	1.660	3-Hydroxy-2-butanone (Acetoin)	513-86-0	$C_4H_8O_2$	739	733	[46]	[91]	Creamy-fatty-buttery, woody, yogurt	[109, 114, 116, 119]
180	204	0.640	2-Methyl-1-penten-3-one	25044-01-3	$C_6H_{10}O$	756	748	[47]		-	
181	216	0.910	2,4-Pentanedione	123-54-6	$C_5H_8O_2$	774	783	[36]	[91]	Ethereal-minty, metallic	[114]
182	222	0.620	3-Hexanone	589-38-8	$C_6H_{12}O$	783	785	[18]	[91]	Ethereal, grape, wine-like	[98]
183	222	0.770	2,4-Dimethyl-3-pentanone	565-80-0	$C_7H_{14}O$	783	793	[48]	[91]	-	
184	228	0.670	2-Hexanone	591-78-6	$C_6H_{12}O$	792	791	[2]	[91]	-	
185	240	0.790	3,4-Hexanedione	4437-51-8	$C_6H_{10}O_2$	805	804	[28]	[91]	Buttery, toasty, burnt, nutty, caramel-eggy	[114, 116]
186	240	2.220	1-Hydroxy-2-butanone	5077-67-8	$C_4H_8O_2$	806	-	-	[91]	Toasted	[109]
187	258	1.750	3-Hydroxy-2-pentanone	3142-66-3	$C_5H_{10}O_2$	819	807	[2]	[91]	Earthy, aged	[98]
188	264	1.870	2-Hydroxy-3-pentanone	5704-20-1	$C_5H_{10}O_2$	823	821	[49]	[91]	-	
189	294	0.930	3-Hexen-2-one	763-93-9	$C_6H_{10}O$	844	845	[50]		-	
190	294	1.010	3-Hexene-2,5-dione	4436-75-3	$C_6H_8O_2$	844	-	-		-	
191	300	0.650	4-Methyl-2-hexanone	105-42-0	$C_7H_{14}O$	848	848	[2]		-	
192	336	0.660	4-Heptanone	123-19-3	$C_7H_{14}O$	874	871	[4]	[91]	Ethereal-fruity, pineapple-like, strawberry-like	[114]
193	354	2.540	1-(Acetyloxy)-2-propanone	592-20-1	$C_5H_8O_3$	889	882	[28]	[91]	Fruity-buttery, sour	[114]
194	360	0.660	3-Heptanone	106-35-4	$C_7H_{14}O$	892	889	[4]	[91]	Green, fatty, fruity, sweet, ethereal	[114, 116]
195	366	0.720	2-Heptanone	110-43-0	$C_7H_{14}O$	896	895	[2]	[91]	Fruity, spicy, cinnamon, banana, spicy	[116]
196	444	0.940	3-Hepten-2-one	1119-44-4	$C_7H_{12}O$	942	942	[51]	[98]	Green-grassy	[116]
197	444	2.180	2,5-Hexanedione	110-13-4	$C_6H_{10}O_2$	943	949	[25]	[91]	Sweet-ethereal	[114]
198	468	0.660	6-Methyl-3-heptanone	624-42-0	$C_8H_{16}O$	956	941	[52]		-	
199	468	0.720	6-Methyl-2-heptanone	928-68-7	$C_8H_{16}O$	956	955	[53]		-	
200	486	0.740	5-Methyl-2-heptanone	18217-12-4	$C_8H_{16}O$	966	971	[54]		-	
201	510	0.820	1-Octen-3-one	4312-99-6	$C_8H_{14}O$	980	980	[4]	[91]	Mushroom	[116, 117, 122, 127]
202	522	0.850	2,3-Octanedione	585-25-1	$C_8H_{14}O_2$	987	985	[4]	[91]	Warmed-over	[116]
203	522	0.900	6-Methyl-5-hepten-2-one	110-93-0	$C_8H_{14}O$	987	985	[4]	[91]	Strong, fatty, green, citrus-like	[116]
204	534	0.740	2-Octanone	111-13-7	$C_8H_{16}O$	994	990	[4]	[91]	Floral, bitter-green, musty-herbaceous, unripe-apple fruity	[114, 116]
205	618	0.990	3-Octen-2-one	1669-44-9	$C_8H_{14}O$	1042	1040	[4]	[91]	Fruity, lemon	[116]
206	708	0.780	2-Nonanone	821-55-6	$C_9H_{18}O$	1093	1095	[4]	[91]	Fruity-floral, fatty, herbaceous, rue	[116, 130]
207	792	0.930	3-Nonen-2-one	14309-57-0	$C_9H_{16}O$	1143	1144	[55]		Fruity	[116]
			Cyclics								
208	294	1.780	2-Cyclopenten-1-one	930-30-3	C_5H_6O	845	835	[32]	[91]	-	
209	366	1.070	Cyclohexanone	108-94-1	$C_6H_{10}O$	896	895	[4]		Peppermint, acetone-like	[116]
210	366	4.250	4-Cyclopentene-1,3-dione	930-60-9	$C_5H_4O_2$	899	911	[31]	[109, 110]	-	
211	390	1.390	2-Methyl-2-cyclopenten-1-one	1120-73-6	C_6H_8O	911	920	[31]	[91]	-	
212	432	1.640	2-Cyclohexen-1-one	930-68-7	C_6H_8O	935	927	[56]	[91]	Gassy-mint	[114]
213	486	1.380	5-Ethylcyclopent-2-en-1-one	34094-63-8	$C_7H_{10}O$	966	966	[57]		-	
214	510	1.300	6-Methylenebicyclo[3.2.0]hept-3-en-2-one	-	C_8H_8O	980	-	-		-	
215	534	1.270	3-Methyl-2-cyclohexen-1-one	1193-18-6	$C_7H_{10}O$	994	-	-	[91]	Caramel-like, phenolic, mild cherry	[116, 130]
216	600	0.750	2,2,6-Trimethylcyclohexanone	2408-37-9	$C_9H_{16}O$	1032	1036	[5]		-	
217	600	3.800	2-Cyclohexene-1,4-dione	4505-38-8	$C_6H_6O_2$	1033	1036	[25]		-	
218	612	1.040	3,5-Dimethyl-2-cyclohexen-1-one	1123-09-7	$C_8H_{12}O$	1039	-	-		-	
219	612	5.470	2-Hydroxy-3-methyl-2-cyclopenten-1-one	80-71-7	$C_6H_8O_2$	1041	1042	[58]	[91]	Sweet, caramellic-spicy, walnut, maple, licorice, celery, tobacco	[114, 115, 124]
220	654	1.200	2,3,4-Trimethyl-2-cyclopenten-1-one	28790-86-5	$C_8H_{12}O$	1063	1071	[28]	[28]	-	
221	654	3.990	3,5-Dimethyl-1,2-cyclopentanedione	13494-07-0	$C_7H_{10}O_2$	1064	-	-	[91]	-	
222	708	2.120	3-Ethyl-2-hydroxy-2-cyclopenten-1-one	21835-01-8	$C_7H_{10}O_2$	1094	1091	[59]	[91]	Caramel-like, sweet, sugary	[116, 130]
			Aromatics								
223	660	2.190	Acetophenone	98-86-2	C_8H_8O	1067	1065	[2]	[91]	Sweet	[116]
224	768	2.140	1-Phenyl-2-propanone	103-79-7	$C_9H_{10}O$	1130	1131	[2]		-	
225	822	2.320	*o*-Hydroxyacetophenone	118-93-4	$C_8H_8O_2$	1162	1167	[60]	[91]	Sweet, heavy-floral, herbaceous, new-mown hay-like, mimosa -like	[114]
226	834	2.340	1-Phenyl-1,2-propanedione	579-07-7	$C_9H_8O_2$	1169	1175	[61]	[91]	Warm-floral, herbaceous, plastic	[114, 116]
227	864	1.950	*p*-Methylacetophenone	122-00-9	$C_9H_{10}O$	1187	1190	[2]		Fruity, floral	[116]
228	936	1.710	1-Phenyl-2-butanone	1007-32-5	$C_{10}H_{12}O$	1230	1231	[2]		-	

229	990	1.860	1-(4-Hydroxyphenyl)-1-propanone	70-70-2	$C_9H_{10}O_2$	1262	-	-		-	
230	1068	2.470	4-Hydroxy-3-methylacetophenone	876-02-8	$C_9H_{10}O_2$	1310	1311	[33]	[111]	-	
			**Volatile phenols**								
231	708	4.560	2-methoxyphenol (Guaiacol)	90-05-1	$C_7H_8O_2$	1096	1090	[27]	[91]	Smoke-like, phenolic, burnt, spicy, woody, meaty, sweet	[109, 115, 116, 118, 121, 130]
232	738	4.980	2,6-Dimethylphenol	576-26-1	$C_8H_{10}O$	1114	1108	[48]	[91]	Ground-coffee, phenolic	[125, 130]
233	876	1.540	2-Allylphenol	1745-81-9	$C_9H_{10}O$	1194	1170	[62]		-	
234	1020	3.090	4-Ethyl-2-methoxyphenol (4-Ethylguaiacol)	2785-89-9	$C_9H_{12}O_2$	1280	1290	[3]	[91]	Smoky, clove-like, spicy, burnt, vanilla-like, sweet, ethereal, green	[109, 116, 118, 121, 130]
235	1080	4.670	2-Methoxy-4-vinylphenol (4-Vinylguaiacol)	7786-61-0	$C_9H_{10}O_2$	1319	1322	[3]	[91]	Spicy, clove-like, phenolic, apple, rum, roasted peanut	[109, 114, 116, 118, 121, 130]
			**Oxazoles**								
236	120	0.750	4-Methyloxazole	693-93-6	C_4H_5NO	628	-	-		-	
237	198	0.890	4,5-Dimethyloxazole	20662-83-3	C_5H_7NO	747	750	[63]	[91]	-	
238	312	0.850	Trimethyloxazole	20662-84-4	C_6H_9NO	857	852	[63]	[91]	-	
239	426	0.780	4-Ethyl-2,5-dimethyloxazole	30408-61-8	$C_7H_{11}NO$	931	919	[39]	[91]	-	
240	570	0.730	2-Ethyl-4-methyl-5-propyloxazole	102586-53-8	$C_9H_{15}NO$	1014	-	-		-	
241	588	0.760	4,5-Dimethyl-2-propyloxazole	53833-32-2	$C_8H_{13}NO$	1025	-	-	[91]	-	
242	660	0.710	4,5-Dimethyl-2-isobutyloxazole	26131-91-9	$C_9H_{15}NO$	1066	-	-		-	
243	696	3.000	Benzoxazole	273-53-0	C_7H_5NO	1088	-	-	[91]	-	
244	744	1.830	2-Methylbenzoxazole	95-21-6	C_8H_7NO	1115	-	-	[91]	Sweet, gassy-pungent, floral-sweet, tobacco	[114, 130]
			**Pyrazines**								
245	180	1.230	Pyrazine	290-37-9	$C_4H_4N_2$	720	729	[28]	[91]	Pungent, sweet, floral, coffee	[109, 114, 129]
246	270	1.250	Methylpyrazine	109-08-0	$C_5H_6N_2$	827	833	[28]	[91]	Nutty, cocoa, green, roasted, chocolate, meaty, toasted	[109, 114, 129]
247	402	1.120	2,5-Dimethylpyrazine	123-32-0	$C_6H_8N_2$	918	912	[28]	[91]	Chocolate, roasted nuts, earthy, grassy, roasted, nutty	[116, 129, 130]
248	402	1.170	2-Ethylpyrazine	13925-00-3	$C_6H_8N_2$	918	922	[2]	[91]	Peanut butter, musty, nutty, woody, buttery, roasted, green, sweet	[116, 130]
249	414	1.190	2,3-Dimethylpyrazine	5910-89-4	$C_6H_8N_2$	925	922	[2]	[91]	Nutty, cocoa-like odor, green note, toasted, roasted	[109, 116, 129]
250	432	1.660	Vinylpyrazine	4177-16-6	$C_6H_6N_2$	935	935	[12]	[91]	-	
251	492	1.020	2-Isopropylpyrazine	9820-90-0	$C_7H_{10}N_2$	970	-	-		-	
252	540	1.040	2-Ethyl-6-methylpyrazine	13925-03-6	$C_7H_{10}N_2$	997	1000	[28]	[91]	Toasted, flowery, fruity, hazelnut-like	[109, 126, 129]
253	552	1.080	2-Ethyl-3-methylpyrazine	15707-23-0	$C_7H_{10}N_2$	1004	1001	[28]	[91]	Raw-potato, roasted, earthy, nutty, peanut-like, coffee-like	[109, 116, 129]
254	564	1.100	2-Propylpyrazine	18138-03-9	$C_7H_{10}N_2$	1011	1010	[63]	[91]	Green, vegetable, herbal	[116, 126]
255	576	1.410	2-Vinyl-6-methylpyrazine	13925-09-2	$C_7H_8N_2$	1018	1020	[12]	[91]	Coffee	[109]
256	588	2.470	Acetylpyrazine	22047-25-2	$C_6H_6N_2O$	1026	1023	[28]	[91]	Toasted	[109]
257	636	0.920	2-Methyl-3-isopropylpyrazine	15986-81-9	$C_8H_{12}N_2$	1052	1053	[2]		-	
258	654	1.000	Isobutylpyrazine	29460-92-2	$C_8H_{12}N_2$	1063	-	-		-	
259	672	1.710	Isopropenylpyrazine	38713-41-6	$C_7H_8N_2$	1073	1075	[63]	[91]	-	
260	684	0.940	2,6-Diethylpyrazine	13067-27-1	$C_8H_{12}N_2$	1080	1080	[12]	[91]	Toasted, potato-like, roasted	[109, 126, 129]
261	714	0.880	2-Isopropyl-3-methoxypyrazine	25773-40-4	$C_8H_{12}N_2O$	1097	1094	[64]	[91]	Vegetable-like, earthy, bell pepper, raw potato, galbanum, roasty	[116, 117, 118, 130]
262	732	1.670	6,7-Dihydro-5*H*-cyclopentapyrazine	23747-47-9	$C_7H_8N_2$	1108	1104	[65]	[91]	Green, phenolic, nutty, roast	[115, 130]
263	756	1.970	2-Acetyl-3-methylpyrazine	23787-80-6	$C_7H_8N_2O$	1123	1128	[28]	[91]	Cereal, roasted bean, roasted, nutty, grain-roasted potato	[116, 130]
264	780	0.930	2-Isobutyl-3-methylpyrazine	13925-06-9	$C_9H_{14}N_2$	1136	1134	[28]	[91]	Herbaceous green earthy notes, green bell peppers	[116]
265	786	1.390	5*H*-5-Methyl-6,7-dihydrocyclopentapyrazine	23747-48-0	$C_8H_{10}N_2$	1140	1149	[28]	[91]	Earthy, baked potato, peanut, roasted, nutty	[115, 116, 130]
266	792	1.350	2-Methyl-6-(1-propenyl)pyrazine (isomer)	104638-11-1	$C_8H_{10}N_2$	1144	-		[91]	-	
267	816	0.830	2,3-Diethyl-5-methylpyrazine	18138-04-0	$C_9H_{14}N_2$	1158	1153	[12]	[91]	Nutty, meaty, roasted hazelnut, earthy, roasty	[115, 116, 121, 122, 124]
268	822	1.450	2-Methyl-6-(1-propenyl)pyrazine (isomer)	104638-11-1	$C_8H_{10}N_2$	1162	-	-	[91]	-	
269	834	0.860	2-Acetyl-3-ethylpyrazine	32974-92-8	$C_8H_{10}N_2O$	1168	1172	[28]	[28]	-	
270	852	1.010	2-Isoamylpyrazine	40790-22-5	$C_9H_{14}N_2$	1179	-	-		-	
271	858	0.880	2-Isobutyl-3-methoxypyrazine	24683-00-9	$C_9H_{14}N_2O$	1183	1181	[64]	[91]	Green bell-pepper note, galbanum oil, red pepper, green, earthy	[116, 121, 128, 130]
272	870	0.940	2-Butyl-3-methylpyrazine	15987-00-5	$C_9H_{14}N_2$	1190	-	-	[91]	-	
273	894	0.830	2,5-Dimethyl-3-isobutylpyrazine	32736-94-0	$C_{10}H_{16}N_2$	1204	1207	[2]	[91]	-	
274	894	1.950	1-Methylpyrrolo(1,2-a)pyrazine	64608-59-9	$C_8H_8N_2$	1205	1214	[28]	[91]	-	
275	924	1.160	6,7-Dihydro-2,5-dimethyl-5*H*-cyclopentapyrazine	38917-61-2	$C_9H_{12}N_2$	1222	1220	[31]	[91]	-	
276	942	0.770	2,5-Diethyl-3,6-dimethylpyrazine	18903-30-5	$C_{10}H_{16}N_2$	1233	1225	[66]	[91]	-	
277	972	0.910	2-Methyl-6-isopentylpyrazine	91010-41-2	$C_{10}H_{16}N_2$	1251	1257	[28]		-	
278	1062	0.780	2,6-Dimethyl-3(2-methyl-1-butyl)pyrazine	56617-70-0	$C_{11}H_{18}N_2$	1305	1318	[28]		-	
279	1080	0.800	2,5-Dimethyl-3-isoamylpyrazine	18433-98-2	$C_{11}H_{18}N_2$	1317	1319	[67]		-	
280	1092	0.850	2,3-Dimethyl-5-isopentylpyrazine	18450-01-6	$C_{11}H_{18}N_2$	1325	1332	[68]		-	
			**Pyridines**								
281	186	1.090	Pyridine	110-86-1	C_5H_5N	729	726	[28]	[91]	Pungent, nauseating, warm, burnt, smoky, coffee-like	[109, 114, 116, 129]
282	270	1.020	2-Methylpyridine	109-06-8	C_6H_7N	827	824	[63]	[91]	Roasted popcorn, coffee	[109, 130]
283	360	0.950	2,6-Dimethylpyridine	108-48-5	C_7H_9N	892	887	[5]	[91]	Minty-tarry, pyridine, peppermint	[116]
284	390	0.980	2-Ethylpyridine	100-71-0	C_7H_9N	911	919	[31]	[91]	-	
285	480	1.200	3-Ethylpyridine	536-78-7	C_7H_9N	963	963	[28]	[91]	Tobacco, caramel, burnt, coffee-like, toasted	[109, 116, 129, 130]
286	498	1.700	3-Vinylpyridine	1121-55-7	C_7H_7N	973	972	[5]		-	
287	600	2.130	2-Acetylpyridine	1122-62-9	C_7H_7NO	1032	1045	[28]	[91]	Popcorn, bready, tobacco, cracker-like, roasted barley	[116, 130]

288	786	2.450	Methyl 3-pyridinecarboxylate	93-60-7	$C_7H_7NO_2$	1141	1137	[5]	[91]	Nauseating, sweet-herbaceous, mildly tobacco-like, fresh, caramel	[114, 116]
289	888	0.920	2-Pentylpyridine	2294-76-0	$C_{10}H_{15}N$	1201	1205	[69]		Tallowy-like	[116]
			**Pyrroles**								
290	180	0.910	1-Methylpyrrole	96-54-8	C_5H_7N	720	731	[28]	[91]	Smokey-tarry, sweet, woody-herbaceous, animal, coffee	[109, 114]
291	204	5.870	Pyrrole	109-97-7	C_4H_5N	763	768	[28]	[91]	Warm, slightly pungent, hay-like herbaceous, sweet, green, toasted	[109, 114, 116, 129]
292	252	0.920	1-Ethylpyrrole	617-92-5	C_6H_9N	814	818	[63]	[91]	-	
293	330	1.100	2,5-Dimethylpyrrole	625-84-3	C_6H_9N	870	867	[28]	[91]	-	
294	426	0.970	2-Ethyl-4-methylpyrrole	69687-77-0	$C_7H_{11}N$	932	938	[70]	[98]	-	
295	474	2.390	1-Acetylpyrrole	609-41-6	C_6H_7NO	960	957	[71]	[91]	-	
296	528	0.920	3-Ethyl-2,4-dimethylpyrrole	517-22-6	$C_8H_{13}N$	990	988	[28]	[91]	-	
297	552	2.590	1-Methyl-2-formylpyrrole	1192-58-1	C_6H_7NO	1005	1010	[5]	[91]	Cracker-popcorn, burnt	[129, 130]
298	630	2.020	1-Ethyl-2-formylpyrrole	2167-14-8	C_7H_9NO	1049	1067	[70]	[91]	-	
299	672	2.070	2-Acetyl-1-methylpyrrole	932-16-1	C_7H_9NO	1074	1096	[31]	[91]	-	
300	672	2.850	2-Acetylpyrrole	1072-83-9	C_6H_7NO	1074	1098	[28]	[91]	Bread, walnut, licorice, cracker, popcorn-like	[109, 116, 130]
			**Sulfur compounds**								
301	180	0.710	Dimethyl disulfide	624-92-0	$C_2H_6S_2$	719	719	[3]	[91]	Onion	[114, 116]
302	294	1.550	Methylthio-2-propanone	14109-72-9	C_4H_8OS	845	863	[72]		Melon	[116]
303	354	1.440	2-Furylmethylsulfide	13129-38-9	C_5H_6OS	888	894	[73]	[91]	Garlic-like	[130]
304	390	1.870	2-Furfurylthiol	98-02-2	C_5H_6OS	911	918	[63]	[91]	Coffee-like, caramellic-burnt, sweet, roasty, sulfury	[114, 118, 121, 128, 129]
305	390	2.060	3-(Methylthio)propanal (Methional)	3268-49-3	C_4H_8OS	912	912	[4]	[91]	Onion, meat-like, bouillon-like, soup-like, cooked potato-like	[114, 116, 117, 123, 128]
306	444	1.420	1-(Methylthio)-2-butanone	13678-58-5	$C_5H_{10}OS$	942	-	-	[91]	Mushroom, garlic	[116, 130]
307	486	1.190	Dimethyl trisulfide	3658-80-8	$C_2H_6S_3$	966	969	[4]	[91]	Fresh onion, cabbage-like, brothy, sulfury, pungent	[98, 114, 116, 128]
308	510	3.580	3-Mercapto-3-methyl-1-butanol	34300-94-2	$C_5H_{12}OS$	981	-		[91]	Sweet, soup-like, cooked meat, spicy, smoke-roast, meaty, chicken	[98, 124, 130]
309	546	1.520	2-Furfuryl methyl sulfide	1438-91-1	C_6H_8OS	1001	995	[74]	[91]	Coffee-like, onion, garlic, burnt, sulfury, cooked cabbage	[98, 109, 116, 130]
310	582	1.540	3-Mercapto-3-methylbutyl formate	50746-10-6	$C_6H_{12}O_2S$	1022	1020	[75]	[91]	Sweaty, fruity, black currant-like, catty, orange flowers, roasty	[118, 121, 124, 128, 130]
311	732	1.230	3-Mercapto-3-methylbutyl acetate	50746-09-3	$C_7H_{14}O_2S$	1108	-	[76]	[112]	-	
312	906	1.950	Furfuryl methyl disulfide	57500-00-2	$C_6H_8OS_2$	1212	1226	[29]	[91]	Fresh white bread crust	[130]
			**Terpenic compounds**								
			Monoterpenes								
313	426	0.440	α-Pinene	80-56-8	$C_{10}H_{16}$	931	932	[3]	[99]	Pine, turpentine-like	[116]
314	498	0.490	β-Pinene	127-91-3	$C_{10}H_{16}$	973	982	[77]	[99]	Turpentine, dry, woody, resinous	[116]
315	522	0.470	2,6-Dimethyl-2,6-octadiene (isomer)	2792-39-4	$C_{10}H_{18}$	986	990	[78]		-	
316	528	0.540	β-Myrcene	123-35-3	$C_{10}H_{16}$	990	988	[1]	[91]	Sweet, balsamic, plastic, sweet-balsamic-resinous gum	[114, 116]
317	552	0.470	2,6-Dimethyl-2,6-octadiene (isomer)	2792-39-4	$C_{10}H_{18}$	1004	990	[78]		-	
318	552	0.560	α-Phellandrene	99-83-2	$C_{10}H_{16}$	1004	1007	[77]	[113]	Fresh, citrus, peppery, discrete mint, minty, herbaceous	[116]
319	570	0.560	α-Terpinene	99-86-5	$C_{10}H_{16}$	1014	1016	[2]	[99]	Woody, terpene, lemon	[116]
320	582	0.670	*p*-Cymene	99-87-6	$C_{10}H_{14}$	1021	1026	[79]	[91]	Carrot-like, kerosene-like	[114, 116]
321	594	0.550	Limonene	138-86-3	$C_{10}H_{16}$	1028	1031	[46]	[91]	Citrusy, lemon-like, fresh, sweet	[130]
322	612	0.590	β-Ocimene (isomer)	13877-91-3	$C_{10}H_{16}$	1038	1042	[77]	[97]	Warm herbaceous	[116]
323	630	0.610	β-Ocimene (isomer)	13877-91-3	$C_{10}H_{16}$	1049	1042	[77]	[97]	Warm herbaceous	[116]
324	648	0.600	γ-Terpinene	99-85-4	$C_{10}H_{16}$	1059	1062	[80]	[99]	Lemon	[116]
325	696	0.610	α-Terpinolene	586-62-9	$C_{10}H_{16}$	1087	1093	[77]		Sweet, pine	[116]
326	702	0.940	*p*-Cymenene	1195-32-0	$C_{10}H_{12}$	1090	1095	[81]	[91]	Citrusy-lemon-like, gassy	[114]
327	786	0.790	Cosmene	460-01-5	$C_{10}H_{14}$	1140	1134	[82]		-	
328	798	0.670	*Allo*-ocimene	673-84-7	$C_{10}H_{16}$	1147	1144	[80]		-	
			Monoterpenoids								
329	672	0.970	Linalool oxide (isomer)	1365-19-1	$C_{10}H_{18}O_2$	1073	1078	[83]	[91]	Sweet, woody, floral, woody-earthy undertone, pungent	[114, 116]
330	702	0.970	Linalool oxide (isomer)	1365-19-1	$C_{10}H_{18}O_2$	1090	1078	[83]	[91]	Sweet, woody, floral, woody-earthy undertone, pungent	[114, 116]
331	720	1.220	Linalool	78-70-6	$C_{10}H_{18}O$	1101	1101	[2]	[91]	Floral-woody, faintly citrusy note, floral, sweet-fruity	[114-116, 118, 124, 125]
332	876	1.430	α-Terpineol	98-55-5	$C_{10}H_{18}O$	1194	1206	[80]	[91]	Floral, lilac	[130]
333	888	1.200	Safranal	116-26-7	$C_{10}H_{14}O$	1201	1201	[5]		Saffron-like	[116]
334	918	1.050	*p*-Menth-1-en-9-al	29548-14-9	$C_{10}H_{16}O$	1219	1219	[80]		-	
			Sesquiterpenes								
335	1152	0.560	α-Cubebene	17699-14-8	$C_{15}H_{24}$	1364	1366	[84]		-	
336	1170	0.560	α-Copaene	3856-25-5	$C_{15}H_{24}$	1376	1375	[84]	[28]	-	
337	1212	0.610	Longifolene	475-20-7	$C_{15}H_{24}$	1405	1395	[84]		-	
338	1236	0.620	β-Caryophyllene	87-44-5	$C_{15}H_{24}$	1421	1417	[84]		Cloves, turpentine	[116]
339	1296	0.650	α-Humulene	6753-98-6	$C_{15}H_{24}$	1455	1450	[84]		-	
340	1356	0.660	α-Muurolene	31983-22-9	$C_{15}H_{24}$	1505	1490	[84]		-	
341	1386	0.660	δ-Cadinene	483-76-1	$C_{15}H_{24}$	1525	1525	[84]	[28]	-	
			**Norisoprenoids**								
342	1020	0.710	Vitispirane (C_{13})	65416-59-3	$C_{13}H_{20}O$	1279	1289	[3]		-	
343	1098	0.850	Theaspirane (C_{13})	36431-72-8	$C_{13}H_{22}O$	1329	1315	[85]		Fruity, woody, sweetish	[116]

344	1134	0.980	1,2-Dihydro-1,1,6-trimethylnaphthalene (TDN) (C_{13})	30364-38-6	$C_{13}H_{16}$	1353	1363	[3]		-	
345	1182	1.070	β-Damascenone (C_{13})	23726-93-4	$C_{13}H_{18}O$	1385	1395	[3]	[91]	Tea-like, fruity, honey-like, fruity, sweet-fruity	[118, 121, 124-127, 130]
346	1248	1.000	α-Ionone (C_{13})	127-41-3	$C_{13}H_{20}O$	1430	1439	[11]		Warm, woody, berry characteristic violet-like	[116]
347	1290	0.960	Geranyl acetone (C_{13})	689-67-8	$C_{13}H_{22}O$	1459	1463	[3]		Green and rosy floral, fresh-floral, sweet-rosy, slightly green	[116]
			**Thiazoles**								
348	180	1.470	Thiazole	288-47-1	C_3H_3NS	720	730	[63]	[91]	Green, sweet, nutty, tomato, toasted	[109, 116]
349	252	1.150	2-Methylthiazole	3581-87-1	C_4H_5NS	814	815	[63]	[91]	-	
350	264	1.350	4-Methylthiazole	693-95-8	C_4H_5NS	823	823	[28]	[91]	Nutty, green, roasted	[116, 129]
351	294	1.330	5-Methylthiazole	3581-89-3	C_4H_5NS	844	846	[86]	[91]	-	
352	360	1.040	2,4-Dimethylthiazole	541-58-2	C_5H_7NS	892	892	[86]	[91]	Salty, sulfury, burnt, rubber	[98]
353	408	1.080	2,5-Dimethylthiazole	4175-66-0	C_5H_7NS	921	934	[72]	[91]	-	
354	432	1.270	4,5-Dimethylthiazole	3581-91-7	C_5H_7NS	935	947	[63]	[91]	Roasted nutty, boiled poultry	[116]
355	462	1.320	5-Ethylthiazole	17626-73-2	C_5H_7NS	952	955	[63]	[91]	-	
356	498	0.970	2-Ethyl-4-methylthiazole	15679-12-6	C_6H_9NS	973	-	-	[91]	Nutty, green	[116]
357	540	1.220	4-Propylthiazole	41981-60-6	C_6H_9NS	997	-	-		-	
358	564	1.030	5-Ethyl-2-methylthiazole	19961-52-5	C_6H_9NS	1011	1015	[86]	[91]	Rubber-like	[126]
359	576	0.870	2-Isopropyl-4-methylthiazole	15679-13-7	$C_7H_{11}NS$	1018	-	-		Green, vegetable, nutty, rooty, earthy	[116]
360	576	1.150	5-Ethyl-4-methylthiazole	31883-01-9	C_6H_9NS	1018	1039	[86]	[91]	Nutty, green, earthy	[130]
361	576	2.690	2-Acetylthiazole	24295-03-2	C_5H_5NOS	1019	1036	[63]	[91]	Green onion, herbal, grassy	[116]
362	660	0.880	4-Ethyl-2,5-dimethylthiazole	32272-57-4	$C_7H_{11}NS$	1066	1066	[86]	[91]	-	
363	678	0.940	5-Ethyl-2,4-dimethylthiazole	38205-61-7	$C_7H_{11}NS$	1076	1078	[86]	[91]	Earthy, roasty	[118]
364	738	1.990	2-Acetyl-4-methylthiazole	7533-07-5	C_6H_7NOS	1112	1128	[70]	[91]	-	
365	756	2.220	2-Propanoyl-thiazole	43039-98-1	C_6H_7NOS	1123	1119	[39]		-	
366	930	2.900	Benzothiazole	95-16-9	C_7H_5NS	1227	1231	[2]	[91]	Delicate, persistent, rose-like	[116]
			**Tiophene compounds**								
367	126	0.650	Thiophene	110-02-1	C_4H_4S	637	626	[49]	[91]	-	
368	204	0.730	2-Methylthiophene	554-14-3	C_5H_6S	756	771	[63]	[91]	Onion, sulfury	[130]
369	216	0.770	3-Methylthiophene	616-44-4	C_5H_6S	774	783	[63]	[91]	-	
370	324	0.780	2-Ethylthiophene	872-55-9	C_6H_8S	866	872	[63]	[95, 97]	-	
371	330	0.740	2,5-Dimethylthiophene	638-02-8	C_6H_8S	870	877	[63]	[98]	-	
372	342	0.800	2,4-Dimethylthiophene	638-00-6	C_6H_8S	879	882	[63]		-	
373	354	0.840	2,3-Dimethylthiophene	632-16-6	C_6H_8S	888	898	[63]	[98]	-	
374	378	1.300	2-Vinylthiophene	1918-82-7	C_6H_6S	904	915	[63]	[98]	-	
375	414	1.660	3-Methoxythiophene	17573-92-1	C_5H_6OS	925	-	-		-	
376	456	2.670	3-Thiophanone	1003-04-9	C_4H_6OS	950	942	[87]	[91]	Garlic meaty, green vegetable, buttery	[116]
377	492	0.790	2-Isopropylthiophene	4095-22-1	$C_7H_{10}S$	969	976	[88]		-	
378	510	0.780	2,3,4-Trimethylthiophene	1795-04-6	$C_7H_{10}S$	980	993	[89]		-	
379	522	1.890	Dihydro-2-methyl-3(2H)-thiophenone	13679-85-1	C_5H_8OS	987	995	[63]	[91]	-	
380	528	3.660	3-Thiophenecarboxaldehyde	498-62-4	C_5H_4OS	992	1003	[49]		-	
381	540	2.880	Dihydro-2(3H)-thiophenone	1003-10-7	C_4H_6OS	998	-	-	[91]	-	
382	546	3.670	2-Thiophenecarboxaldehyde	98-03-3	C_5H_4OS	1002	1004	[90]	[91]	Coffee	[109]
383	690	2.550	3-Methyl-2-thiophenecarboxaldehyde	5834-16-2	C_6H_6OS	1084	1105	[66]	[91]	-	
384	690	3.220	3-Acetylthiophene	1468-83-3	C_6H_6OS	1085	-	-	[91]	-	
385	702	3.180	2-Acetylthiophene	88-15-3	C_6H_6OS	1092	1112	[63]	[91]	-	
386	708	0.830	2,5-Diethylthiophene	5069-23-8	$C_8H_{12}S$	1094	-	-		-	
387	738	2.260	Methyl-2-thiophene carboxylate	5380-42-7	$C_6H_6O_2S$	1112	-	-	[91]	-	
388	750	2.680	5-Methyl-2-thiophenecarboxaldehyde	13679-70-4	C_6H_6OS	1120	1124	[39]	[91]	-	
389	822	0.770	2-Pentylthiophene	4861-58-9	$C_9H_{14}S$	1161	1165	[63]		-	
390	864	2.390	2-Propionylthiophene	13679-75-9	C_7H_8OS	1187	1181	[90]	[91]	-	

[a] Retention times for first (^1t_R) and second (^2t_R) dimension in seconds.

[b] RI_{Calc}: Retention Index obtained through the modulated chromatogram.

[c] RI_{Lit}: Retention Index reported in the literature for 5% phenyl polysilphenylene-siloxane GC column or equivalents [1-90].

[d] Compound already identified in roasted coffee beans and/or brews [28, 91-113].

[e] Aroma descriptors [98, 109, 114-130].

References

1. Martins, C., et al., *Insights on beer volatile profile: Optimization of solid-phase microextraction procedure taking advantage of the comprehensive two-dimensional gas chromatography structured separation.* Journal of Separation Science, 2015. **38**(12): p. 2140-2148.
2. Alves, Z., et al., *Exploring the <italic>Saccharomyces cerevisiae</italic> Volatile Metabolome: Indigenous versus Commercial Strains.* Plos One, 2015. **10**(11): p. e0143641.
3. Santos, M.C., et al., *High pressure treatments accelerate changes in volatile composition of sulphur dioxide-free wine during bottle storage.* Food Chemistry, 2015. **188**: p. 406-414.
4. Costa, C.P., et al., *Shedding light on Aspergillus niger volatile exometabolome.* Scientific Reports, 2016. **6**: p. 27441.
5. Leffingwell, J.C. and E.D. Alford, *Volatile constituents of Perique tobacco.* Electron. J. Environ. Agric. Food Chem., 2005. **4**(2): p. 899-915.
6. Bellesia, F., et al., *Volatile Compounds of the White Truffle (Tuber magnatum Pico) from Middle Italy.* Flavour and Fragrance Journal, 1996. **11**(4): p. 239-243.
7. Mebazaa, R., et al., *Characterisation of volatile compounds in Tunisian fenugreek seeds.* Food Chemistry, 2009. **115**(4): p. 1326-1336.
8. Rocha, S.M., et al., *Exploring the potentialities of comprehensive two-dimensional gas chromatography coupled to time of flight mass spectrometry to distinguish bivalve species: Comparison of two clam species (Venerupis decussata and Venerupis philippinarum).* Journal of Chromatography A, 2013. **1315**: p. 152-161.
9. Miyazaki, T., et al., *Distribution of aroma volatile compounds in tangerine hybrids and proposed inheritance.* Journal of the Science of Food and Agriculture, 2011. **91**(3): p. 449-460.
10. Romeo, V., et al., *Headspace Volatiles of Vicia sativa L. (Leguminoseae) by Solid-Phase Microextraction and Gas Chromatography/Mass Spectrometry.* Journal of Essential Oil Research, 2009. **21**(1): p. 33-35.
11. Silva, I., et al., *Headspace solid-phase microextraction combined with comprehensive two-dimensional gas chromatography time-of-flight mass spectrometry for the determination of volatile compounds from marine salt.* Journal of Chromatography A, 2010. **1217**(34): p. 5511-5521.
12. van Loon, W.A.M., et al., *Identification and olfactometry of French fries flavour extracted at mouth conditions.* Food Chemistry, 2005. **90**(3): p. 417-425.
13. Bauchot, A.D., et al., *Effect of Aminocyclopropane-1-carboxylic Acid Oxidase Antisense Gene on the Formation of Volatile Esters in Cantaloupe Charentais Melon (Cv. Védrandais).* Journal of Agricultural and Food Chemistry, 1998. **46**(11): p. 4787-4792.
14. Maia, J.G.S., E.H.A. Andrade, and M.d.G.B. Zoghbi, *Volatile Constituents of the Leaves, Fruits and Flowers of Cashew (Anacardium occidentale L.).* Journal of Food Composition and Analysis, 2000. **13**(3): p. 227-232.
15. Scheidig, C., M. Czerny, and P. Schieberle, *Changes in key odorants of raw coffee beans during storage under defined conditions.* Journal of Agricultural and Food Chemistry, 2007. **55**(14): p. 5768-5775.
16. Figuérédo, G., et al., *Studies of Mediterranean oregano populations—V. Chemical composition of essential oils of oregano: Origanum syriacum L. var. bevanii (Holmes) Ietswaart, O. syriacum L. var. sinaicum (Boiss.) Ietswaart, and O. syriacum L. var. syriacum from Lebanon and Israel.* Flavour and Fragrance Journal, 2005. **20**(2): p. 164-168.
17. Sampaio, T.S. and P.C.L. Nogueira, *Volatile components of mangaba fruit (Hancornia speciosa Gomes) at three stages of maturity.* Food Chemistry, 2006. **95**(4): p. 606-610.
18. Tret'yakov, K.V., *Retention Data. NIST Mass Spectrometry Data Center.* 2007.
19. Costa, R., et al., *An investigation on the volatile composition of some Artemisia species from Iran.* Flavour and Fragrance Journal, 2009. **24**(2): p. 75-82.
20. Zhao, J., et al., *Chemical composition of the volatiles of three wild Bergenia species from western China.* Flavour and Fragrance Journal, 2006. **21**(3): p. 431-434.
21. Isidorov, V.A., et al., *Partition coefficients of alkyl aromatic hydrocarbons and esters in a hexane–acetonitrile system.* Journal of Chromatography A, 2001. **923**(1–2): p. 127-136.
22. Cao, H., Z. Li, and X. Chen, *QSRR Study of GC Retention Indices of Volatile Compounds Emitted from Mosla chinensis Maxim by Multiple Linear Regression.* Chinese Journal of Chemistry, 2011. **29**(10): p. 2187-2196.
23. Rodríguez-Burruezo, A., et al., *Analysis of the Volatile Aroma Constituents of Parental and Hybrid Clones of Pepino (Solanum muricatum).* Journal of Agricultural and Food Chemistry, 2004. **52**(18): p. 5663-5669.
24. Spadone, J.C., G. Takeoka, and R. Liardon, *Analytical Investigation of Rio Off-Flavor in Green Coffee.* Journal of Agricultural and Food Chemistry, 1990. **38**(1): p. 226-233.
25. Ferreira, L.R., et al., *Aqueous phase reactions of pentoses in the presence of nanocrystalline zeolite beta: Identification of by-products and kinetic modelling.* Chemical Engineering Journal, 2013. **215–216**: p. 772-783.
26. Antunes, M.M., et al., *Catalytic dehydration of d-xylose to 2-furfuraldehyde in the presence of Zr-(W,Al) mixed oxides. Tracing by-products using two-dimensional gas chromatography-time-of-flight mass spectrometry.* Catalysis Today, 2012. **195**(1): p. 127-135.
27. Perestrelo, R., et al., *In-Depth Search Focused on Furans, Lactones, Volatile Phenols, and Acetals As Potential Age Markers of Madeira Wines by Comprehensive Two-Dimensional Gas Chromatography with Time-of-Flight Mass Spectrometry Combined with Solid Phase Microextraction.* Journal of Agricultural and Food Chemistry, 2011. **59**(7): p. 3186-3204.
28. Risticevic, S., E. Carasek, and J. Pawliszyn, *Headspace solid-phase microextraction–gas chromatographic–time-of-flight mass spectrometric methodology for geographical origin verification of coffee.* Analytica Chimica Acta, 2008. **617**(1–2): p. 72-84.

29. Elmore, J.S., et al., *Effect of the Polyunsaturated Fatty Acid Composition of Beef Muscle on the Profile of Aroma Volatiles.* Journal of Agricultural and Food Chemistry, 1999. **47**(4): p. 1619-1625.
30. Solina, M., et al., *Volatile aroma components of soy protein isolate and acid-hydrolysed vegetable protein.* Food Chemistry, 2005. **90**(4): p. 861-873.
31. Ames, J.M., R.C.E. Guy, and G.J. Kipping, *Effect of pH, Temperature, and Moisture on the Formation of Volatile Compounds in Glycine/Glucose Model Systems.* Journal of Agricultural and Food Chemistry, 2001. **49**(9): p. 4315-4323.
32. Xu, X., et al., *Comprehensive two-dimensional gas chromatography (GC x GC) measurements of volatile organic compounds in the atmosphere.* Atmospheric Chemistry and Physics, 2003. **3**: p. 665-682.
33. Neves, P., et al., *Production of biomass-derived furanic ethers and levulinate esters using heterogeneous acid catalysts.* Green Chemistry, 2013. **15**(12): p. 3367-3376.
34. Zhao, Y., et al., *EXtraction, preparation and identification of volatile compounds in Changyu XO brandy.* **Chinese J. Chromatogr.**, 2008. **26**(2): p. 212-222.
35. Whitfield, F. and D. Mottram, *Investigation of the reaction between 4-hydroxy-5-methyl-3(2H)-furanone and cysteine or hydrogen sulfide at pH 4.5.* Journal of Agricultural and Food Chemistry, 1999. **47**(4): p. 1626-1634.
36. Pino, J., et al., *Volatile components from Mango (Mangifera indica L.) cultivars.* Journal of Agricultural and Food Chemistry, 2005. **53**(6): p. 2213-2223.
37. Kim, M., et al., *Determination of volatile flavor components in danggui cultivars by solvent free injection and hydrodistillation followed by gas chromatographic-mass spectrometric analysis.* Journal of Chromatography a, 2006. **1116**(1-2): p. 259-264.
38. Majcher, M., P. Lawrowski, and H. Jelen, *Comparison of original and adulterated oscypek cheese based on volatile and sensory profiles.* Acta Sci. Pol. Technol. Aliment., 2010. **9**(3): p. 265-275.
39. Whitfield, F. and D. Mottram, *Heterocyclic volatiles formed by heating cysteine or hydrogen sulfide with 4-hydroxy-5-methyl-3 (2H)-furanone at pH 6.5.* Journal of Agricultural and Food Chemistry, 2001. **49**(2): p. 816-822.
40. Fan, W. and M. Qian, *Characterization of aroma compounds of Chinese "Wuliangye" and "Jiannanchun" liquors by aroma extract dilution analysis.* Journal of Agricultural and Food Chemistry, 2006. **54**(7): p. 2695-2704.
41. Macku, C. and T. Shibamoto, *Volatile sulfur-containing compounds generated from the thermal interaction of corn oil and cysteine.* Journal of Agricultural and Food Chemistry, 1991. **39**(11): p. 1987-1989.
42. Kallio, M., et al., *Comprehensive two-dimensional gas chromatography coupled to time-of-flight mass spectrometry in the identification of organic compounds in atmospheric aerosols from coniferous forest.* Journal of Chromatography a, 2006. **1125**(2): p. 234-243.
43. Kotowska, U., M. Zalikowski, and V. Isidorov, *HS-SPME/GC-MS analysis of volatile and semi-volatile organic compounds emitted from municipal sewage sludge.* Environmental Monitoring and Assessment, 2012. **184**(5): p. 2893-2907.
44. Wang, Z., M. Fingas, and K. Li, *Fractionation of a light crude-oil and identification and quantitation of aliphatic, aromatic, and biomarker compounds by GC-FID and GC-MS - Part II.* Journal of Chromatographic Science, 1994. **32**(9): p. 367-382.
45. Mondello, L., *HS-SPME-GCxGC-MS analysis of Yerba Mate (Ilex paraguariensis) in Shimadzu GC-GC application compendium of comprehensive 2D GC.* Shimadzu Corp., 2012. **1**: p. 1-29.
46. Salvador, Â.C., et al., *Can Volatile Organic Metabolites Be Used to Simultaneously Assess Microbial and Mite Contamination Level in Cereal Grains and Coffee Beans?* Plos One, 2013. **8**(4): p. e59338.
47. Hashizume, M., M. Gordon, and D. Mottram, *Light-induced off-flavor development in cloudy apple juice.* Journal of Agricultural and Food Chemistry, 2007. **55**(22): p. 9177-9182.
48. Rostad, C.E. and W.E. Pereira, *Kovats and lee retention indices determined by gas chromatography/mass spectrometry for organic compounds of environmental interest.* Journal of High Resolution Chromatography, 1986. **9**(6): p. 328-334.
49. Elmore, J.S., et al., *The effects of diet and breed on the volatile compounds of cooked lamb.* Meat Science, 2000. **55**(2): p. 149-159.
50. Timon, M., et al., *Volatile compounds in supercritical carbon dioxide extracts of Iberian ham.* Journal of Agricultural and Food Chemistry, 1998. **46**(12): p. 5143-5150.
51. Pino, J., E. Sauri-Dauh, and R. Marbot, *Changes in volatile compounds of Habanero chile pepper (Capsicum chinense Jack. cv. Habanero) at two ripening stages.* Food Chemistry, 2006. **94**(3): p. 394-398.
52. Usai, M., et al., *Composition and variability of the essential oil of Sardinian Thymus herba-barona Loisel.* Flavour and Fragrance Journal, 2003. **18**(1): p. 21-25.
53. Vasta, V., J. Ratel, and E. Engel, *Mass spectrometry analysis of volatile compounds in raw meat for the authentication of the feeding background of farm animals*. Journal of Agricultural and Food Chemistry, 2007. **55**(12): p. 4630-4639.
54. Owens, J., et al., *Formation of volatile compounds during Bacillus subtilis fermentation of soya beans.* Journal of the Science of Food and Agriculture, 1997. **74**(1): p. 132-140.

55. Elmore, J.S., et al., *Effect of Lipid Composition on Meat-like Model Systems Containing Cysteine, Ribose, and Polyunsaturated Fatty Acids.* Journal of Agricultural and Food Chemistry, 2002. **50**(5): p. 1126-1132.
56. Kim, J. and H. Chung, *GC-MS Analysis of the Volatile Components in Dried Boxthorn (Lycium chinensis) Fruit.* Journal of the Korean Society For Applied Biological Chemistry, 2009. **52**(5): p. 516-524.
57. Turchini, G.M., et al., *Discrimination of origin of farmed trout by means of biometrical parameters, fillet composition and flavor volatile compounds.* Italian Journal of Animal Science, 2004. **3**(2): p. 123-140.
58. Mateo, J., et al., *Volatile Compounds in Spanish Paprika.* Journal of Food Composition and Analysis, 1997. **10**(3): p. 225-232.
59. de Simon, B., et al., *Volatile Compounds in Acacia, Chestnut, Cherry, Ash, and Oak Woods, with a View to Their Use in Cooperage.* Journal of Agricultural and Food Chemistry, 2009. **57**(8): p. 3217-3227.
60. Radulovic, N.S., et al., *Volatiles from vegetative organs of the paleoendemic resurrection plants Ramonda serbica Panc. and Ramonda nathaliae Panc. at Petrov.* J. Serb. Chem. Soc., 2009. **74**: p. 35-44.
61. Wang, Q., et al., *Chemical variation in the essential oil of Ephedra sinica from Northeastern China.* Food Chemistry, 2006. **98**(1): p. 52-58.
62. Adams, R.P., *Systematics of smooth leaf margin Juniperus of the western hemisphere based on leaf essential oils and RAPD DNA fingerprinting.* Biochemical Systematics and Ecology, 2000. **28**(2): p. 149-162.
63. Ames, J., R. Guy, and G. Kipping, *Effect of pH and temperature on the formation of volatile compounds in cysteine/reducing sugar/starch mixtures during extrusion cooking.* Journal of Agricultural and Food Chemistry, 2001. **49**(4): p. 1885-1894.
64. Escudero, A., et al., *Analytical characterization of the aroma of five premium red wines. Insights into the role of odor families and the concept of fruitiness of wines.* Journal of Agricultural and Food Chemistry, 2007. **55**(11): p. 4501-4510.
65. Boylston, T.D. and B.T. Viniyard, *Isolation of volatile flavor compounds from peanut butter using purge-and-trap technique*, in *Instrumental Methods in Food and Beverage Analysis*, D. Wetzel and G. Charalambous, Editors. 1998, Elsevier, B.V. p. 225-243.
66. Moon, S.-Y., M.A. Cliff, and E.C.Y. Li-Chan, *Odour-active components of simulated beef flavour analysed by solid phase microextraction and gas chromatography–mass spectrometry and –olfactometry.* Food Research International, 2006. **39**(3): p. 294-308.
67. Oruna-Concha, M., S. Duckham, and J. Ames, *Comparison of volatile compounds isolated from the skin and flesh of four potato cultivars after baking.* Journal of Agricultural and Food Chemistry, 2001. **49**(5): p. 2414-2421.
68. Welty, W., et al., *Effects of milk fat, cocoa butter, or selected fat replacers on flavor volatiles of chocolate ice cream.* Journal of Dairy Science, 2001. **84**(1): p. 21-30.
69. Macku, C. and T. Shibamoto, *Headspace volatile compounds formed from heated corn-oil and corn-oil with glycine.* Journal of Agricultural and Food Chemistry, 1991. **39**(7): p. 1265-1269.
70. Bredie, W., D. Mottram, and R. Guy, *Aroma volatiles generated during extrusion cooking of maize flour.* Journal of Agricultural and Food Chemistry, 1998. **46**(4): p. 1479-1487.
71. Begnaud, F., C. Peres, and J. Berdague, *Characterization of volatile effluents of livestock buildings by solid-phase microextraction.* International Journal of Environmental Analytical Chemistry, 2003. **83**(10): p. 837-849.
72. Bredie, W., D. Mottram, and R. Guy, *Effect of temperature and pH on the generation of flavor volatiles in extrusion cooking of wheat flour.* Journal of Agricultural and Food Chemistry, 2002. **50**(5): p. 1118-1125.
73. Shivashankar, S., T.K. Roy, and P.N.R. Moorthy, *Headspace solid phase micro extraction and GC/MS analysis of the volatile components in seed and cake of Azadirachta indica A. juss.* Chem. Bull. of Politechnika Univ. Timisoara Romania, 2012. **57**: p. 1-6.
74. Bastos, D., et al., *Essential oil and antioxidant activity of green mate and mate tea (Ilex paraguariensis) infusions.* Journal of Food Composition and Analysis, 2006. **19**(6-7): p. 538-543.
75. Sanz, C., et al., *Comparison of potent odorants in a filtered coffee brew and in an instant coffee beverage by aroma extract dilution analysis (AEDA).* European Food Research and Technology, 2002. **214**(4): p. 299-302.
76. Vermeulen, C. and S. Collin, *Combinatorial synthesis and sensorial properties of 21 mercapto esters.* Journal of Agricultural and Food Chemistry, 2003. **51**(12): p. 3618-3622.
77. Petronilho, S., et al., *Assessment of the terpenic profile of Callistemon citrinus (Curtis) Skeels from Mexico.* Industrial Crops and Products, 2013. **46**: p. 369-379.
78. Buchin, S., et al., *Identification de composés monoterpéniques, sesquiterpéniques et benzéniques dans un lait d'alpage très riche en ces substances.* Mitt. Lebensmittelunters. Hyg., 2002. **93**: p. 199-216.
79. Adams, R.P., et al., *DNA fingerprinting and terpenoid analysis of Juniperus blancoi var. huehuentensis (Cupressaceae), a new subalpine variety from Durango, Mexico.* Biochemical Systematics and Ecology, 2006. **34**(3): p. 205-211.

80. Jalali, H., et al., *Deeper insight into the monoterpenic composition of Ferula gummosa oleo-gum-resin from Iran.* Industrial Crops and Products, 2012. **36**(1): p. 500-507.
81. Zeng, Y., et al., *Comparative analysis of volatile components from Clematis species growing in China.* Analytica Chimica Acta, 2007. **595**(1-2): p. 328-339.
82. Flamini, G., P.L. Cioni, and I. Morelli, *Differences in the fragrances of pollen and different floral parts of male and female flowers of Laurus nobilis.* J. Agric. Food Chem., 2002. **50**: p. 4647-4652.
83. Liu, J., et al., *Volatile constituents of the leaves and flowers of Salvia przewalskii Maxim. from Tibet.* Flavour and Fragrance Journal, 2006. **21**(3): p. 435-438.
84. Jalali, H., et al., *Assessment of the sesquiterpenic profile of Ferula gummosa oleo-gum-resin (galbanum) from Iran. Contributes to its valuation as a potential source of sesquiterpenic compounds.* Industrial Crops and Products, 2013. **44**: p. 185-191.
85. Campeol, E., et al., *Volatile compounds from three cultivars of Olea europaea from Italy.* Journal of Agricultural and Food Chemistry, 2001. **49**(11): p. 5409-5411.
86. Mottram, D. and F. Whitfield, *Volatile compounds from the reaction of cysteine, ribose, and phospholipid in low-moisture systems.* Journal of Agricultural and Food Chemistry, 1995. **43**(4): p. 984-988.
87. Mahadevan, K. and L. Farmer, *Key Odor Impact Compounds in Three Yeast Extract Pastes.* Journal of Agricultural and Food Chemistry, 2006. **54**(19): p. 7242-7250.
88. Setkova, L., S. Risticevic, and J. Pawliszyn, *Rapid headspace solid-phase microextraction-gas chromatographic-time-of-flight mass spectrometric method for qualitative profiling of ice wine volatile fraction - II: Classification of Canadian and Czech ice wines using statistical evaluation of the data.* Journal of Chromatography a, 2007. **1147**(2): p. 224-240.
89. Damste, J., et al., *Identification of homologous series of alkylated thiophenes, thiolanes, thianes and benzothiophenes present in pyrolysates of sulfur-rich kerogens.* Journal of Chromatography, 1988. **435**(3): p. 435-452.
90. Madruga, M. and D. Mottram, *The effect of pH on the formation of volatile compounds produced by heating a model system containing 5 '-Imp and cysteine.* Journal of the Brazilian Chemical Society, 1998. **9**(3): p. 261-271.
91. Flament, I. and Y. Bessière-Thomas, *Coffee Flavor Chemistry*. 2002: Wiley.
92. Lindinger, C., et al., *Identification of Ethyl Formate as a Quality Marker of the Fermented Off-note in Coffee by a Nontargeted Chemometric Approach.* Journal of Agricultural and Food Chemistry, 2009. **57**(21): p. 9972-9978.
93. Maeztu, L., et al., *Characterization of espresso coffee aroma by static headspace GC-MS and sensory flavor profile.* Journal of Agricultural and Food Chemistry, 2001. **49**(11): p. 5437-5444.
94. Cordero, C., C. Bicchi, and P. Rubiolo, *Group-type and fingerprint analysis of roasted food matrices (coffee and hazelnut samples) by comprehensive two-dimensional gas chromatography.* Journal of Agricultural and Food Chemistry, 2008. **56**(17): p. 7655-7666.
95. Sanz, C., et al., *Optimizing headspace temperature and time sampling for identification of volatile compounds in ground roasted Arabica coffee.* Journal of Agricultural and Food Chemistry, 2001. **49**(3): p. 1364-1369.
96. Nebesny, E., et al., *The effect of roasting method on headspace composition of robusta coffee bean aroma.* European Food Research and Technology, 2007. **225**(1): p. 9-19.
97. Piccino, S., et al., *Aromatic composition and potent odorants of the "specialty coffee" brew "Bourbon Pointu" correlated to its three trade classifications.* Food Research International, 2014. **61**: p. 264-271.
98. Chin, S.T., G.T. Eyres, and P.J. Marriott, *Identification of potent odourants in wine and brewed coffee using gas chromatography-olfactometry and comprehensive two-dimensional gas chromatography.* Journal of Chromatography A, 2011. **1218**(42): p. 7487-7498.
99. Mondello, L., et al., *Reliable characterization of coffee bean aroma profiles by automated headspace solid phase microextraction-gas chromatography-mass spectrometry with the support of a dual-filter mass spectra library.* Journal of Separation Science, 2005. **28**(9-10): p. 1101-1109.
100. Lopez-Darias, J., et al., *Developing qualitative extraction profiles of coffee aromas utilizing polymeric ionic liquid sorbent coatings in headspace solid-phase microextraction gas chromatography-mass spectrometry.* Analytical and Bioanalytical Chemistry, 2011. **401**(9): p. 2965-2976.
101. Sanz, C., et al., *Profiles of volatile compounds and sensory analysis of three blends of coffee: influence of different proportions of Arabica and Robusta and influence of roasting coffee with sugar.* Journal of the Science of Food and Agriculture, 2002. **82**(8): p. 840-847.
102. Pereira, G., et al., *Conducting starter culture-controlled fermentations of coffee beans during on-farm wet processing: Growth, metabolic analyses and sensorial effects.* Food Research International, 2015. **75**: p. 348-356.
103. Evangelista, S., et al., *Improvement of coffee beverage quality by using selected yeasts strains during the fermentation in dry process.* Food Research International, 2014. **61**: p. 183-195.
104. Evangelista, S., et al., *Inoculation of starter cultures in a semi-dry coffee (Coffea arabica) fermentation process.* Food Microbiology, 2014. **44**: p. 87-95.
105. Wang, C., et al., *Coffee flavour modification through controlled fermentations of green coffee beans by Saccharomyces cerevisiae and Pichia kluyveri: Part I. Effects from individual yeasts.* Food Research International, 2020. **136**.

106. Akiyama, M., et al., *Analysis of volatile compounds released during the grinding of roasted coffee beans using solid-phase microextraction.* Journal of Agricultural and Food Chemistry, 2003. **51**(7): p. 1961-1969.
107. Akiyama, M., et al., *Characterization of headspace aroma compounds of freshly brewed arabica coffees and studies on a characteristic aroma compound of Ethiopian coffee.* Journal of Food Science, 2008. **73**(5): p. C335-C346.
108. Ascrizzi, R. and G. Flamini, *Wild Harenna coffee: flavour profiling from the bean to the cup.* European Food Research and Technology, 2020. **246**(3): p. 643-660.
109. Gonzalez-Rios, O., et al., *Impact of "ecological" post-harvest processing on coffee aroma: II. Roasted coffee.* Journal of Food Composition and Analysis, 2007. **20**(3-4): p. 297-307.
110. Cui, D., et al., *Application of SPME-GC-TOFMS, E-nose, and sensory evaluation to investigate the flavor characteristics of Chinese Yunnan coffee at three different conditions (beans, ground powder, and brewed coffee).* Flavour and Fragrance Journal, 2020. **35**(5): p. 541-560.
111. Lee, L., et al., *Modulation of the volatile and non-volatile profiles of coffee fermented with Yarrowia lipolytica: II. Roasted coffee.* Lwt-Food Science and Technology, 2017. **80**: p. 32-42.
112. Kumazawa, K. and H. Masuda, *Identification of odor-active 3-mercapto-3-methylbutyl acetate in volatile fraction of roasted coffee brew isolated by steam distillation under reduced pressure.* Journal of Agricultural and Food Chemistry, 2003. **51**(10): p. 3079-3082.
113. Farag, M., et al., *Sensory Metabolite Profiling in a Date Pit Based Coffee Substitute and in Response to Roasting as Analyzed via Mass Spectrometry Based Metabolomics.* Molecules, 2019. **24**(18).
114. Arctander, S., *Perfume and Flavor Chemicals: (aroma Chemicals)*. 1969: Allured Publishing Corporation.
115. Akiyama, M., et al., *Analysis of the Headspace Volatiles of Freshly Brewed Arabica Coffee Using Solid-Phase Microextraction.* Journal of Food Science, 2007. **72**(7): p. C388-C396.
116. Burdock, G.A., *Fenaroli's Handbook of Flavor Ingredients, Fifth Edition*. 2004, Boca Raton, FL: CRC Press.
117. Czerny, M. and W. Grosch, *Potent odorants of raw Arabica coffee. Their changes during roasting.* Journal of Agricultural and Food Chemistry, 2000. **48**(3): p. 868-872.
118. Blank, I., A. Sen, and W. Grosch, *Potent odorants of the roasted powder and brew of Arabica coffee.* Zeitschrift für Lebensmittel-Untersuchung und Forschung, 1992. **195**(3): p. 239-245.
119. Gonzalez-Rios, O., et al., *Impact of "ecological" post-harvest processing on the volatile fraction of coffee beans: I. Green coffee.* Journal of Food Composition and Analysis, 2007. **20**(3): p. 289-296.
120. Cantergiani, E., et al., *Characterisation of the aroma of green Mexican coffee and identification of mouldy/earthy defect.* European Food Research and Technology, 2001. **212**(6): p. 648-657.
121. Semmelroch, P. and W. Grosch, *Analysis of roasted coffee powders and brews by gas chromatography-olfactometry of headspace samples.* LWT - Food Science and Technology, 1995. **28**(3): p. 310-313.
122. Hofmann, T. and P. Schieberle, *Chemical Interactions between Odor-Active Thiols and Melanoidins Involved in the Aroma Staling of Coffee Beverages.* Journal of Agricultural and Food Chemistry, 2002. **50**(2): p. 319-326.
123. Holscher, W. and H. Steinhart, *Investigation of roasted coffee freshness with an improved headspace technique.* Zeitschrift für Lebensmittel-Untersuchung und Forschung, 1992. **195**(1): p. 33-38.
124. Akiyama, M., et al., *Analysis of Freshly Brewed Espresso Using a Retronasal Aroma Simulator and Influence of Milk Addition.* Food Science and Technology Research, 2009. **15**(3): p. 233-244.
125. Holscher, W. and H. Steinhart, *Formation Pathways for Primary Roasted Coffee Aroma Compounds*, in *Thermally Generated Flavors*. 1993, American Chemical Society. p. 206-217.
126. López-Galilea, I., et al., *Changes in Headspace Volatile Concentrations of Coffee Brews Caused by the Roasting Process and the Brewing Procedure.* Journal of Agricultural and Food Chemistry, 2006. **54**(22): p. 8560-8566.
127. Holscher, W. and H. Steinhart, *Aroma compounds in green coffee*, in *Developments in Food Science*, G. Charalambous, Editor. 1995, Elsevier. p. 785-803.
128. de Melo Pereira, G.V., et al., *Exploring the impacts of postharvest processing on the aroma formation of coffee beans – A review.* Food Chemistry, 2019. **272**: p. 441-452.
129. Zapata, J., et al., *Characterization of aroma compounds present in an industrial recovery concentrate of coffee flavour.* CyTA - Journal of Food, 2018. **16**(1): p. 367-372.
130. Flament, I., *Coffee Flavor Chemistry*. 2001, East Sussex: Wiley.
