# Supplementary material for: Insights on Single-Dose Espresso Coffee Capsules’ Volatile Profile: From Ground Powder Volatiles to Prediction of Espresso Brew Aroma Properties
Source: Foods. 2021 Oct 19;10(10):2508. doi: 10.3390/foods10102508 (PMC8535219; doi:10.3390/foods10102508)
Supplement: Supplementary file 1 [file foods-10-02508-s001.zip › Supplementary Material/Supplementary Material-Table S2.pdf]

Supplementary Table S2
(Foods)

Insights on single-dose espresso coffee capsules' volatile profile: from ground powder volatiles to prediction of espresso brew aroma properties

Guido R. Lopes [1,†], Sílvia Petronilho [1,2,*,†], Andreia S. Ferreira [1], Mariana Pinto [1], Claúdia P. Passos [1], Elisabete Coelho [1], Carla Rodrigues [3], Cláudia Figueira [4], Sílvia M. Rocha [1] and Manuel A. Coimbra [1]

[1] LAQV-REQUIMTE, Chemistry Department, Campus Universitário de Santiago, University of Aveiro, 3810-193 Aveiro, Portugal; guido@ua.pt (G.R.L.); silviapetronilho@ua.pt (S.P.); a39493@ua.pt (A.S.F.); a39045@ua.pt (M.P.); cpassos@ua.pt (C.P.P.); ecoelho@ua.pt (E.C.); smrocha@ua.pt (S.M.R.); mac@ua.pt (M.A.C.)
[2] Chemistry Research Centre-Vila Real, Department of Chemistry, University of Trás os-Montes and Alto Douro, Quinta de Prados, 5001-801 Vila Real, Portugal
[3] Delta Ventures, Av. Infante D. Henrique 151-A 1950-405 Lisboa, Portugal; carla.rodrigues@grupo-nabeiro.pt
[4] Diverge, Grupo Nabeiro Innovation Center, Alameda dos Oceanos 65, 1.1, 1990-208 Lisboa, Portugal; claudia.figueira@gruponabeiro.com

* Correspondence: silviapetronilho@ua.pt
† G.R.L. and S.P. contributed equally as first authors.

Table S2. GC × GC peak areas of 390 volatile compounds putatively identified in 8 roasted ground capsule-coffee powders and respective espresso brews, using HS-SPME/GC × GC-ToFMS.

		Powder								Brew							
**Peak no.**	**Compound**	**Dec**	**1**	**2**	**3**	**4**	**5**	**6**	**Sup**	**Dec**	**1**	**2**	**3**	**4**	**5**	**6**	**Sup**
	**Acids**	1.56×10^{9} (17.9)	1.37×10^{9} (23.2)	1.57×10^{9} (15.5)	1.92×10^{9} (8.6)	1.66×10^{9} (34.8)	1.56×10^{9} (5.0)	1.62×10^{9} (25.2)	1.30×10^{9} (54.8)	2.88×10^{8} (20.3)	2.55×10^{8} (27.3)	1.55×10^{8} (38.1)	5.30×10^{7} (30.8)	2.71×10^{8} (19.0)	2.87×10^{8} (2.0)	2.01×10^{8} (17.6)	1.07×10^{8} (63.1)
	Aliphatics																
1	Acetic acid	1.35×10^{9}	9.47×10^{8}	1.07×10^{9}	1.43×10^{9}	1.10×10^{9}	1.08×10^{9}	1.18×10^{9}	8.69×10^{8}	2.82×10^{8}	2.38×10^{8}	1.37×10^{8}	4.90×10^{7}	2.55×10^{8}	2.71×10^{8}	1.93×10^{8}	9.90×10^{7}
2	Propanoic acid	7.14×10^{7}	6.02×10^{7}	9.17×10^{7}	4.15×10^{7}	1.25×10^{8}	2.68×10^{7}	2.90×10^{7}	5.04×10^{7}	4.73×10^{5}	1.32×10^{5}	1.32×10^{5}	1.32×10^{5}	9.86×10^{5}	2.05×10^{6}	2.23×10^{6}	1.32×10^{5}
3	Butanoic acid	4.27×10^{6}	4.50×10^{6}	7.42×10^{6}	5.96×10^{6}	1.31×10^{7}	7.72×10^{6}	6.96×10^{6}	5.90×10^{6}	4.59×10^{4}	4.59×10^{4}	1.07×10^{5}	4.59×10^{4}	4.59×10^{4}	4.59×10^{4}	4.59×10^{4}	4.59×10^{4}
4	Isovaleric acid	1.25×10^{8}	3.60×10^{8}	4.11×10^{8}	4.40×10^{8}	4.19×10^{8}	4.43×10^{8}	3.98×10^{8}	3.76×10^{8}	5.34×10^{6}	1.71×10^{7}	1.80×10^{7}	3.81×10^{6}	1.56×10^{7}	1.38×10^{7}	6.02×10^{6}	7.71×10^{6}
	**Alcohols**	5.50×10^{7} (4.0)	1.06×10^{8} (7.7)	1.14×10^{8} (8.5)	1.16×10^{8} (18.9)	7.20×10^{7} (24.7)	1.11×10^{8} (7.9)	8.49×10^{7} (8.1)	7.32×10^{7} (19.7)	5.94×10^{7} (5.1)	1.30×10^{8} (11.3)	7.40×10^{7} (29.2)	3.90×10^{7} (3.3)	1.14×10^{8} (7.0)	9.14×10^{7} (14.7)	4.58×10^{7} (17.1)	3.95×10^{7} (12.5)
	Aliphatics																
5	2-Methyl-1-propanol	4.95×10^{5}	6.61×10^{5}	1.60×10^{6}	4.55×10^{5}	1.80×10^{5}	1.44×10^{6}	7.85×10^{5}	4.81×10^{5}	3.83×10^{5}	4.95×10^{5}	3.69×10^{5}	3.44×10^{5}	5.35×10^{5}	3.77×10^{5}	3.23×10^{5}	1.50×10^{5}
6	3-Buten-1-ol	1.60×10^{6}	1.30×10^{6}	1.65×10^{6}	8.81×10^{5}	1.60×10^{5}	$1.48\times^{6}$	1.25×10^{6}	1.17×10^{6}	n.d.	n.d.	n.d.	n.d.	n.d.	n.d.	n.d.	n.d.
7	3-Methyl-3-buten-1-ol	8.77×10^{6}	1.27×10^{7}	1.64×10^{7}	1.26×10^{7}	6.67×10^{6}	2.48×10^{7}	1.72×10^{7}	6.80×10^{6}	9.49×10^{6}	8.06×10^{6}	5.09×10^{6}	1.85×10^{6}	8.01×10^{6}	1.05×10^{7}	6.78×10^{6}	1.75×10^{6}
8	2-Methyl-1-butanol	2.10×10^{6}	1.49×10^{7}	2.46×10^{7}	1.71×10^{7}	2.34×10^{6}	1.95×10^{7}	8.09×10^{6}	3.28×10^{6}	4.24×10^{6}	7.99×10^{6}	6.15×10^{6}	3.47×10^{6}	1.00×10^{7}	1.09×10^{7}	6.64×10^{6}	2.85×10^{6}
9	1-Pentanol	3.33×10^{6}	2.40×10^{6}	2.91×10^{6}	2.17×10^{6}	2.99×10^{6}	5.22×10^{6}	4.63×10^{6}	2.01×10^{6}	1.40×10^{6}	2.43×10^{6}	1.36×10^{6}	6.25×10^{5}	5.11×10^{6}	5.17×10^{6}	2.90×10^{6}	6.72×10^{5}
10	3-Methyl-2-buten-1-ol	3.29×10^{6}	6.58×10^{6}	7.74×10^{6}	6.01×10^{6}	3.75×10^{6}	7.85×10^{6}	1.00×10^{7}	3.55×10^{6}	3.08×10^{6}	4.13×10^{6}	2.32×10^{6}	1.20×10^{6}	3.76×10^{6}	4.63×10^{6}	4.07×10^{6}	9.92×10^{5}
11	2-Hexanol	2.69×10^{5}	6.51×10^{5}	5.68×10^{5}	6.45×10^{5}	2.32×10^{5}	3.57×10^{5}	1.55×10^{5}	3.46×10^{5}	4.60×10^{5}	1.31×10^{6}	5.51×10^{5}	3.02×10^{5}	8.88×10^{5}	5.26×10^{5}	1.90×10^{5}	2.93×10^{5}
12	2-Heptanol	1.33×10^{7}	3.03×10^{7}	2.54×10^{7}	2.84×10^{7}	1.44×10^{7}	1.66×10^{7}	8.67×10^{6}	2.18×10^{7}	2.12×10^{7}	6.91×10^{7}	3.42×10^{7}	2.06×10^{7}	5.00×10^{7}	3.22×10^{7}	1.04×10^{7}	2.16×10^{7}
13	1-Octen-3-ol	1.12×10^{7}	1.61×10^{7}	8.38×10^{6}	1.47×10^{7}	1.27×10^{7}	1.10×10^{7}	1.49×10^{7}	5.65×10^{6}	1.27×10^{7}	2.35×10^{7}	1.58×10^{7}	7.29×10^{6}	2.66×10^{7}	1.72×10^{7}	1.11×10^{7}	7.92×10^{6}
14	2-Ethyl-1-hexanol	1.46×10^{6}	1.65×10^{6}	1.92×10^{6}	1.90×10^{6}	4.04×10^{6}	1.44×10^{6}	1.43×10^{6}	1.69×10^{6}	1.66×10^{6}	1.35×10^{6}	1.65×10^{6}	1.17×10^{6}	8.88×10^{5}	8.71×10^{5}	5.62×10^{5}	5.68×10^{5}
15	1-Octanol	6.00×10^{5}	8.53×10^{5}	6.04×10^{5}	1.15×10^{6}	1.10×10^{6}	1.07×10^{6}	7.09×10^{5}	1.12×10^{6}	1.23×10^{6}	2.32×10^{6}	1.38×10^{6}	8.71×10^{5}	1.67×10^{6}	2.32×10^{6}	1.19×10^{6}	5.97×10^{5}
	Aromatics																
16	2-Phenylethanol	8.60×10^{6}	1.74×10^{7}	2.23×10^{7}	3.05×10^{7}	2.34×10^{7}	2.03×10^{7}	1.71×10^{7}	2.53×10^{7}	3.57×10^{6}	9.46×10^{6}	5.10×10^{6}	1.31×10^{6}	6.42×10^{6}	6.75×10^{6}	1.63×10^{6}	2.12×10^{6}
	**Aldehydes**	4.31×10^{8} (7.7)	3.33×10^{8} (9.0)	3.40×10^{8} (16.1)	3.52×10^{8} (19.7)	2.78×10^{8} (26.3)	4.68×10^{8} (39.6)	3.65×10^{8} (9.1)	3.13×10^{8} (27.2)	1.08×10^{9} (3.1)	9.46×10^{8} (15.6)	6.25×10^{8} (22.1)	4.10×10^{8} (2.3)	8.59×10^{8} (8.4)	7.81×10^{8} (6.2)	5.36×10^{8} (16.9)	3.68×10^{8} (5.2)
	Aliphatics																
17	Acetaldehyde	1.81×10^{7}	1.49×10^{7}	1.46×10^{7}	2.50×10^{7}	1.54×10^{7}	1.99×10^{7}	1.18×10^{7}	1.05×10^{7}	1.30×10^{7}	1.14×10^{7}	3.01×10^{6}	6.85×10^{6}	1.23×10^{7}	1.23×10^{7}	8.83×10^{6}	5.40×10^{6}

18	2-Methylpropanal	1.31×10^{7}	1.41×10^{7}	1.44×10^{7}	1.39×10^{7}	5.04×10^{6}	2.03×10^{7}	1.41×10^{7}	7.68×10^{6}	1.27×10^{8}	5.35×10^{7}	4.95×10^{7}	4.11×10^{7}	4.34×10^{7}	6.20×10^{7}	4.48×10^{7}	2.52×10^{7}
19	2-Butenal	5.88×10^{6}	4.38×10^{6}	4.58×10^{6}	8.14×10^{6}	1.60×10^{6}	4.80×10^{6}	3.85×10^{6}	5.88×10^{6}	7.39×10^{6}	4.95×10^{6}	3.55×10^{6}	3.43×10^{6}	5.70×10^{6}	5.97×10^{6}	3.05×10^{6}	2.41×10^{6}
20	3-Methylbutanal	7.30×10^{7}	5.07×10^{7}	5.26×10^{7}	5.09×10^{7}	1.70×10^{7}	1.60×10^{8}	6.43×10^{7}	4.03×10^{7}	3.76×10^{8}	3.97×10^{8}	2.32×10^{8}	2.00×10^{8}	3.01×10^{8}	2.61×10^{8}	1.99×10^{8}	1.61×10^{8}
21	2-Methyl-2-butenal	8.70×10^{6}	1.12×10^{7}	1.40×10^{7}	7.70×10^{6}	3.04×10^{6}	1.46×10^{7}	9.45×10^{6}	4.84×10^{6}	3.01×10^{7}	2.84×10^{7}	1.52×10^{7}	9.34×10^{6}	2.63×10^{7}	2.92×10^{7}	1.65×10^{7}	7.06×10^{6}
22	2-Pentenal	2.22×10^{6}	1.00×10^{6}	1.19×10^{6}	2.10×10^{6}	5.44×10^{5}	1.14×10^{6}	1.14×10^{6}	1.08×10^{6}	4.10×10^{6}	3.21×10^{6}	1.29×10^{6}	1.38×10^{6}	3.53×10^{6}	3.23×10^{6}	2.15×10^{6}	1.31×10^{6}
23	Hexanal	7.67×10^{7}	3.81×10^{7}	3.89×10^{7}	3.37×10^{7}	3.02×10^{7}	4.97×10^{7}	5.29×10^{7}	4.83×10^{7}	1.24×10^{8}	8.84×10^{7}	6.44×10^{7}	3.21×10^{7}	1.19×10^{8}	8.93×10^{7}	6.28×10^{7}	4.84×10^{7}
24	4-Methyl-3-pentenal	5.42×10^{5}	5.09×10^{5}	5.73×10^{5}	4.36×10^{5}	1.53×10^{5}	5.58×10^{5}	3.54×10^{5}	3.18×10^{5}	3.60×10^{6}	3.06×10^{6}	1.51×10^{6}	1.09×10^{6}	3.10×10^{6}	3.18×10^{6}	1.52×10^{6}	8.67×10^{5}
25	2-Methyl-2-hexenal	6.28×10^{6}	7.24×10^{6}	7.80×10^{6}	5.18×10^{6}	7.57×10^{5}	5.77×10^{6}	3.95×10^{6}	4.40×10^{6}	1.14×10^{7}	1.76×10^{7}	9.93×10^{6}	6.39×10^{6}	1.23×10^{7}	1.40×10^{7}	6.82×10^{6}	5.42×10^{6}
26	2,4-Hexadienal	2.54×10^{6}	3.42×10^{6}	3.72×10^{6}	3.14×10^{6}	1.50×10^{6}	3.85×10^{6}	3.54×10^{6}	1.91×10^{6}	1.38×10^{6}	1.71×10^{6}	8.19×10^{5}	3.45×10^{5}	1.52×10^{6}	1.67×10^{6}	1.09×10^{6}	2.99×10^{5}
27	4-Methylhexanal	n.d.	n.d.	n.d.	n.d.	n.d.	n.d.	n.d.	n.d.	9.93×10^{5}	1.60×10^{6}	4.07×10^{5}	3.97×10^{5}	7.19×10^{5}	7.59×10^{5}	3.52×10^{5}	3.19×10^{5}
28	Heptanal	1.48×10^{7}	8.52×10^{6}	9.20×10^{6}	1.19×10^{7}	8.77×10^{6}	1.40×10^{7}	1.55×10^{7}	1.10×10^{7}	1.87×10^{7}	1.51×10^{7}	3.82×10^{7}	6.19×10^{6}	2.59×10^{7}	2.31×10^{7}	1.44×10^{7}	6.64×10^{6}
29	2-Heptenal	2.31×10^{7}	1.01×10^{7}	1.20×10^{7}	1.40×10^{7}	1.34×10^{7}	1.21×10^{7}	1.81×10^{7}	1.64×10^{7}	1.58×10^{7}	1.32×10^{7}	1.66×10^{7}	3.67×10^{6}	2.65×10^{7}	2.12×10^{7}	1.39×10^{7}	7.79×10^{6}
30	Octanal	5.58×10^{6}	4.43×10^{6}	4.16×10^{6}	4.41×10^{6}	5.77×10^{6}	4.00×10^{6}	4.76×10^{6}	4.66×10^{6}	6.17×10^{6}	4.59×10^{6}	4.29×10^{6}	3.29×10^{6}	5.29×10^{6}	5.50×10^{6}	3.05×10^{6}	2.94×10^{6}
31	2,4-Heptadienal	2.53×10^{6}	1.74×10^{6}	1.98×10^{6}	2.68×10^{6}	2.10×10^{6}	1.95×10^{6}	3.95×10^{6}	1.51×10^{6}	3.36×10^{6}	4.76×10^{6}	2.62×10^{6}	9.42×10^{5}	4.09×10^{6}	4.10×10^{6}	2.74×10^{6}	1.06×10^{6}
32	2-Octenal	1.27×10^{7}	3.23×10^{6}	7.03×10^{6}	1.32×10^{7}	1.22×10^{7}	7.63×10^{6}	1.18×10^{7}	1.15×10^{7}	1.34×10^{7}	9.32×10^{6}	9.99×10^{6}	2.83×10^{6}	1.41×10^{7}	1.05×10^{7}	7.21×10^{6}	4.63×10^{6}
33	Nonanal	2.86×10^{7}	2.08×10^{7}	2.39×10^{7}	2.33×10^{7}	3.14×10^{7}	1.82×10^{7}	1.94×10^{7}	2.58×10^{7}	3.31×10^{7}	2.24×10^{7}	1.67×10^{7}	1.31×10^{7}	2.20×10^{7}	1.94×10^{7}	1.29×10^{7}	9.68×10^{6}
34	2-Nonenal	9.95×10^{6}	8.66×10^{6}	5.93×10^{6}	7.45×10^{6}	1.19×10^{7}	7.44×10^{6}	9.64×10^{6}	1.09×10^{7}	1.68×10^{7}	1.31×10^{7}	8.59×10^{6}	5.25×10^{6}	1.29×10^{7}	1.11×10^{7}	6.79×10^{6}	4.18×10^{6}
35	Decanal	3.59×10^{6}	7.33×10^{6}	9.81×10^{6}	7.93×10^{6}	7.16×10^{6}	7.37×10^{6}	4.67×10^{6}	4.25×10^{6}	9.25×10^{6}	1.05×10^{7}	7.38×10^{6}	7.78×10^{6}	8.89×10^{6}	7.78×10^{6}	4.62×10^{6}	3.40×10^{6}
36	Undecanal	1.02×10^{5}	2.41×10^{5}	2.07×10^{5}	6.60×10^{4}	2.22×10^{5}	4.96×10^{5}	1.92×10^{5}	2.81×10^{5}	1.71×10^{6}	2.27×10^{6}	1.12×10^{6}	7.00×10^{5}	1.48×10^{6}	1.29×10^{6}	5.20×10^{5}	7.06×10^{5}
37	Dodecanal	2.10×10^{5}	2.24×10^{5}	1.88×10^{5}	4.65×10^{4}	3.83×10^{5}	2.12×10^{5}	1.83×10^{5}	2.71×10^{5}	2.24×10^{6}	3.12×10^{6}	2.11×10^{6}	4.98×10^{5}	2.18×10^{6}	2.12×10^{6}	1.61×10^{6}	1.45×10^{6}
	Aromatics																
38	Benzaldehyde	9.15×10^{7}	8.80×10^{7}	7.98×10^{7}	7.90×10^{7}	7.87×10^{7}	8.58×10^{7}	7.86×10^{7}	7.51×10^{7}	1.37×10^{8}	1.30×10^{8}	7.90×10^{7}	3.60×10^{7}	1.09×10^{8}	1.01×10^{8}	6.52×10^{7}	4.04×10^{7}
39	Benzeneacetaldehyde	9.98×10^{6}	1.41×10^{7}	1.21×10^{7}	1.27×10^{7}	1.11×10^{7}	7.87×10^{6}	1.07×10^{7}	7.72×10^{6}	8.23×10^{7}	7.15×10^{7}	3.86×10^{7}	1.93×10^{7}	6.68×10^{7}	6.37×10^{7}	3.84×10^{7}	1.90×10^{7}
40	2-Hydroxybenzaldehyde	1.24×10^{6}	1.17×10^{6}	1.39×10^{6}	1.30×10^{6}	1.71×10^{6}	1.51×10^{6}	1.75×10^{6}	1.11×10^{6}	2.59×10^{6}	2.91×10^{6}	1.56×10^{6}	6.01×10^{5}	2.48×10^{6}	2.36×10^{6}	1.65×10^{6}	5.24×10^{5}
41	2-Methylbenzaldehyde	8.38×10^{6}	9.11×10^{6}	9.82×10^{6}	9.47×10^{6}	8.05×10^{6}	9.53×10^{6}	1.05×10^{7}	7.70×10^{6}	2.27×10^{7}	1.92×10^{7}	1.02×10^{7}	4.44×10^{6}	1.69×10^{7}	1.61×10^{7}	1.03×10^{7}	4.19×10^{6}
42	2-Phenyl-2-butenal	1.19×10^{7}	9.76×10^{6}	1.06×10^{7}	1.48×10^{7}	1.03×10^{7}	9.57×10^{6}	9.57×10^{6}	1.01×10^{7}	1.42×10^{7}	1.30×10^{7}	6.72×10^{6}	3.16×10^{6}	1.16×10^{7}	8.75×10^{6}	5.52×10^{6}	3.08×10^{6}
	**Esters**	1.16×10^{8} (5.1)	1.24×10^{8} (14.6)	1.36×10^{8} (5.9)	1.43×10^{8} (5.2)	9.47×10^{7} (32.2)	1.53×10^{8} (10.7)	1.13×10^{8} (24.6)	1.23×10^{8} (20.1)	1.90×10^{8} (12.1)	2.61×10^{8} (14.9)	1.64×10^{8} (19.7)	8.89×10^{7} (32.2)	3.50×10^{8} (6.1)	2.64×10^{8} (14.3)	1.60×10^{8} (23.5)	1.03×10^{8} (13.9)
	Aliphatics																

43	Methyl acetate	2.19×10^{7}	1.77×10^{7}	1.37×10^{7}	2.15×10^{7}	7.61×10^{6}	2.32×10^{7}	1.31×10^{7}	2.38×10^{7}	3.73×10^{7}	3.43×10^{7}	2.12×10^{7}	1.37×10^{7}	5.84×10^{7}	2.13×10^{7}	3.03×10^{7}	2.53×10^{7}
44	Methyl propenoate	n.d.	n.d.	n.d.	n.d.	n.d.	n.d.	n.d.	n.d.	4.00×10^{5}	3.56×10^{5}	1.31×10^{5}	1.16×10^{5}	1.30×10^{4}	1.30×10^{4}	1.30×10^{4}	1.40×10^{5}
45	Methyl propanoate	3.16×10^{5}	5.75×10^{5}	9.46×10^{5}	1.96×10^{6}	2.77×10^{5}	1.50×10^{6}	8.91×10^{5}	9.22×10^{5}	5.77×10^{6}	7.43×10^{6}	3.58×10^{6}	3.04×10^{6}	6.66×10^{6}	7.41×10^{6}	4.52×10^{6}	1.31×10^{6}
46	Methyl glycolate	2.02×10^{6}	3.04×10^{6}	3.01×10^{6}	2.72×10^{6}	3.07×10^{6}	3.23×10^{6}	3.36×10^{6}	1.86×10^{6}	4.80×10^{4}	3.89×10^{4}	1.16×10^{4}	1.06×10^{3}	5.69×10^{4}	4.25×10^{4}	1.49×10^{4}	1.06×10^{3}
47	Methyl 2-methylpropenoate	2.82×10^{6}	7.85×10^{5}	6.13×10^{5}	8.13×10^{5}	1.53×10^{6}	1.49×10^{6}	1.92×10^{6}	2.07×10^{6}	4.97×10^{6}	1.63×10^{6}	1.17×10^{6}	9.01×10^{5}	4.05×10^{6}	2.48×10^{6}	5.92×10^{6}	1.50×10^{6}
48	Methyl butanoate	3.80×10^{5}	4.35×10^{5}	8.77×10^{5}	2.90×10^{5}	1.25×10^{5}	4.31×10^{5}	3.73×10^{5}	1.69×10^{5}	3.07×10^{6}	1.99×10^{6}	9.03×10^{5}	5.45×10^{5}	2.23×10^{6}	1.58×10^{6}	8.98×10^{5}	5.69×10^{5}
49	Methyl 2-butenoate	1.55×10^{6}	3.43×10^{6}	3.21×10^{6}	1.79×10^{6}	6.70×10^{5}	4.33×10^{6}	2.07×10^{6}	1.23×10^{6}	3.92×10^{6}	6.46×10^{6}	2.34×10^{6}	1.72×10^{6}	5.95×10^{6}	4.54×10^{6}	2.92×10^{6}	1.73×10^{6}
50	Methyl 2-methylbutanoate	3.51×10^{5}	5.58×10^{5}	7.00×10^{5}	4.67×10^{5}	1.43×10^{5}	8.78×10^{5}	4.31×10^{5}	2.03×10^{5}	9.80×10^{5}	1.62×10^{6}	1.15×10^{6}	6.24×10^{5}	2.49×10^{6}	2.37×10^{6}	1.06×10^{6}	5.49×10^{5}
51	Methyl 3-methylbutanoate	1.22×10^{6}	6.21×10^{6}	6.94×10^{6}	3.40×10^{6}	2.88×10^{5}	6.32×10^{6}	3.27×10^{6}	1.40×10^{6}	9.57×10^{6}	2.31×10^{7}	1.00×10^{7}	9.11×10^{6}	2.42×10^{7}	1.59×10^{7}	8.86×10^{6}	5.09×10^{6}
52	3-Methylbutyl formate	2.02×10^{5}	9.75×10^{5}	1.57×10^{6}	7.73×10^{5}	4.24×10^{5}	1.67×10^{6}	9.95×10^{5}	1.28×10^{6}	1.26×10^{6}	3.02×10^{6}	2.39×10^{6}	1.77×10^{6}	4.11×10^{6}	4.88×10^{6}	2.79×10^{6}	1.03×10^{6}
53	Butyl acetate	3.62×10^{6}	2.22×10^{6}	6.96×10^{6}	3.79×10^{6}	1.16×10^{6}	3.24×10^{6}	1.45×10^{6}	1.74×10^{6}	7.40×10^{6}	2.45×10^{6}	7.81×10^{6}	4.60×10^{6}	4.01×10^{7}	3.07×10^{7}	2.92×10^{5}	2.44×10^{6}
54	Methyl pentanoate	9.80×10^{4}	1.36×10^{5}	1.89×10^{5}	9.70×10^{4}	5.06×10^{4}	1.18×10^{5}	1.86×10^{5}	7.61×10^{4}	3.40×10^{5}	4.62×10^{5}	2.95×10^{5}	1.10×10^{5}	5.53×10^{5}	4.83×10^{5}	2.36×10^{5}	1.44×10^{5}
55	Methyl 3-methyl-2-butenoate	1.30×10^{6}	3.99×10^{6}	3.66×10^{6}	3.37×10^{6}	8.27×10^{5}	2.32×10^{6}	1.56×10^{6}	2.05×10^{6}	6.39×10^{6}	1.47×10^{7}	6.08×10^{6}	4.00×10^{6}	1.04×10^{7}	7.13×10^{6}	3.50×10^{6}	3.74×10^{6}
56	Ethyl 3-methylbutanoate	1.95×10^{6}	1.98×10^{6}	1.61×10^{6}	1.20×10^{6}	6.26×10^{5}	6.91×10^{5}	7.15×10^{5}	8.83×10^{5}	2.66×10^{6}	3.31×10^{6}	1.97×10^{6}	1.19×10^{6}	4.77×10^{6}	1.22×10^{6}	8.78×10^{5}	1.12×10^{6}
57	Isoamyl acetate	9.06×10^{6}	2.87×10^{6}	3.98×10^{6}	3.22×10^{6}	1.41×10^{6}	4.17×10^{6}	3.20×10^{6}	2.06×10^{6}	1.28×10^{7}	4.77×10^{6}	3.51×10^{6}	2.88×10^{6}	7.42×10^{6}	8.18×10^{6}	3.88×10^{6}	1.76×10^{6}
58	Methyl 3-methylpentanoate	4.43×10^{5}	6.46×10^{5}	6.58×10^{5}	4.29×10^{5}	2.99×10^{5}	2.81×10^{5}	3.46×10^{5}	3.33×10^{5}	3.27×10^{5}	9.32×10^{5}	3.77×10^{5}	4.30×10^{5}	7.63×10^{5}	4.74×10^{5}	1.58×10^{5}	2.57×10^{5}
59	3-Methyl-3-butenyl acetate	4.18×10^{6}	3.45×10^{6}	3.92×10^{6}	3.08×10^{6}	1.47×10^{6}	5.27×10^{6}	3.89×10^{6}	2.00×10^{6}	7.42×10^{6}	8.04×10^{6}	5.51×10^{6}	3.15×10^{6}	1.02×10^{7}	1.19×10^{7}	7.22×10^{6}	1.79×10^{6}
60	Methyl 4-methylpentanoate	1.29×10^{6}	4.95×10^{5}	2.90×10^{5}	5.54×10^{5}	9.41×10^{4}	2.88×10^{5}	3.22×10^{5}	2.12×10^{5}	7.90×10^{5}	1.68×10^{6}	7.35×10^{5}	4.12×10^{5}	1.13×10^{6}	7.48×10^{5}	3.54×10^{5}	3.63×10^{5}
61	Pentyl acetate	2.07×10^{6}	6.14×10^{5}	6.61×10^{5}	4.72×10^{5}	5.03×10^{5}	1.54×10^{6}	1.26×10^{6}	3.32×10^{5}	2.77×10^{6}	1.22×10^{6}	9.34×10^{5}	6.23×10^{5}	3.00×10^{6}	3.07×10^{6}	1.41×10^{6}	4.63×10^{5}
62	Methyl hexanoate	5.99×10^{5}	9.92×10^{5}	1.01×10^{6}	1.13×10^{6}	1.60×10^{6}	1.54×10^{6}	1.08×10^{6}	6.42×10^{5}	7.93×10^{5}	1.59×10^{6}	1.29×10^{6}	5.60×10^{5}	3.23×10^{6}	2.92×10^{6}	1.21×10^{6}	4.37×10^{5}
63	Ethyl tiglate	1.18×10^{6}	3.33×10^{6}	2.64×10^{6}	3.83×10^{6}	8.69×10^{5}	7.88×10^{5}	2.71×10^{5}	3.17×10^{6}	1.36×10^{6}	6.67×10^{6}	2.83×10^{6}	2.49×10^{6}	6.02×10^{6}	1.15×10^{6}	4.21×10^{5}	2.35×10^{6}
64	3-Methyl-2-butenyl acetate	6.83×10^{6}	1.17×10^{7}	1.45×10^{7}	9.83×10^{6}	5.69×10^{6}	1.80×10^{7}	1.94×10^{7}	6.57×10^{6}	7.39×10^{6}	2.38×10^{7}	1.94×10^{7}	7.40×10^{6}	3.13×10^{7}	3.72×10^{7}	2.86×10^{7}	6.41×10^{6}
65	Hexyl formate	9.67×10^{4}	1.08×10^{6}	1.80×10^{6}	1.09×10^{6}	1.40×10^{6}	5.03×10^{6}	3.79×10^{6}	8.59×10^{5}	1.42×10^{5}	1.77×10^{6}	2.06×10^{6}	1.04×10^{6}	6.97×10^{6}	8.86×10^{6}	3.69×10^{6}	7.22×10^{5}
66	Ethylidene acetate	5.06×10^{6}	6.86×10^{6}	6.77×10^{6}	6.78×10^{6}	3.40×10^{6}	6.87×10^{6}	5.60×10^{6}	4.97×10^{6}	1.24×10^{6}	1.57×10^{6}	8.67×10^{5}	4.27×10^{5}	1.54×10^{6}	1.56×10^{6}	8.58×10^{5}	4.50×10^{5}
67	Methyl 4-methyl-2-oxopentanoate	1.58×10^{6}	2.46×10^{6}	2.28×10^{6}	2.13×10^{6}	1.29×10^{6}	2.40×10^{6}	1.88×10^{6}	2.05×10^{6}	1.78×10^{6}	3.71×10^{6}	2.09×10^{6}	5.29×10^{5}	2.55×10^{6}	3.14×10^{6}	1.68×10^{6}	8.10×10^{5}
68	Butyl 2-methyl-2-propenoate	1.77×10^{7}	4.61×10^{6}	9.71×10^{6}	1.31×10^{7}	1.74×10^{7}	1.76×10^{7}	6.74×10^{6}	1.22×10^{7}	8.87×10^{6}	2.83×10^{6}	4.64×10^{6}	4.06×10^{6}	1.97×10^{7}	1.38×10^{7}	6.54×10^{6}	4.64×10^{6}
69	Hexyl acetate	2.61×10^{6}	1.04×10^{6}	1.89×10^{6}	1.39×10^{6}	2.84×10^{6}	5.86×10^{6}	4.88×10^{6}	1.10×10^{6}	2.15×10^{6}	1.17×10^{6}	1.34×10^{6}	6.36×10^{5}	4.86×10^{6}	6.03×10^{6}	3.00×10^{6}	4.24×10^{5}
70	Isobutyl 3-methyl-2-butenoate	7.93×10^{5}	1.96×10^{6}	1.40×10^{6}	2.39×10^{6}	1.33×10^{6}	1.32×10^{6}	1.45×10^{6}	3.73×10^{6}	3.70×10^{5}	1.59×10^{6}	1.15×10^{6}	6.79×10^{5}	1.18×10^{6}	8.49×10^{5}	5.01×10^{5}	7.03×10^{5}
71	Methyl octanoate	7.63×10^{4}	5.20×10^{5}	2.13×10^{5}	1.83×10^{5}	1.00×10^{6}	2.12×10^{5}	3.27×10^{5}	1.84×10^{5}	2.19×10^{6}	4.73×10^{6}	4.82×10^{6}	1.66×10^{5}	2.77×10^{6}	2.86×10^{6}	2.56×10^{6}	2.19×10^{6}

72	3-Methylbutyl 3-methyl-2-butenoate	1.64×10^{6}	1.83×10^{6}	1.42×10^{6}	2.94×10^{6}	5.30×10^{5}	3.91×10^{5}	2.33×10^{5}	3.46×10^{6}	1.39×10^{6}	1.16×10^{6}	5.65×10^{5}	6.72×10^{5}	3.71×10^{5}	2.32×10^{5}	9.54×10^{4}	6.75×10^{5}
73	Methyl decanoate	3.28×10^{4}	6.26×10^{4}	4.38×10^{4}	6.57×10^{4}	1.02×10^{5}	1.03×10^{5}	5.99×10^{4}	5.01×10^{4}	3.49×10^{6}	6.70×10^{6}	3.67×10^{6}	5.02×10^{4}	5.32×10^{6}	4.81×10^{6}	3.45×10^{6}	3.20×10^{6}
74	Methyl dodecanoate	6.92×10^{4}	5.55×10^{4}	7.26×10^{4}	5.08×10^{4}	1.80×10^{5}	1.26×10^{5}	8.89×10^{4}	1.00×10^{5}	9.43×10^{6}	1.41×10^{7}	1.13×10^{7}	7.38×10^{4}	1.93×10^{7}	1.32×10^{7}	9.16×10^{6}	1.21×10^{7}
	Aromatics																
75	Phenyl acetate	1.54×10^{6}	1.35×10^{6}	1.30×10^{6}	1.61×10^{6}	1.64×10^{6}	1.62×10^{6}	1.36×10^{6}	1.46×10^{6}	2.24×10^{6}	3.02×10^{6}	1.13×10^{6}	5.93×10^{5}	2.50×10^{6}	2.11×10^{6}	1.16×10^{6}	4.29×10^{5}
76	Benzyl formate	2.35×10^{5}	3.46×10^{5}	3.81×10^{5}	4.89×10^{5}	4.25×10^{5}	4.60×10^{5}	3.99×10^{5}	3.79×10^{5}	5.66×10^{5}	1.17×10^{6}	6.81×10^{5}	1.28×10^{5}	1.05×10^{6}	9.76×10^{5}	6.15×10^{5}	2.31×10^{5}
77	Methyl benzoate	1.45×10^{6}	3.19×10^{6}	2.99×10^{6}	3.75×10^{6}	2.25×10^{6}	1.87×10^{6}	1.66×10^{6}	3.51×10^{6}	2.50×10^{6}	7.52×10^{6}	3.85×10^{6}	2.13×10^{6}	5.12×10^{6}	2.98×10^{6}	1.35×10^{6}	1.93×10^{6}
78	Methyl phenylacetate	6.31×10^{6}	7.64×10^{6}	6.89×10^{6}	8.35×10^{6}	6.37×10^{6}	6.09×10^{6}	5.80×10^{6}	7.54×10^{6}	7.91×10^{6}	1.41×10^{7}	5.47×10^{6}	2.72×10^{6}	9.80×10^{6}	5.86×10^{6}	3.03×10^{6}	3.12×10^{6}
79	2-Phenylethyl formate	1.25×10^{6}	3.21×10^{6}	4.20×10^{6}	3.93×10^{6}	3.56×10^{6}	5.24×10^{6}	3.88×10^{6}	4.07×10^{6}	3.18×10^{6}	6.81×10^{6}	3.39×10^{6}	1.86×10^{6}	6.28×10^{6}	6.62×10^{6}	3.68×10^{6}	1.84×10^{6}
80	Methyl 2-hydroxybenzoate	9.92×10^{6}	1.53×10^{7}	1.71×10^{7}	2.18×10^{7}	1.75×10^{7}	1.38×10^{7}	1.29×10^{7}	1.72×10^{7}	2.01×10^{7}	3.04×10^{7}	1.82×10^{7}	1.04×10^{7}	2.72×10^{7}	2.02×10^{7}	1.18×10^{7}	7.75×10^{6}
81	2-Phenylethyl acetate	1.14×10^{6}	2.76×10^{6}	2.88×10^{6}	2.70×10^{6}	2.22×10^{6}	1.95×10^{6}	1.67×10^{6}	2.58×10^{6}	2.21×10^{6}	3.69×10^{6}	2.09×10^{6}	1.34×10^{6}	3.16×10^{6}	2.62×10^{6}	1.25×10^{6}	1.42×10^{6}
82	Ethyl 2-hydroxybenzoate	1.09×10^{6}	3.26×10^{6}	3.35×10^{6}	5.69×10^{6}	2.62×10^{6}	1.30×10^{6}	5.16×10^{5}	4.38×10^{6}	2.14×10^{6}	5.71×10^{6}	3.17×10^{6}	2.09×10^{6}	3.46×10^{6}	1.29×10^{6}	5.46×10^{5}	1.88×10^{6}
	**Furan compounds**	6.12×10^{9} (10.1)	5.22×10^{9} (15.5)	7.91×10^{9} (17.2)	6.77×10^{9} (23.7)	6.21×10^{9} (14.8)	8.04×10^{9} (15.8)	6.88×10^{9} (4.7)	5.40×10^{9} (28.6)	7.42×10^{9} (6.3)	7.20×10^{9} (12.7)	4.86×10^{9} (21.9)	3.31×10^{9} (7.1)	6.66×10^{9} (9.6)	6.58×10^{9} (8.6)	5.18×10^{9} (9.2)	2.97×10^{9} (10.2)
83	Furan	3.95×10^{6}	2.57×10^{6}	3.86×10^{6}	4.50×10^{6}	1.62×10^{6}	4.59×10^{6}	3.19×10^{6}	1.93×10^{6}	3.79×10^{7}	2.98×10^{7}	2.70×10^{7}	3.64×10^{7}	3.43×10^{7}	3.03×10^{7}	2.09×10^{7}	1.81×10^{7}
84	2-Methylfuran	5.41×10^{7}	4.11×10^{7}	6.06×10^{7}	3.50×10^{7}	1.65×10^{7}	6.70×10^{7}	4.59×10^{7}	2.69×10^{7}	2.31×10^{8}	1.81×10^{8}	1.91×10^{8}	1.94×10^{8}	2.52×10^{8}	2.01×10^{8}	1.72×10^{8}	1.12×10^{8}
85	Tetrahydrofuran	9.99×10^{7}	6.51×10^{7}	1.11×10^{8}	6.06×10^{7}	8.21×10^{6}	1.70×10^{8}	1.02×10^{8}	8.27×10^{7}	5.52×10^{7}	3.95×10^{7}	2.24×10^{7}	2.38×10^{7}	6.08×10^{7}	2.89×10^{7}	5.34×10^{7}	1.41×10^{7}
86	2,5-Dimethylfuran	2.13×10^{7}	3.52×10^{7}	4.51×10^{7}	1.24×10^{7}	3.55×10^{6}	5.15×10^{7}	2.39×10^{7}	7.63×10^{6}	1.29×10^{8}	8.76×10^{7}	5.10×10^{7}	4.11×10^{7}	8.69×10^{7}	7.92×10^{7}	4.02×10^{7}	1.91×10^{7}
87	2,4-Dimethylfuran	1.39×10^{6}	2.49×10^{6}	2.93×10^{6}	8.94×10^{5}	2.35×10^{5}	2.81×10^{6}	1.36×10^{6}	4.18×10^{5}	7.68×10^{6}	3.74×10^{6}	4.71×10^{6}	3.47×10^{6}	8.13×10^{6}	8.18×10^{6}	2.52×10^{6}	1.43×10^{6}
88	2-Propylfuran	7.49×10^{5}	9.27×10^{5}	1.06×10^{6}	5.16×10^{5}	3.11×10^{5}	1.31×10^{6}	7.04×10^{5}	2.35×10^{5}	8.00×10^{5}	6.94×10^{5}	4.60×10^{5}	3.54×10^{5}	1.22×10^{6}	1.15×10^{6}	4.02×10^{5}	1.45×10^{5}
89	2-Ethyl-5-methylfuran	2.62×10^{6}	6.93×10^{6}	5.41×10^{6}	2.72×10^{6}	1.42×10^{6}	1.26×10^{6}	3.86×10^{6}	1.25×10^{6}	4.70×10^{6}	6.49×10^{6}	3.36×10^{6}	2.43×10^{6}	6.62×10^{6}	6.56×10^{6}	2.64×10^{6}	9.09×10^{5}
90	2-Ethyl-5-methyltetrahydrofuran	1.20×10^{5}	5.84×10^{5}	6.43×10^{5}	4.13×10^{5}	7.85×10^{5}	5.08×10^{5}	8.91×10^{4}	1.91×10^{5}	6.75×10^{5}	1.71×10^{6}	1.11×10^{6}	1.01×10^{6}	3.26×10^{6}	1.56×10^{6}	3.03×10^{5}	7.85×10^{5}
91	2-Furancarbonitrile	1.87×10^{6}	2.04×10^{6}	1.96×10^{6}	1.88×10^{6}	3.77×10^{5}	1.43×10^{6}	1.48×10^{6}	1.50×10^{6}	4.72×10^{6}	3.88×10^{6}	1.65×10^{6}	8.43×10^{5}	2.69×10^{6}	2.63×10^{6}	1.45×10^{6}	9.15×10^{5}
92	Dihydro-2-methyl-3-furanone	1.62×10^{8}	2.02×10^{8}	2.13×10^{8}	2.75×10^{8}	1.24×10^{8}	1.96×10^{8}	2.19×10^{8}	1.58×10^{8}	9.92×10^{7}	1.11×10^{8}	7.01×10^{7}	3.91×10^{7}	1.15×10^{8}	1.12×10^{8}	1.02×10^{8}	3.28×10^{7}
93	2,3,5-Trimethylfuran	4.14×10^{6}	9.81×10^{6}	1.07×10^{7}	6.07×10^{6}	2.47×10^{6}	1.12×10^{7}	8.27×10^{6}	1.37×10^{6}	7.02×10^{6}	1.03×10^{7}	6.13×10^{6}	4.24×10^{6}	1.03×10^{7}	1.07×10^{7}	4.18×10^{6}	1.67×10^{6}
94	3-Furaldehyde	2.23×10^{6}	2.18×10^{6}	2.39×10^{6}	2.17×10^{6}	1.89×10^{6}	2.27×10^{6}	2.92×10^{6}	1.73×10^{6}	1.65×10^{6}	1.61×10^{6}	7.54×10^{5}	2.20×10^{5}	1.54×10^{6}	1.57×10^{6}	9.34×10^{5}	1.90×10^{5}
95	2-Vinyl-5-methylfuran	4.83×10^{7}	6.99×10^{7}	7.60×10^{7}	4.83×10^{7}	3.05×10^{6}	8.14×10^{7}	6.10×10^{7}	2.68×10^{7}	5.74×10^{7}	8.46×10^{7}	5.75×10^{7}	4.46×10^{7}	9.83×10^{7}	8.48×10^{7}	4.52×10^{7}	1.98×10^{7}
96	2-(Methoxymethyl)furan	3.72×10^{7}	6.15×10^{7}	6.23×10^{7}	5.27×10^{7}	2.25×10^{7}	7.18×10^{7}	5.07×10^{7}	3.06×10^{7}	6.25×10^{7}	8.54×10^{7}	5.54×10^{7}	3.31×10^{7}	9.41×10^{7}	8.41×10^{7}	6.12×10^{7}	2.75×10^{7}
97	2,3,4-Trimethylfuran	1.45×10^{6}	6.08×10^{6}	3.17×10^{6}	2.52×10^{6}	4.61×10^{5}	5.61×10^{6}	2.20×10^{6}	4.04×10^{5}	2.38×10^{6}	3.14×10^{6}	1.60×10^{6}	1.28×10^{6}	2.79×10^{6}	2.14×10^{6}	1.33×10^{6}	5.97×10^{5}
98	Furfural	6.51×10^{8}	6.05×10^{8}	5.87×10^{8}	6.20×10^{8}	7.08×10^{8}	6.11×10^{8}	6.88×10^{8}	4.70×10^{8}	8.75×10^{8}	8.00×10^{8}	5.88×10^{8}	3.65×10^{8}	7.19×10^{8}	7.11×10^{8}	6.46×10^{8}	3.85×10^{8}

99	2-(2-Propenyl)furan	4.33×10^{7}	6.91×10^{7}	6.20×10^{7}	4.65×10^{7}	1.66×10^{7}	7.31×10^{7}	5.04×10^{7}	2.50×10^{7}	4.01×10^{7}	5.57×10^{7}	3.36×10^{7}	2.42×10^{7}	6.37×10^{7}	6.05×10^{7}	3.14×10^{7}	1.37×10^{7}
100	5-Methyl-2(3H)-furanone	n.d.	n.d.	n.d.	n.d.	n.d.	n.d.	n.d.	n.d.	7.69×10^{6}	9.05×10^{6}	3.50×10^{6}	1.96×10^{6}	5.19×10^{6}	1.05×10^{7}	3.88×10^{6}	1.12×10^{6}
101	2-Furanmethanol	2.31×10^{9}	1.06×10^{9}	3.10×10^{9}	2.03×10^{9}	2.10×10^{9}	3.20×10^{9}	1.97×10^{9}	2.08×10^{9}	1.92×10^{9}	1.63×10^{9}	1.06×10^{9}	7.34×10^{8}	1.14×10^{9}	1.43×10^{9}	9.13×10^{8}	5.24×10^{8}
102	2,5-Diethyltetrahydrofuran	2.29×10^{5}	4.34×10^{5}	3.65×10^{5}	3.34×10^{5}	2.73×10^{4}	3.71×10^{5}	2.07×10^{5}	1.07×10^{5}	2.68×10^{5}	7.56×10^{5}	4.42×10^{5}	3.31×10^{5}	1.29×10^{6}	1.05×10^{6}	4.85×10^{5}	8.06×10^{4}
103	2-Butylfuran	1.96×10^{6}	1.22×10^{6}	1.53×10^{6}	9.00×10^{5}	1.03×10^{6}	2.73×10^{6}	1.87×10^{6}	8.26×10^{5}	4.15×10^{5}	4.16×10^{5}	5.04×10^{5}	1.72×10^{5}	1.50×10^{6}	1.32×10^{6}	4.69×10^{5}	1.46×10^{5}
104	Furfuryl formate	1.19×10^{8}	1.04×10^{8}	1.23×10^{8}	1.51×10^{8}	1.05×10^{8}	1.46×10^{8}	1.49×10^{8}	8.11×10^{7}	1.52×10^{8}	1.91×10^{8}	9.32×10^{7}	7.20×10^{7}	1.87×10^{8}	1.78×10^{8}	1.45×10^{8}	8.22×10^{7}
105	2-Acetylfuran	1.35×10^{8}	2.64×10^{8}	2.65×10^{8}	2.86×10^{8}	2.66×10^{8}	3.05×10^{8}	2.67×10^{8}	2.10×10^{8}	3.13×10^{8}	3.10×10^{8}	1.99×10^{8}	1.06×10^{8}	3.22×10^{8}	2.92×10^{8}	2.47×10^{8}	1.05×10^{8}
106	γ-Butyrolactone	5.23×10^{7}	1.43×10^{8}	1.83×10^{8}	2.80×10^{8}	2.18×10^{8}	1.75×10^{8}	2.66×10^{8}	7.01×10^{7}	1.12×10^{8}	1.00×10^{8}	5.88×10^{7}	2.84×10^{7}	8.67×10^{7}	8.39×10^{7}	5.23×10^{7}	3.54×10^{7}
107	2,3,4,5-Tetramethylfuran	8.70×10^{6}	1.06×10^{7}	1.18×10^{7}	9.08×10^{6}	3.75×10^{6}	1.36×10^{7}	1.13×10^{7}	6.08×10^{6}	2.89×10^{6}	7.77×10^{6}	4.13×10^{6}	3.62×10^{6}	8.83×10^{6}	8.52×10^{6}	5.17×10^{6}	1.69×10^{6}
108	3-(1,1-Dimethylethyl)-2,3-dihydrofuran	2.08×10^{5}	1.56×10^{5}	2.03×10^{5}	2.64×10^{5}	9.64×10^{4}	1.13×10^{5}	1.81×10^{5}	2.19×10^{5}	6.34×10^{5}	4.66×10^{5}	2.42×10^{5}	1.38×10^{5}	4.16×10^{5}	4.13×10^{5}	3.00×10^{5}	1.27×10^{5}
109	1-(2-Furyl)-2-propanone	1.12×10^{7}	6.56×10^{7}	7.19×10^{7}	4.56×10^{7}	1.66×10^{7}	7.99×10^{7}	4.21×10^{7}	6.11×10^{6}	7.33×10^{7}	7.85×10^{7}	2.89×10^{7}	2.31×10^{7}	7.70×10^{7}	7.58×10^{7}	5.26×10^{7}	1.27×10^{7}
110	2-Methyl-5-propenylfuran	6.56×10^{7}	7.83×10^{7}	8.67×10^{7}	7.89×10^{7}	4.07×10^{7}	1.03×10^{8}	9.07×10^{7}	5.52×10^{7}	2.97×10^{7}	5.43×10^{7}	3.17×10^{7}	2.22×10^{7}	5.78×10^{7}	5.66×10^{7}	3.37×10^{7}	1.35×10^{7}
111	Dihydro-5-methyl-2(3H)-furanone	8.32×10^{6}	9.93×10^{6}	1.07×10^{7}	1.16×10^{7}	1.22×10^{7}	1.11×10^{7}	1.50×10^{7}	8.86×10^{6}	1.27×10^{6}	2.28×10^{6}	1.47×10^{6}	2.00×10^{5}	1.44×10^{6}	1.77×10^{6}	1.07×10^{6}	3.03×10^{5}
112	5-Methylfurfural	5.15×10^{8}	5.33×10^{8}	7.10×10^{8}	6.94×10^{8}	5.59×10^{8}	4.92×10^{8}	7.25×10^{8}	4.72×10^{8}	7.37×10^{8}	6.62×10^{8}	4.64×10^{8}	2.53×10^{8}	6.80×10^{8}	6.77×10^{8}	6.06×10^{8}	2.76×10^{8}
113	2-Acetyl-5-methylfuran	2.51×10^{7}	2.68×10^{7}	2.78×10^{7}	2.59×10^{7}	1.94×10^{7}	3.18×10^{7}	3.28×10^{7}	2.11×10^{7}	2.88×10^{7}	3.88×10^{7}	1.67×10^{7}	7.92×10^{6}	3.56×10^{7}	3.67×10^{7}	2.35×10^{7}	8.89×10^{6}
114	Methyl 2-furoate	1.34×10^{7}	1.47×10^{7}	1.10×10^{7}	1.18×10^{7}	1.11×10^{7}	1.26×10^{7}	1.67×10^{7}	1.17×10^{7}	1.56×10^{7}	2.16×10^{7}	8.90×10^{6}	4.44×10^{6}	1.80×10^{7}	1.94×10^{7}	1.11×10^{7}	4.54×10^{6}
115	2-Pentylfuran	8.37×10^{7}	4.86×10^{7}	4.86×10^{7}	5.00×10^{7}	6.14×10^{7}	8.47×10^{7}	6.04×10^{7}	4.78×10^{7}	2.51×10^{7}	1.53×10^{7}	1.06×10^{7}	5.79×10^{6}	3.38×10^{7}	2.82×10^{7}	1.13×10^{7}	4.05×10^{6}
116	Benzofuran	1.40×10^{7}	1.52×10^{7}	2.06×10^{7}	1.41×10^{7}	1.05×10^{7}	1.79×10^{7}	1.78×10^{7}	1.23×10^{7}	2.75×10^{7}	3.50×10^{7}	1.94×10^{7}	1.11×10^{7}	3.44×10^{7}	3.32×10^{7}	2.14×10^{7}	7.66×10^{6}
117	Furfuryl acetate	4.01×10^{8}	4.79×10^{8}	6.42×10^{8}	6.10×10^{8}	5.50×10^{8}	4.47×10^{8}	5.71×10^{8}	4.52×10^{8}	7.84×10^{8}	8.96×10^{8}	7.55×10^{8}	5.55×10^{8}	8.89×10^{8}	8.07×10^{8}	9.17×10^{8}	6.56×10^{8}
118	2,5-Dihydro-3,5-dimethyl-2-furanone	8.06×10^{7}	9.89×10^{7}	1.08×10^{8}	1.15×10^{8}	1.15×10^{8}	1.14×10^{8}	1.10×10^{8}	8.88×10^{7}	2.06×10^{7}	2.58×10^{7}	1.29×10^{7}	5.42×10^{6}	2.08×10^{7}	2.27×10^{7}	1.44×10^{7}	6.07×10^{6}
119	1-(2-Furanyl)-1-propanone	8.09×10^{7}	7.14×10^{7}	8.08×10^{7}	1.04×10^{8}	9.99×10^{7}	7.57×10^{7}	9.52×10^{7}	8.49×10^{7}	9.65×10^{7}	1.05×10^{8}	5.68×10^{7}	2.49×10^{7}	9.34×10^{7}	5.33×10^{7}	7.27×10^{7}	2.61×10^{7}
120	2,2'-Bifuran	4.46×10^{7}	5.21×10^{7}	6.61×10^{7}	5.64×10^{7}	5.15×10^{7}	1.25×10^{8}	7.34×10^{7}	4.51×10^{7}	7.83×10^{7}	9.63×10^{7}	6.73×10^{7}	3.60×10^{7}	8.00×10^{7}	9.01×10^{7}	5.85×10^{7}	2.79×10^{7}
121	3,4-Dimethyl-2,5-furandione	9.92×10^{7}	1.02×10^{8}	1.16×10^{8}	1.15×10^{8}	1.29×10^{8}	1.37×10^{8}	1.45×10^{8}	9.09×10^{7}	2.56×10^{6}	2.98×10^{6}	1.18×10^{6}	6.11×10^{5}	2.60×10^{6}	2.60×10^{6}	2.06×10^{6}	6.62×10^{5}
122	1-(5-Methyl-2-furyl)-2-propanone	1.89×10^{6}	3.31×10^{7}	3.85×10^{7}	2.16×10^{7}	3.37×10^{6}	4.94×10^{7}	6.36×10^{6}	2.05×10^{6}	4.57×10^{7}	5.52×10^{7}	1.91×10^{7}	1.48×10^{7}	3.67×10^{7}	4.49×10^{7}	2.59×10^{7}	1.12×10^{7}
123	1-(2-Furanyl)-2-butanone	3.13×10^{6}	1.37×10^{7}	1.45×10^{7}	9.00×10^{6}	3.99×10^{6}	2.01×10^{7}	8.61×10^{6}	5.85×10^{5}	2.23×10^{7}	2.11×10^{7}	9.33×10^{6}	6.07×10^{6}	1.58×10^{7}	1.97×10^{7}	1.33×10^{7}	4.27×10^{6}
124	5-Ethyl-2-furaldehyde	1.06×10^{7}	1.35×10^{7}	1.59×10^{7}	1.43×10^{7}	1.64×10^{7}	1.50×10^{7}	1.98×10^{7}	1.05×10^{7}	1.09×10^{7}	1.38×10^{7}	7.32×10^{6}	2.51×10^{6}	1.32×10^{7}	1.41×10^{7}	9.04×10^{6}	2.67×10^{6}
125	1-(2-Furanyl)-3-butanone	6.98×10^{7}	6.10×10^{7}	7.93×10^{7}	6.25×10^{7}	8.88×10^{7}	1.05×10^{8}	8.07×10^{7}	4.84×10^{7}	6.23×10^{7}	6.70×10^{7}	2.78×10^{7}	1.75×10^{7}	5.64×10^{7}	5.38×10^{7}	4.13×10^{7}	1.85×10^{7}
126	2,2′-Methylenebisfuran	1.70×10^{8}	1.64×10^{8}	1.82×10^{8}	1.74×10^{8}	1.65×10^{8}	2.23×10^{8}	1.97×10^{8}	1.12×10^{8}	2.35×10^{8}	3.11×10^{8}	2.04×10^{8}	1.59×10^{8}	2.83×10^{8}	2.53×10^{8}	1.53×10^{8}	1.39×10^{8}
127	3-Acetyl-2,5-dimethylfuran	4.28×10^{6}	4.49×10^{6}	6.18×10^{6}	4.75×10^{6}	4.99×10^{6}	6.11×10^{6}	6.28×10^{6}	4.22×10^{6}	5.78×10^{6}	6.95×10^{6}	3.31×10^{6}	2.18×10^{6}	6.64×10^{6}	5.55×10^{6}	3.68×10^{6}	1.97×10^{6}

128	Furfuryl propanoate	5.96×10^{7}	6.58×10^{7}	6.24×10^{7}	6.11×10^{7}	7.08×10^{7}	8.48×10^{7}	6.27×10^{7}	5.86×10^{7}	9.91×10^{7}	1.18×10^{8}	7.75×10^{7}	3.81×10^{7}	9.60×10^{7}	1.26×10^{8}	8.54×10^{7}	4.23×10^{7}
129	1-(2-Furanyl)-1-butanone	9.81×10^{6}	8.26×10^{6}	1.36×10^{7}	7.64×10^{6}	1.07×10^{7}	1.22×10^{7}	1.20×10^{7}	9.38×10^{6}	1.13×10^{7}	1.37×10^{7}	7.09×10^{6}	2.74×10^{6}	1.27×10^{7}	1.36×10^{7}	8.00×10^{6}	2.67×10^{6}
130	2-Methylbenzofuran	1.88×10^{7}	2.21×10^{7}	2.36×10^{7}	2.18×10^{7}	2.45×10^{7}	3.12×10^{7}	2.58×10^{7}	1.94×10^{7}	3.05×10^{7}	2.58×10^{7}	1.64×10^{7}	1.19×10^{7}	3.29×10^{7}	3.43×10^{7}	2.06×10^{7}	6.69×10^{6}
131	2-Methyl-5-propionylfuran	3.16×10^{7}	3.52×10^{7}	3.89×10^{7}	3.64×10^{7}	3.91×10^{7}	4.99×10^{7}	4.82×10^{7}	3.28×10^{7}	2.05×10^{7}	2.74×10^{7}	1.28×10^{7}	4.85×10^{6}	2.13×10^{7}	2.64×10^{7}	1.53×10^{7}	5.23×10^{6}
132	1-(5-Methyl-2-furanyl)-2-butanone	1.36×10^{6}	5.23×10^{6}	7.02×10^{6}	6.11×10^{6}	1.79×10^{6}	9.50×10^{6}	1.78×10^{6}	2.02×10^{6}	7.51×10^{6}	1.18×10^{7}	3.95×10^{6}	2.56×10^{6}	6.58×10^{6}	9.66×10^{6}	4.01×10^{6}	1.16×10^{6}
133	2-Heptylfuran	1.04×10^{7}	1.48×10^{7}	1.53×10^{7}	1.44×10^{7}	9.58×10^{6}	1.79×10^{7}	1.64×10^{7}	1.01×10^{7}	1.35×10^{7}	1.74×10^{7}	7.28×10^{6}	3.99×10^{6}	1.51×10^{7}	1.49×10^{7}	1.06×10^{7}	3.49×10^{6}
134	Furfuryl butanoate	3.24×10^{6}	3.29×10^{6}	3.76×10^{6}	4.11×10^{6}	5.57×10^{6}	4.49×10^{6}	4.69×10^{6}	3.34×10^{6}	5.88×10^{6}	7.52×10^{6}	5.91×10^{6}	1.96×10^{6}	6.41×10^{6}	6.71×10^{6}	3.77×10^{6}	2.34×10^{6}
135	2-(2-Furanylmethyl)-5-methylfuran	8.46×10^{7}	8.14×10^{7}	8.54×10^{7}	8.92×10^{7}	8.23×10^{7}	1.07×10^{8}	8.58×10^{7}	6.83×10^{7}	1.04×10^{8}	1.11×10^{8}	7.58×10^{7}	5.50×10^{7}	1.03×10^{8}	9.71×10^{7}	7.09×10^{7}	4.20×10^{7}
136	*n*-Furfuryl pyrrole	2.64×10^{8}	1.82×10^{8}	2.34×10^{8}	1.83×10^{8}	2.29×10^{8}	1.91×10^{8}	2.13×10^{8}	1.94×10^{8}	4.62×10^{8}	3.84×10^{8}	2.65×10^{8}	2.14×10^{8}	4.23×10^{8}	3.29×10^{8}	2.26×10^{8}	1.91×10^{8}
137	2-Methyl-3(2-furyl)acrolein	1.42×10^{7}	1.23×10^{7}	1.26×10^{7}	1.63×10^{7}	1.47×10^{7}	1.60×10^{7}	1.53×10^{7}	1.19×10^{7}	2.15×10^{7}	1.82×10^{7}	9.20×10^{6}	4.43×10^{6}	1.78×10^{7}	1.77×10^{7}	1.04×10^{7}	3.70×10^{6}
138	4-(2-Furanyl)-3-buten-2-one	1.67×10^{7}	1.53×10^{7}	1.66×10^{7}	1.99×10^{7}	2.02×10^{7}	1.97×10^{7}	1.99×10^{7}	1.47×10^{7}	1.27×10^{7}	1.27×10^{7}	6.10×10^{6}	1.97×10^{6}	1.20×10^{7}	1.22×10^{7}	7.26×10^{6}	2.68×10^{6}
139	4,7-Dimethylbenzofuran	1.08×10^{7}	1.34×10^{7}	1.36×10^{7}	1.38×10^{7}	1.23×10^{7}	1.45×10^{7}	1.81×10^{7}	2.42×10^{6}	7.31×10^{6}	7.10×10^{6}	4.02×10^{6}	2.16×10^{6}	6.12×10^{6}	6.13×10^{6}	4.99×10^{6}	1.30×10^{6}
140	2-Furyl pyrazine	3.16×10^{7}	2.94×10^{7}	3.07×10^{7}	4.23×10^{7}	2.96×10^{7}	2.90×10^{7}	2.19×10^{7}	3.40×10^{7}	1.76×10^{7}	1.77×10^{7}	7.89×10^{6}	3.92×10^{6}	1.33×10^{7}	1.22×10^{7}	6.23×10^{6}	4.64×10^{6}
141	2,2′-Methylenebis(5-methylfuran)	6.81×10^{6}	7.23×10^{6}	7.22×10^{6}	7.51×10^{6}	7.89×10^{6}	9.29×10^{6}	9.41×10^{6}	6.98×10^{6}	9.58×10^{6}	7.60×10^{6}	5.04×10^{6}	4.01×10^{6}	7.50×10^{6}	8.69×10^{6}	5.35×10^{6}	2.87×10^{6}
142	1-(5-Methylfurfuryl)pyrrole	1.28×10^{7}	1.14×10^{7}	9.15×10^{6}	1.14×10^{7}	9.67×10^{6}	1.07×10^{7}	6.48×10^{6}	1.07×10^{7}	3.43×10^{7}	2.55×10^{7}	1.21×10^{7}	8.94×10^{6}	1.91×10^{7}	1.66×10^{7}	7.19×10^{6}	6.98×10^{6}
143	Difurfuryl ether	7.21×10^{7}	6.39×10^{7}	6.38×10^{7}	7.85×10^{7}	7.36×10^{7}	8.59×10^{7}	7.31×10^{7}	5.99×10^{7}	1.20×10^{8}	1.23×10^{8}	6.30×10^{7}	3.22×10^{7}	1.13×10^{8}	1.16×10^{8}	5.96×10^{7}	3.35×10^{7}
144	1-Furfuryl-2-formylpyrrole	6.65×10^{5}	4.39×10^{5}	1.18×10^{6}	1.60×10^{6}	9.39×10^{5}	1.27×10^{6}	1.21×10^{6}	1.09×10^{6}	1.86×10^{6}	1.85×10^{6}	7.47×10^{5}	5.22×10^{5}	1.12×10^{6}	1.41×10^{6}	8.01×10^{5}	4.16×10^{5}
145	2-Acetyl-1-furfurylpyrrole	2.57×10^{6}	1.86×10^{6}	1.89×10^{6}	2.42×10^{6}	2.76×10^{6}	3.09×10^{6}	2.52×10^{6}	2.52×10^{6}	6.93×10^{6}	5.55×10^{6}	2.87×10^{6}	1.28×10^{6}	5.00×10^{6}	4.89×10^{6}	2.50×10^{6}	1.30×10^{6}
	**Hydrocarbons**	6.00×10^{7} (7.2)	9.14×10^{7} (16.4)	7.69×10^{7} (19.9)	6.10×10^{7} (9.0)	6.53×10^{7} (37.1)	9.09×10^{7} (20.9)	5.15×10^{7} (3.3)	6.14×10^{7} (17.6)	1.97×10^{8} (35.7)	2.68×10^{8} (34.0)	1.93×10^{8} (32.3)	3.94×10^{7} (5.7)	2.64×10^{8} (13.9)	2.24×10^{8} (21.9)	1.25×10^{8} (42.8)	1.58×10^{8} (13.0)
	Aliphatics																
146	Nonane	4.71×10^{5}	7.76×10^{5}	6.76×10^{5}	7.71×10^{5}	4.60×10^{5}	1.76×10^{6}	6.74×10^{5}	3.99×10^{5}	2.29×10^{5}	2.48×10^{5}	1.84×10^{5}	6.60×10^{4}	2.25×10^{5}	1.50×10^{5}	1.11×10^{5}	4.55×10^{4}
147	1,3-Nonadiene	2.54×10^{6}	1.97×10^{6}	1.74×10^{6}	1.42×10^{6}	6.77×10^{5}	2.19×10^{6}	1.05×10^{6}	1.25×10^{6}	1.08×10^{5}	1.85×10^{5}	1.30×10^{5}	9.28×10^{4}	2.81×10^{5}	2.36×10^{5}	8.61×10^{4}	5.36×10^{4}
148	Decane	8.88×10^{5}	1.39×10^{6}	7.46×10^{5}	6.58×10^{5}	1.90×10^{6}	3.18×10^{6}	1.49×10^{6}	1.35×10^{6}	1.50×10^{6}	2.32×10^{6}	2.01×10^{6}	1.26×10^{5}	1.03×10^{6}	1.03×10^{6}	9.53×10^{5}	7.53×10^{5}
149	Undecane	2.37×10^{6}	1.20×10^{7}	7.44×10^{6}	1.51×10^{6}	2.07×10^{7}	3.10×10^{7}	3.14×10^{6}	4.00×10^{6}	2.06×10^{6}	2.49×10^{6}	2.58×10^{6}	4.69×10^{5}	1.48×10^{6}	1.78×10^{6}	1.44×10^{6}	9.85×10^{5}
150	1-Dodecene	5.83×10^{5}	4.90×10^{5}	1.95×10^{6}	3.34×10^{5}	9.28×10^{5}	5.90×10^{5}	4.42×10^{5}	2.30×10^{6}	3.54×10^{5}	5.87×10^{5}	2.58×10^{5}	1.88×10^{5}	5.62×10^{5}	5.25×10^{5}	2.34×10^{5}	1.82×10^{5}
151	Dodecane	1.71×10^{6}	2.37×10^{6}	1.50×10^{6}	1.43×10^{6}	4.78×10^{6}	4.23×10^{5}	2.98×10^{6}	4.57×10^{6}	5.30×10^{6}	1.10×10^{7}	4.32×10^{6}	2.73×10^{5}	6.28×10^{6}	7.55×10^{6}	6.65×10^{6}	5.40×10^{6}
152	Tridecane	1.42×10^{6}	1.75×10^{6}	1.24×10^{6}	1.38×10^{6}	2.03×10^{6}	1.74×10^{6}	1.24×10^{6}	2.40×10^{6}	3.76×10^{7}	6.67×10^{7}	4.77×10^{7}	4.46×10^{5}	5.70×10^{7}	4.81×10^{7}	3.15×10^{7}	4.20×10^{7}
153	Tetradecane	2.10×10^{6}	1.86×10^{6}	2.19×10^{6}	1.59×10^{6}	1.94×10^{6}	1.41×10^{6}	1.26×10^{6}	2.45×10^{6}	5.10×10^{7}	5.58×10^{7}	5.09×10^{7}	1.69×10^{6}	7.57×10^{7}	7.00×10^{7}	3.37×10^{7}	5.46×10^{7}
154	Pentadecane	2.11×10^{6}	1.51×10^{6}	4.08×10^{5}	8.46×10^{5}	9.85×10^{5}	7.25×10^{5}	6.49×10^{5}	1.27×10^{6}	2.32×10^{6}	2.77×10^{6}	1.47×10^{6}	8.50×10^{5}	3.10×10^{6}	1.93×10^{6}	1.51×10^{6}	1.55×10^{6}

155	Hexadecane	1.23×10^{6}	1.35×10^{6}	5.15×10^{5}	6.88×10^{4}	5.54×10^{5}	4.10×10^{5}	2.56×10^{5}	5.99×10^{5}	2.90×10^{6}	3.09×10^{6}	1.95×10^{6}	8.87×10^{5}	4.79×10^{6}	4.87×10^{6}	3.09×10^{6}	3.60×10^{6}
	Aromatics																
156	Methylbenzene	5.71×10^{6}	1.11×10^{7}	1.22×10^{7}	6.71×10^{6}	2.11×10^{6}	1.08×10^{7}	6.34×10^{6}	2.77×10^{6}	2.55×10^{7}	2.86×10^{7}	1.32×10^{7}	1.07×10^{7}	2.71×10^{7}	2.02×10^{7}	6.39×10^{6}	6.17×10^{6}
157	Ethylbenzene	2.03×10^{6}	3.14×10^{6}	2.94×10^{6}	2.40×10^{6}	9.58×10^{5}	1.77×10^{6}	1.40×10^{6}	1.11×10^{6}	2.57×10^{6}	2.56×10^{6}	3.38×10^{6}	1.86×10^{6}	3.51×10^{6}	2.13×10^{6}	1.09×10^{6}	9.88×10^{5}
158	1,3-Dimethylbenzene	5.18×10^{6}	5.64×10^{6}	6.09×10^{6}	4.94×10^{6}	3.28×10^{6}	4.74×10^{6}	4.29×10^{6}	2.93×10^{6}	7.88×10^{6}	1.13×10^{7}	8.55×10^{6}	5.33×10^{6}	9.66×10^{6}	6.71×10^{6}	4.20×10^{6}	3.94×10^{6}
159	Phenylethylene	1.86×10^{7}	2.54×10^{7}	2.16×10^{7}	2.02×10^{7}	9.01×10^{6}	1.66×10^{7}	1.20×10^{7}	1.57×10^{7}	1.78×10^{7}	2.88×10^{7}	1.52×10^{7}	1.25×10^{7}	2.38×10^{7}	1.64×10^{7}	7.34×10^{6}	6.68×10^{6}
160	1-Methylethylbenzene	2.18×10^{5}	3.97×10^{5}	2.08×10^{5}	1.71×10^{5}	6.82×10^{4}	1.06×10^{4}	2.23×10^{5}	1.01×10^{6}	1.02×10^{5}	1.33×10^{5}	1.12×10^{5}	6.21×10^{4}	1.59×10^{5}	2.25×10^{5}	7.10×10^{4}	3.79×10^{4}
161	1-Ethyl-4-methylbenzene	2.09×10^{6}	2.59×10^{6}	2.76×10^{6}	2.21×10^{6}	1.87×10^{6}	2.41×10^{6}	3.19×10^{6}	2.30×10^{6}	8.72×10^{5}	1.39×10^{6}	1.63×10^{6}	7.82×10^{5}	2.15×10^{6}	1.64×10^{6}	1.27×10^{6}	6.14×10^{5}
162	1,2,3-Trimethylbenzene	1.95×10^{6}	2.34×10^{6}	2.23×10^{6}	2.49×10^{6}	1.85×10^{6}	1.34×10^{6}	1.76×10^{6}	2.27×10^{6}	1.24×10^{6}	1.47×10^{6}	9.97×10^{5}	9.55×10^{5}	1.60×10^{6}	1.62×10^{6}	7.20×10^{5}	6.80×10^{5}
163	1-Methyl-3-propylbenzene	5.72×10^{5}	1.58×10^{6}	2.22×10^{5}	8.20×10^{5}	1.22×10^{6}	7.95×10^{5}	2.01×10^{5}	1.35×10^{6}	1.67×10^{5}	2.85×10^{5}	2.46×10^{5}	7.50×10^{4}	2.70×10^{5}	1.87×10^{5}	1.18×10^{5}	2.46×10^{5}
164	Butylbenzene	3.38×10^{6}	3.50×10^{6}	3.27×10^{6}	3.35×10^{6}	2.04×10^{6}	3.01×10^{6}	2.21×10^{6}	2.82×10^{6}	6.50×10^{5}	7.44×10^{5}	6.20×10^{5}	3.62×10^{5}	1.04×10^{6}	1.06×10^{6}	4.39×10^{5}	3.59×10^{5}
165	4-Ethyl-1,2-dimethylbenzene	4.03×10^{5}	1.60×10^{6}	1.10×10^{6}	9.20×10^{5}	1.22×10^{6}	1.15×10^{6}	1.18×10^{6}	1.34×10^{6}	2.60×10^{5}	6.88×10^{5}	3.95×10^{5}	1.74×10^{5}	6.38×10^{5}	5.25×10^{5}	3.62×10^{5}	1.85×10^{5}
166	1-Ethyl-2,3-dimethylbenzene	5.24×10^{5}	1.33×10^{6}	7.94×10^{5}	1.09×10^{6}	7.99×10^{5}	6.62×10^{5}	7.60×10^{5}	9.91×10^{5}	1.67×10^{5}	4.22×10^{5}	2.33×10^{5}	1.21×10^{5}	3.55×10^{5}	2.36×10^{5}	1.79×10^{5}	1.86×10^{5}
167	1,2,4,5-Tetramethylbenzene	4.10×10^{5}	3.05×10^{6}	2.02×10^{6}	2.30×10^{6}	2.43×10^{6}	6.72×10^{5}	1.93×10^{6}	2.53×10^{6}	3.80×10^{5}	1.46×10^{6}	8.61×10^{5}	4.32×10^{5}	1.20×10^{6}	5.36×10^{5}	5.55×10^{5}	3.98×10^{5}
168	1,2,3,4-Tetramethylbenzene	7.63×10^{5}	1.52×10^{6}	1.02×10^{6}	1.19×10^{6}	1.19×10^{6}	9.79×10^{5}	1.02×10^{6}	1.46×10^{6}	5.39×10^{5}	8.70×10^{5}	4.96×10^{5}	3.14×10^{5}	8.40×10^{5}	5.74×10^{5}	3.83×10^{5}	2.80×10^{5}
169	Pentylbenzene	2.50×10^{6}	2.70×10^{6}	2.00×10^{6}	2.16×10^{6}	2.12×10^{6}	2.37×10^{6}	1.83×10^{6}	2.29×10^{6}	4.23×10^{5}	5.64×10^{5}	3.36×10^{5}	1.84×10^{5}	4.66×10^{5}	4.30×10^{5}	2.05×10^{5}	1.49×10^{5}
170	1-Butylheptylbenzene	1.54×10^{5}	3.51×10^{4}	3.27×10^{4}	6.28×10^{3}	2.06×10^{5}	1.29×10^{5}	1.68×10^{4}	1.07×10^{4}	3.57×10^{7}	4.34×10^{7}	3.51×10^{7}	4.67×10^{5}	4.05×10^{7}	3.53×10^{7}	2.23×10^{7}	2.81×10^{7}
	**Ketones**	1.46×10^{9} (3.3)	1.78×10^{9} (14.2)	2.23×10^{9} (24.4)	1.76×10^{9} (6.0)	1.52×10^{9} (9.5)	1.78×10^{9} (6.7)	1.87×10^{9} (10.4)	1.25×10^{9} (29.9)	9.81×10^{8} (14.7)	1.02×10^{9} (4.4)	5.19×10^{8} (4.2)	3.81×10^{8} (16.4)	9.43×10^{8} (11.6)	9.29×10^{8} (12.7)	6.35×10^{8} (12.8)	3.45×10^{8} (15.0)
	Aliphatics																
171	2-Propanone	9.13×10^{7}	5.14×10^{7}	6.54×10^{7}	7.90×10^{7}	1.98×10^{7}	7.92×10^{7}	5.45×10^{7}	4.11×10^{7}	7.14×10^{7}	6.37×10^{7}	3.52×10^{7}	4.33×10^{7}	5.47×10^{7}	5.00×10^{7}	4.27×10^{7}	4.24×10^{7}
172	2-Butanone	4.00×10^{7}	3.96×10^{7}	3.54×10^{7}	3.53×10^{7}	1.56×10^{7}	4.71×10^{7}	3.64×10^{7}	2.44×10^{7}	7.23×10^{7}	6.83×10^{7}	4.61×10^{7}	4.12×10^{7}	3.72×10^{7}	4.04×10^{7}	4.57×10^{7}	3.42×10^{7}
173	2,3-Butanedione (Diacetyl)	3.16×10^{7}	3.63×10^{7}	3.85×10^{7}	4.54×10^{7}	1.30×10^{7}	3.99×10^{7}	3.23×10^{7}	1.96×10^{7}	5.53×10^{7}	5.18×10^{7}	2.40×10^{7}	2.28×10^{7}	3.58×10^{7}	4.03×10^{7}	3.21×10^{7}	1.77×10^{7}
174	2-Pentanone	8.43×10^{6}	1.05×10^{7}	1.30×10^{7}	6.14×10^{6}	2.12×10^{6}	1.30×10^{7}	8.03×10^{6}	4.33×10^{6}	2.78×10^{7}	2.39×10^{7}	1.55×10^{7}	1.09×10^{7}	2.43×10^{7}	2.17×10^{7}	1.32×10^{7}	7.49×10^{6}
175	2,3-Pentanedione	5.92×10^{7}	6.24×10^{7}	1.00×10^{8}	6.33×10^{7}	1.97×10^{7}	7.86×10^{7}	7.09×10^{7}	3.71×10^{7}	8.75×10^{7}	9.99×10^{7}	5.97×10^{7}	5.56×10^{7}	7.97×10^{7}	8.98×10^{7}	7.11×10^{7}	4.05×10^{7}
176	1-Hydroxy-2-propanone	2.52×10^{8}	1.91×10^{8}	1.62×10^{8}	2.75×10^{8}	2.41×10^{8}	2.56×10^{8}	2.95×10^{8}	2.60×10^{8}	2.51×10^{7}	2.55×10^{7}	1.41×10^{7}	4.11×10^{6}	2.44×10^{7}	1.98×10^{7}	1.65×10^{7}	6.15×10^{6}
177	4-Methyl-2-pentanone	3.75×10^{5}	7.22×10^{5}	1.08×10^{6}	5.50×10^{5}	4.05×10^{5}	1.28×10^{6}	4.58×10^{5}	1.06×10^{5}	3.22×10^{6}	6.38×10^{6}	2.33×10^{6}	1.10×10^{6}	5.15×10^{6}	2.91×10^{6}	1.35×10^{6}	1.01×10^{6}
178	3-Penten-2-one	1.35×10^{7}	1.58×10^{7}	1.36×10^{7}	1.76×10^{7}	5.53×10^{6}	1.55×10^{7}	1.08×10^{7}	1.12×10^{7}	1.34×10^{7}	1.15×10^{7}	9.25×10^{6}	4.73×10^{6}	9.85×10^{6}	1.23×10^{7}	7.04×10^{6}	5.86×10^{6}
179	3-Hydroxy-2-butanone (Acetoin)	8.26×10^{7}	9.12×10^{7}	8.12×10^{7}	9.21×10^{7}	5.69×10^{7}	1.10×10^{8}	1.16×10^{8}	5.70×10^{7}	9.19×10^{6}	7.59×10^{6}	4.50×10^{6}	2.25×10^{6}	7.03×10^{6}	7.50×10^{6}	5.22×10^{6}	2.36×10^{6}

180	2-Methyl-1-penten-3-one	3.91×10^{4}	6.96×10^{6}	1.81×10^{6}	1.62×10^{5}	3.91×10^{4}	2.85×10^{6}	7.18×10^{5}	3.91×10^{4}	4.05×10^{6}	5.58×10^{6}	2.36×10^{6}	1.58×10^{6}	4.45×10^{6}	4.68×10^{6}	1.69×10^{6}	1.13×10^{6}
181	2,4-Pentanedione	1.55×10^{6}	2.05×10^{6}	2.65×10^{6}	1.84×10^{6}	6.22×10^{5}	2.38×10^{6}	1.74×10^{6}	9.45×10^{5}	1.03×10^{6}	1.19×10^{6}	4.19×10^{4}	4.19×10^{4}	1.36×10^{5}	1.58×10^{5}	4.19×10^{4}	4.19×10^{4}
182	3-Hexanone	7.92×10^{6}	1.23×10^{7}	1.55×10^{7}	8.94×10^{6}	3.50×10^{6}	1.64×10^{7}	7.41×10^{6}	4.60×10^{6}	3.55×10^{7}	3.88×10^{7}	2.13×10^{7}	1.66×10^{7}	4.02×10^{7}	3.61×10^{7}	1.81×10^{7}	1.15×10^{7}
183	2,4-Dimethyl-3-pentanone	1.07×10^{7}	2.71×10^{7}	2.41×10^{7}	1.54×10^{7}	2.81×10^{6}	2.72×10^{7}	1.78×10^{7}	6.69×10^{6}	3.86×10^{7}	4.75×10^{7}	2.34×10^{7}	1.85×10^{7}	4.98×10^{7}	4.73×10^{7}	3.50×10^{7}	1.39×10^{7}
184	2-Hexanone	2.04×10^{6}	2.47×10^{6}	2.90×10^{6}	2.05×10^{6}	8.66×10^{5}	2.88×10^{6}	1.66×10^{6}	1.07×10^{6}	5.88×10^{6}	3.86×10^{6}	2.38×10^{6}	2.19×10^{6}	7.54×10^{6}	6.26×10^{6}	3.04×10^{6}	1.80×10^{6}
185	3,4-Hexanedione	1.07×10^{7}	2.11×10^{7}	2.34×10^{7}	1.41×10^{7}	3.99×10^{6}	2.53×10^{7}	1.81×10^{7}	6.01×10^{6}	2.55×10^{7}	3.57×10^{7}	1.89×10^{7}	1.15×10^{7}	3.59×10^{7}	3.27×10^{7}	2.71×10^{7}	8.27×10^{6}
186	1-Hydroxy-2-butanone	4.26×10^{7}	4.81×10^{7}	5.16×10^{7}	5.33×10^{7}	5.69×10^{7}	5.95×10^{7}	6.01×10^{7}	4.18×10^{7}	4.37×10^{6}	3.88×10^{6}	2.29×10^{6}	9.23×10^{5}	3.65×10^{6}	3.32×10^{6}	2.70×10^{6}	6.39×10^{5}
187	3-Hydroxy-2-pentanone	2.72×10^{7}	3.85×10^{7}	4.72×10^{7}	3.73×10^{7}	2.89×10^{7}	5.51×10^{7}	4.80×10^{7}	2.32×10^{7}	5.57×10^{6}	6.11×10^{6}	3.25×10^{6}	1.57×10^{6}	5.22×10^{6}	5.37×10^{6}	4.34×10^{6}	1.23×10^{6}
188	2-Hydroxy-3-pentanone	2.41×10^{7}	3.20×10^{7}	4.36×10^{7}	3.54×10^{7}	2.79×10^{7}	4.80×10^{7}	4.51×10^{7}	1.98×10^{7}	5.15×10^{6}	5.58×10^{6}	1.60×10^{6}	1.35×10^{6}	4.38×10^{6}	4.61×10^{6}	3.39×10^{6}	8.25×10^{5}
189	3-Hexen-2-one	4.82×10^{6}	6.60×10^{6}	5.96×10^{6}	4.91×10^{6}	1.91×10^{6}	6.62×10^{6}	5.11×10^{6}	3.18×10^{6}	6.46×10^{6}	1.42×10^{7}	4.05×10^{6}	2.39×10^{6}	1.60×10^{7}	8.88×10^{6}	5.81×10^{6}	2.35×10^{6}
190	3-Hexene-2,5-dione	3.27×10^{6}	8.61×10^{6}	6.59×10^{6}	5.47×10^{6}	7.40×10^{5}	6.18×10^{6}	3.09×10^{6}	2.71×10^{6}	6.86×10^{6}	2.00×10^{7}	6.32×10^{6}	6.10×10^{6}	1.15×10^{7}	1.10×10^{7}	4.54×10^{6}	3.06×10^{6}
191	4-Methyl-2-hexanone	7.90×10^{5}	1.09×10^{6}	1.45×10^{6}	9.13×10^{5}	3.27×10^{6}	1.06×10^{6}	9.80×10^{5}	4.52×10^{5}	1.94×10^{6}	3.06×10^{6}	1.59×10^{6}	9.81×10^{5}	2.58×10^{6}	2.34×10^{6}	1.09×10^{6}	1.05×10^{6}
192	4-Heptanone	3.04×10^{5}	5.48×10^{5}	4.19×10^{5}	3.01×10^{5}	1.76×10^{5}	4.36×10^{5}	3.29×10^{5}	2.43×10^{5}	8.53×10^{5}	1.22×10^{6}	6.87×10^{5}	3.35×10^{5}	1.15×10^{6}	1.16×10^{6}	5.44×10^{5}	2.72×10^{5}
193	1-(Acetyloxy)-2-propanone	2.78×10^{8}	5.84×10^{8}	9.64×10^{8}	3.92×10^{8}	4.75×10^{8}	2.70×10^{8}	4.56×10^{8}	2.59×10^{8}	2.22×10^{8}	1.72×10^{8}	5.07×10^{7}	5.14×10^{7}	1.66×10^{8}	1.73×10^{8}	1.15×10^{8}	6.08×10^{7}
194	3-Heptanone	1.41×10^{6}	9.72×10^{5}	1.17×10^{6}	9.23×10^{5}	4.71×10^{5}	1.18×10^{6}	7.97×10^{5}	8.38×10^{5}	1.68×10^{6}	1.97×10^{6}	1.30×10^{6}	7.59×10^{5}	3.24×10^{6}	2.84×10^{6}	1.31×10^{6}	7.46×10^{5}
195	2-Heptanone	9.15×10^{6}	9.04×10^{6}	1.17×10^{7}	8.48×10^{6}	7.79×10^{6}	1.98×10^{7}	1.06×10^{7}	5.86×10^{6}	1.43×10^{7}	1.91×10^{7}	1.54×10^{7}	7.16×10^{6}	5.24×10^{7}	3.99×10^{7}	1.56×10^{7}	7.45×10^{6}
196	3-Hepten-2-one	1.24×10^{6}	1.99×10^{6}	1.95×10^{6}	1.58×10^{6}	6.66×10^{5}	1.90×10^{6}	1.56×10^{6}	9.19×10^{5}	1.74×10^{6}	3.14×10^{6}	1.45×10^{6}	7.70×10^{5}	2.67×10^{6}	2.49×10^{6}	1.44×10^{6}	7.77×10^{5}
197	2,5-Hexanedione	3.81×10^{7}	5.17×10^{7}	5.87×10^{7}	5.61×10^{7}	6.94×10^{7}	6.46×10^{7}	7.10×10^{7}	3.87×10^{7}	3.49×10^{6}	3.79×10^{6}	2.41×10^{6}	9.37×10^{5}	4.08×10^{6}	4.04×10^{6}	2.80×10^{6}	9.71×10^{5}
198	6-Methyl-3-heptanone	1.55×10^{6}	1.06×10^{6}	1.16×10^{6}	1.10×10^{6}	3.25×10^{5}	7.63×10^{5}	1.05×10^{6}	7.94×10^{5}	1.11×10^{6}	2.22×10^{6}	1.16×10^{6}	9.74×10^{5}	1.84×10^{6}	1.67×10^{6}	6.91×10^{5}	6.78×10^{5}
199	6-Methyl-2-heptanone	2.23×10^{6}	2.36×10^{6}	2.43×10^{6}	1.92×10^{6}	6.82×10^{5}	1.86×10^{6}	1.27×10^{6}	1.59×10^{6}	2.88×10^{6}	3.89×10^{6}	2.43×10^{6}	1.78×10^{6}	3.64×10^{6}	3.17×10^{6}	1.62×10^{6}	1.61×10^{6}
200	5-Methyl-2-heptanone	5.77×10^{5}	5.90×10^{5}	5.20×10^{5}	2.31×10^{6}	2.01×10^{5}	4.79×10^{5}	4.73×10^{5}	4.39×10^{5}	7.93×10^{5}	9.26×10^{5}	5.13×10^{5}	3.94×10^{5}	7.77×10^{5}	6.66×10^{5}	3.17×10^{5}	3.42×10^{5}
201	1-Octen-3-one	3.18×10^{6}	1.31×10^{6}	1.43×10^{6}	2.07×10^{6}	1.82×10^{6}	1.75×10^{6}	1.60×10^{6}	2.60×10^{6}	3.19×10^{6}	6.67×10^{5}	2.11×10^{6}	6.74×10^{5}	3.86×10^{6}	2.66×10^{6}	1.28×10^{6}	1.14×10^{6}
202	2,3-Octanedione	1.15×10^{7}	6.31×10^{6}	5.33×10^{6}	8.74×10^{6}	6.57×10^{6}	5.29×10^{6}	9.27×10^{6}	1.02×10^{7}	1.26×10^{7}	9.43×10^{6}	9.88×10^{6}	3.60×10^{6}	1.45×10^{7}	1.12×10^{7}	6.89×10^{6}	5.50×10^{6}
203	6-Methyl-5-hepten-2-one	4.97×10^{6}	9.68×10^{6}	1.22×10^{7}	1.31×10^{7}	7.29×10^{6}	4.75×10^{6}	5.86×10^{6}	7.17×10^{6}	3.45×10^{6}	9.11×10^{6}	4.95×10^{6}	2.34×10^{6}	5.02×10^{6}	8.21×10^{6}	3.50×10^{6}	1.97×10^{6}
204	2-Octanone	3.20×10^{6}	4.57×10^{6}	4.69×10^{6}	8.01×10^{6}	6.09×10^{6}	9.19×10^{6}	3.64×10^{6}	6.37×10^{6}	6.26×10^{6}	1.11×10^{7}	4.34×10^{6}	4.55×10^{6}	1.33×10^{7}	1.09×10^{7}	5.13×10^{6}	3.68×10^{6}
205	3-Octen-2-one	3.42×10^{6}	2.33×10^{6}	2.69×10^{6}	2.52×10^{6}	4.71×10^{6}	4.63×10^{6}	4.03×10^{6}	2.26×10^{6}	2.18×10^{6}	2.75×10^{6}	2.54×10^{6}	7.90×10^{5}	5.65×10^{6}	5.90×10^{6}	2.41×10^{6}	9.18×10^{5}
206	2-Nonanone	9.48×10^{5}	1.28×10^{6}	1.37×10^{6}	1.31×10^{6}	1.17×10^{6}	1.17×10^{6}	9.96×10^{5}	1.46×10^{6}	1.09×10^{6}	1.34×10^{6}	1.15×10^{6}	6.92×10^{5}	1.42×10^{6}	1.27×10^{6}	6.26×10^{5}	8.36×10^{5}
207	3-Nonen-2-one	6.76×10^{5}	7.93×10^{5}	4.31×10^{5}	1.18×10^{6}	1.20×10^{6}	1.32×10^{6}	9.83×10^{5}	9.28×10^{5}	1.37×10^{6}	1.11×10^{6}	7.40×10^{5}	3.97×10^{5}	1.46×10^{6}	1.56×10^{6}	7.77×10^{5}	3.53×10^{5}
	Cyclics																

208	2-Cyclopenten-1-one	2.00×10^{7}	2.48×10^{7}	2.38×10^{7}	2.48×10^{7}	1.64×10^{7}	2.47×10^{7}	2.31×10^{7}	1.68×10^{7}	8.93×10^{6}	8.39×10^{6}	3.83×10^{6}	2.39×10^{6}	6.54×10^{6}	6.54×10^{6}	3.95×10^{6}	2.55×10^{6}
209	Cyclohexanone	3.21×10^{6}	4.04×10^{6}	3.86×10^{6}	3.32×10^{6}	1.56×10^{6}	4.10×10^{6}	3.29×10^{6}	2.39×10^{6}	5.30×10^{6}	5.71×10^{6}	3.57×10^{6}	1.05×10^{6}	3.76×10^{6}	3.62×10^{6}	2.20×10^{6}	6.63×10^{5}
210	4-Cyclopentene-1,3-dione	1.01×10^{7}	1.24×10^{7}	1.82×10^{7}	3.63×10^{7}	1.03×10^{7}	8.10×10^{6}	1.01×10^{7}	2.12×10^{7}	9.52×10^{6}	7.47×10^{6}	4.18×10^{6}	3.03×10^{6}	5.77×10^{6}	4.90×10^{6}	3.41×10^{6}	2.91×10^{6}
211	2-Methyl-2-cyclopenten-1-one	3.39×10^{7}	4.46×10^{7}	3.05×10^{7}	4.24×10^{7}	2.91×10^{7}	5.27×10^{7}	4.73×10^{7}	2.94×10^{7}	2.32×10^{7}	2.54×10^{7}	1.20×10^{7}	6.22×10^{6}	2.19×10^{7}	2.23×10^{7}	1.52×10^{7}	6.36×10^{6}
212	2-Cyclohexen-1-one	1.63×10^{7}	1.65×10^{7}	1.68×10^{7}	1.59×10^{7}	1.13×10^{7}	2.05×10^{7}	1.95×10^{7}	1.15×10^{7}	7.68×10^{6}	7.93×10^{6}	3.90×10^{6}	1.97×10^{6}	7.63×10^{6}	7.92×10^{6}	5.25×10^{6}	1.98×10^{6}
213	5-Ethylcyclopent-2-en-1-one	1.52×10^{6}	1.60×10^{6}	1.69×10^{6}	1.55×10^{6}	9.72×10^{5}	1.83×10^{6}	1.76×10^{6}	1.23×10^{6}	8.64×10^{5}	1.29×10^{6}	5.72×10^{5}	2.53×10^{5}	1.10×10^{6}	1.17×10^{6}	7.47×10^{5}	1.77×10^{5}
214	6-Methylenebicyclo[3.2.0]hept-3-en-2-one	1.42×10^{6}	1.54×10^{6}	1.31×10^{6}	1.24×10^{6}	7.24×10^{5}	1.66×10^{6}	1.20×10^{6}	1.00×10^{6}	1.08×10^{6}	1.66×10^{6}	6.77×10^{5}	4.39×10^{5}	1.16×10^{6}	1.20×10^{6}	6.96×10^{5}	3.01×10^{5}
215	3-Methyl-2-cyclohexen-1-one	1.39×10^{7}	1.56×10^{7}	1.66×10^{7}	1.47×10^{7}	1.13×10^{7}	2.07×10^{7}	1.82×10^{7}	1.18×10^{7}	1.17×10^{7}	1.81×10^{7}	9.49×10^{6}	4.14×10^{6}	1.68×10^{7}	1.92×10^{7}	1.34×10^{7}	4.07×10^{6}
216	2,2,6-Trimethylcyclohexanone	6.13×10^{5}	8.18×10^{5}	7.78×10^{5}	7.67×10^{5}	5.22×10^{5}	6.21×10^{5}	3.86×10^{5}	8.58×10^{5}	1.05×10^{6}	1.63×10^{6}	9.42×10^{5}	7.36×10^{5}	1.36×10^{6}	1.07×10^{6}	5.33×10^{5}	5.85×10^{5}
217	2-Cyclohexene-1,4-dione	1.97×10^{7}	2.96×10^{7}	3.44×10^{7}	3.38×10^{7}	3.88×10^{7}	3.28×10^{7}	3.97×10^{7}	2.34×10^{7}	1.76×10^{7}	1.75×10^{7}	7.65×10^{6}	3.88×10^{6}	1.55×10^{7}	1.42×10^{7}	1.13×10^{7}	3.65×10^{6}
218	3,5-Dimethyl-2-cyclohexen-1-one	3.48×10^{6}	4.30×10^{6}	6.56×10^{6}	6.45×10^{6}	5.31×10^{6}	8.27×10^{6}	6.59×10^{6}	5.50×10^{6}	2.95×10^{6}	3.69×10^{6}	2.75×10^{6}	1.14×10^{6}	4.87×10^{6}	4.90×10^{6}	2.87×10^{6}	1.21×10^{6}
219	2-Hydroxy-3-methyl-2-cyclopenten-1-one	7.82×10^{7}	6.90×10^{7}	8.04×10^{7}	9.10×10^{7}	1.15×10^{8}	9.79×10^{7}	9.18×10^{7}	6.54×10^{7}	4.47×10^{6}	6.60×10^{6}	2.28×10^{6}	1.93×10^{5}	5.62×10^{6}	6.42×10^{6}	1.84×10^{6}	2.32×10^{4}
220	2,3,4-Trimethyl-2-cyclopenten-1-one	3.23×10^{7}	3.83×10^{7}	3.92×10^{7}	3.73×10^{7}	3.43×10^{7}	4.78×10^{7}	4.33×10^{7}	3.12×10^{7}	1.93×10^{7}	2.76×10^{7}	1.39×10^{7}	5.87×10^{6}	2.47×10^{7}	2.67×10^{7}	1.67×10^{7}	6.13×10^{6}
221	3,5-Dimethyl-1,2-cyclopentanedione	2.47×10^{7}	2.19×10^{7}	2.32×10^{7}	2.52×10^{7}	2.33×10^{7}	2.88×10^{7}	2.86×10^{7}	2.10×10^{7}	4.74×10^{6}	4.33×10^{6}	2.44×10^{6}	4.39×10^{5}	3.62×10^{6}	4.51×10^{6}	2.24×10^{6}	6.87×10^{5}
222	3-Ethyl-2-hydroxy-2-cyclopenten-1-one	7.90×10^{7}	7.09×10^{7}	8.21×10^{7}	7.76×10^{7}	8.97×10^{7}	9.64×10^{7}	8.65×10^{7}	6.32×10^{7}	2.35×10^{7}	3.70×10^{7}	2.07×10^{7}	8.61×10^{6}	3.15×10^{7}	3.76×10^{7}	2.61×10^{7}	9.02×10^{6}
	Aromatics																
223	Acetophenone	1.63×10^{7}	1.68×10^{7}	1.65×10^{7}	1.82×10^{7}	1.72×10^{7}	1.56×10^{7}	1.60×10^{7}	1.55×10^{7}	1.90×10^{7}	2.23×10^{7}	1.23×10^{7}	4.73×10^{6}	1.78×10^{7}	1.73×10^{7}	9.95×10^{6}	5.06×10^{6}
224	1-Phenyl-2-propanone	9.22×10^{6}	9.11×10^{6}	9.00×10^{6}	1.07×10^{7}	9.27×10^{6}	7.89×10^{6}	7.46×10^{6}	9.60×10^{6}	8.88×10^{6}	8.73×10^{6}	4.08×10^{6}	2.25×10^{6}	7.63×10^{6}	6.87×10^{6}	3.70×10^{6}	2.35×10^{6}
225	*o*-Hydroxyacetophenone	5.66×10^{6}	5.21×10^{6}	4.89×10^{6}	5.35×10^{6}	6.22×10^{6}	7.48×10^{6}	6.53×10^{6}	5.72×10^{6}	8.16×10^{6}	8.03×10^{6}	4.60×10^{6}	2.00×10^{6}	7.64×10^{6}	7.70×10^{6}	4.86×10^{6}	2.13×10^{6}
226	1-Phenyl-1,2-propanedione	1.16×10^{6}	1.08×10^{6}	9.82×10^{5}	1.08×10^{6}	1.43×10^{6}	1.30×10^{6}	1.13×10^{6}	1.03×10^{6}	1.52×10^{6}	1.73×10^{6}	9.09×10^{5}	2.52×10^{5}	1.58×10^{6}	1.37×10^{6}	7.08×10^{5}	3.89×10^{5}
227	*p*-Methylacetophenone	2.87×10^{6}	3.57×10^{6}	2.87×10^{6}	3.52×10^{6}	3.54×10^{6}	4.19×10^{6}	5.00×10^{6}	2.64×10^{6}	6.48×10^{6}	7.31×10^{6}	3.38×10^{6}	1.63×10^{6}	6.49×10^{6}	6.63×10^{6}	3.58×10^{6}	1.28×10^{6}
228	1-Phenyl-2-butanone	1.58×10^{6}	1.54×10^{6}	1.12×10^{6}	1.80×10^{6}	1.18×10^{6}	1.20×10^{6}	1.08×10^{6}	1.39×10^{6}	2.83×10^{6}	3.03×10^{6}	1.24×10^{6}	7.85×10^{5}	2.15×10^{6}	1.83×10^{6}	9.21×10^{5}	5.82×10^{5}
229	1-(4-Hydroxyphenyl)-1-propanone	7.62×10^{5}	5.80×10^{5}	6.98×10^{5}	4.84×10^{5}	6.67×10^{5}	7.25×10^{5}	5.92×10^{5}	6.60×10^{5}	8.15×10^{5}	7.57×10^{5}	7.33×10^{5}	1.44×10^{5}	8.60×10^{5}	7.50×10^{5}	4.53×10^{5}	1.41×10^{5}
230	4-Hydroxy-3-methylacetophenone	5.82×10^{6}	5.92×10^{6}	5.38×10^{6}	7.75×10^{6}	7.56×10^{6}	8.76×10^{6}	7.34×10^{6}	5.84×10^{6}	5.96×10^{6}	6.49×10^{6}	3.21×10^{6}	1.35×10^{6}	6.04×10^{6}	5.98×10^{6}	3.46×10^{6}	1.64×10^{6}
	**Volatile phenols**	8.68×10^{8} (1.9)	6.66×10^{8} (12.8)	6.43×10^{8} (10.6)	8.01×10^{8} (12.7)	7.21×10^{8} (11.7)	5.78×10^{8} (12.5)	3.81×10^{8} (3.1)	6.79×10^{8} (17.6)	1.08×10^{9} (9.3)	9.01×10^{8} (10.8)	4.20×10^{8} (23.6)	2.29×10^{8} (24.9)	6.29×10^{8} (12.1)	4.64×10^{8} (9.2)	2.06×10^{8} (18.6)	2.35×10^{8} (13.3)
231	2-methoxyphenol (Guaiacol)	3.28×10^{8}	2.15×10^{8}	2.47×10^{8}	2.94×10^{8}	2.68×10^{8}	2.26×10^{8}	1.60×10^{8}	2.27×10^{8}	3.30×10^{8}	2.69×10^{8}	1.21×10^{8}	6.04×10^{7}	1.92×10^{8}	1.40×10^{8}	6.75×10^{7}	6.50×10^{7}
232	2,6-Dimethylphenol	2.71×10^{6}	2.08×10^{6}	2.67×10^{6}	2.38×10^{6}	2.74×10^{6}	3.34×10^{6}	3.43×10^{6}	2.57×10^{6}	5.27×10^{6}	5.64×10^{6}	2.87×10^{6}	9.77×10^{5}	6.23×10^{6}	5.87×10^{6}	2.97×10^{6}	9.78×10^{5}
233	2-Allylphenol	2.59×10^{7}	1.60×10^{7}	9.51×10^{6}	1.17×10^{7}	2.04×10^{7}	1.37×10^{7}	2.09×10^{7}	9.68×10^{6}	2.81×10^{7}	1.98×10^{7}	1.22×10^{7}	6.54×10^{6}	2.17×10^{7}	2.12×10^{7}	1.11×10^{7}	5.58×10^{6}
234	4-Ethyl-2-methoxyphenol (4-Ethylguaiacol)	1.38×10^{8}	9.95×10^{7}	7.55×10^{7}	1.22×10^{8}	8.86×10^{7}	6.00×10^{7}	3.18×10^{7}	1.02×10^{8}	2.04×10^{8}	1.60×10^{8}	7.13×10^{7}	3.98×10^{7}	1.03×10^{8}	6.79×10^{7}	2.50×10^{7}	4.30×10^{7}

235	2-Methoxy-4-vinylphenol (4-Vinylguaiacol)	3.72×10^{8}	3.33×10^{8}	3.08×10^{8}	3.71×10^{8}	3.42×10^{8}	2.75×10^{8}	1.66×10^{8}	3.37×10^{8}	5.08×10^{8}	4.46×10^{8}	2.13×10^{8}	1.21×10^{8}	3.06×10^{8}	2.29×10^{8}	9.96×10^{7}	1.20×10^{8}
	**Oxazoles**	5.16×10^{7} (3.7)	6.70×10^{7} (6.4)	6.36×10^{7} (6.3)	6.59×10^{7} (3.4)	3.56×10^{7} (45.8)	7.38×10^{7} (2.9)	4.89×10^{7} (15.9)	4.73×10^{7} (28.0)	5.31×10^{7} (11.6)	6.48×10^{7} (7.9)	3.10×10^{7} (39.7)	1.81×10^{7} (20.3)	5.31×10^{7} (5.0)	5.27×10^{7} (17.9)	3.14×10^{7} (21.5)	1.63×10^{7} (8.8)
236	4-Methyloxazole	2.33×10^{6}	2.61×10^{6}	2.84×10^{6}	1.77×10^{6}	5.16×10^{5}	2.91×10^{6}	2.03×10^{6}	1.71×10^{6}	2.23×10^{6}	1.73×10^{6}	1.38×10^{6}	8.49×10^{5}	1.31×10^{6}	1.56×10^{6}	1.66×10^{6}	9.57×10^{5}
237	4,5-Dimethyloxazole	3.49×10^{6}	6.08×10^{6}	6.86×10^{6}	4.97×10^{6}	1.56×10^{6}	5.87×10^{6}	3.74×10^{6}	2.73×10^{6}	8.44×10^{6}	8.56×10^{6}	3.43×10^{6}	2.31×10^{6}	6.46×10^{6}	6.25×10^{6}	3.57×10^{6}	2.09×10^{6}
238	Trimethyloxazole	1.83×10^{7}	2.88×10^{7}	2.78×10^{7}	2.63×10^{7}	1.05×10^{7}	2.88×10^{7}	2.11×10^{7}	1.63×10^{7}	1.38×10^{7}	2.32×10^{7}	9.71×10^{6}	7.00×10^{6}	1.97×10^{7}	2.09×10^{7}	1.25×10^{7}	7.35×10^{6}
239	4-Ethyl-2,5-dimethyloxazole	5.50×10^{6}	6.35×10^{6}	5.77×10^{6}	6.45×10^{6}	1.55×10^{6}	7.99×10^{6}	3.27×10^{6}	5.52×10^{6}	3.55×10^{6}	6.97×10^{6}	3.25×10^{6}	2.14×10^{6}	6.36×10^{6}	3.76×10^{6}	3.65×10^{6}	5.35×10^{5}
240	2-Ethyl-4-methyl-5-propyloxazole	1.44×10^{6}	2.48×10^{6}	1.93×10^{6}	2.32×10^{6}	1.15×10^{6}	1.96×10^{6}	1.69×10^{6}	1.82×10^{6}	1.75×10^{6}	2.56×10^{6}	1.70×10^{6}	8.73×10^{5}	2.44×10^{6}	2.31×10^{6}	1.26×10^{6}	8.21×10^{5}
241	4,5-Dimethyl-2-propyloxazole	3.89×10^{5}	3.88×10^{5}	2.21×10^{5}	3.10×10^{5}	1.56×10^{5}	2.68×10^{5}	2.91×10^{5}	3.16×10^{5}	8.38×10^{5}	6.55×10^{5}	6.57×10^{5}	3.99×10^{5}	6.37×10^{5}	7.31×10^{5}	4.19×10^{5}	3.61×10^{5}
242	4,5-Dimethyl-2-isobutyloxazole	3.60×10^{6}	4.79×10^{6}	4.33×10^{6}	5.68×10^{6}	3.42×10^{6}	4.30×10^{6}	3.43×10^{6}	5.10×10^{6}	4.10×10^{6}	5.59×10^{6}	3.27×10^{6}	2.04×10^{6}	4.19×10^{6}	4.42×10^{6}	2.40×10^{6}	1.53×10^{6}
243	Benzoxazole	1.14×10^{7}	1.06×10^{7}	1.02×10^{7}	1.34×10^{7}	1.23×10^{7}	1.14×10^{7}	9.31×10^{6}	9.41×10^{6}	6.08×10^{6}	4.47×10^{6}	1.93×10^{6}	1.16×10^{5}	2.69×10^{6}	2.97×10^{6}	4.29×10^{5}	1.80×10^{5}
244	2-Methylbenzoxazole	5.07×10^{6}	4.93×10^{6}	3.62×10^{6}	4.56×10^{6}	4.40×10^{6}	1.02×10^{7}	4.01×10^{6}	4.34×10^{6}	1.23×10^{7}	1.11×10^{7}	5.65×10^{6}	2.41×10^{6}	9.34×10^{6}	9.82×10^{6}	5.46×10^{6}	2.43×10^{6}
	**Pyrazines**	2.51×10^{9} (8.3)	2.57×10^{9} (20.5)	2.36×10^{9} (2.8)	2.45×10^{9} (9.1)	2.30×10^{9} (5.6)	2.02×10^{9} (8.6)	2.21×10^{9} (15.8)	2.67×10^{9} (15.0)	1.93×10^{9} (5.3)	2.10×10^{9} (10.8)	1.47×10^{9} (20.0)	9.26×10^{8} (5.6)	1.82×10^{9} (5.1)	1.78×10^{9} (9.1)	1.13×10^{9} (6.3)	1.03×10^{9} (9.1)
245	Pyrazine	1.14×10^{8}	1.43×10^{8}	1.32×10^{8}	1.28×10^{8}	6.96×10^{7}	1.25×10^{8}	1.04×10^{8}	1.16×10^{8}	6.31×10^{7}	5.74×10^{7}	2.13×10^{7}	1.20×10^{7}	4.27×10^{7}	3.81×10^{7}	2.09×10^{7}	1.06×10^{7}
246	Methylpyrazine	4.04×10^{8}	3.15×10^{8}	2.57×10^{8}	2.47×10^{8}	3.92×10^{8}	4.24×10^{7}	4.46×10^{8}	4.28×10^{8}	3.85×10^{8}	4.07×10^{8}	2.85×10^{8}	1.96×10^{8}	3.73×10^{8}	3.56×10^{8}	2.38×10^{8}	2.08×10^{8}
247	2,5-Dimethylpyrazine	3.18×10^{8}	4.58×10^{8}	3.80×10^{8}	1.28×10^{8}	3.33×10^{8}	2.44×10^{8}	1.55×10^{8}	3.88×10^{8}	3.46×10^{8}	2.84×10^{8}	2.88×10^{8}	2.15×10^{8}	3.86×10^{8}	3.46×10^{8}	2.62×10^{8}	2.05×10^{8}
248	2-Ethylpyrazine	1.22×10^{8}	1.04×10^{8}	9.79×10^{7}	9.75×10^{7}	1.03×10^{8}	1.04×10^{8}	1.05×10^{8}	8.42×10^{7}	2.17×10^{8}	1.54×10^{8}	1.49×10^{8}	9.55×10^{7}	1.41×10^{8}	1.60×10^{8}	9.27×10^{7}	8.51×10^{7}
249	2,3-Dimethylpyrazine	1.43×10^{8}	1.34×10^{8}	1.42×10^{8}	1.70×10^{8}	1.06×10^{8}	8.26×10^{7}	1.26×10^{8}	1.44×10^{8}	7.19×10^{7}	7.99×10^{7}	4.48×10^{7}	2.49×10^{7}	6.92×10^{7}	6.31×10^{7}	3.45×10^{7}	2.88×10^{7}
250	Vinylpyrazine	3.81×10^{7}	5.11×10^{7}	5.11×10^{7}	5.69×10^{7}	3.07×10^{7}	4.84×10^{7}	4.00×10^{7}	4.52×10^{7}	1.81×10^{7}	2.16×10^{7}	9.22×10^{6}	5.06×10^{6}	1.65×10^{7}	1.56×10^{7}	8.49×10^{6}	5.31×10^{6}
251	2-Isopropylpyrazine	2.76×10^{6}	3.59×10^{6}	3.52×10^{6}	3.73×10^{6}	1.66×10^{6}	2.89×10^{6}	2.49×10^{6}	3.12×10^{6}	1.40×10^{6}	2.60×10^{6}	1.39×10^{6}	5.78×10^{5}	2.09×10^{6}	1.99×10^{6}	1.03×10^{6}	7.66×10^{5}
252	2-Ethyl-6-methylpyrazine	3.05×10^{8}	1.93×10^{8}	2.33×10^{8}	2.84×10^{8}	2.10×10^{8}	3.12×10^{8}	2.15×10^{8}	2.31×10^{8}	2.73×10^{8}	3.74×10^{8}	2.50×10^{8}	1.10×10^{8}	2.36×10^{8}	2.29×10^{8}	1.82×10^{8}	1.62×10^{8}
253	2-Ethyl-3-methylpyrazine	1.43×10^{8}	2.32×10^{8}	1.92×10^{8}	2.73×10^{8}	1.91×10^{8}	1.74×10^{8}	2.57×10^{8}	2.69×10^{8}	1.60×10^{8}	1.86×10^{8}	1.51×10^{8}	1.20×10^{8}	1.61×10^{8}	1.99×10^{8}	8.57×10^{7}	1.30×10^{8}
254	2-Propylpyrazine	4.40×10^{7}	5.50×10^{7}	4.34×10^{7}	6.34×10^{7}	3.25×10^{7}	4.47×10^{7}	3.64×10^{7}	5.07×10^{7}	2.28×10^{7}	3.54×10^{7}	1.82×10^{7}	9.12×10^{6}	2.68×10^{7}	2.64×10^{7}	1.18×10^{7}	1.06×10^{7}
255	2-Vinyl-6-methylpyrazine	1.30×10^{8}	1.45×10^{8}	1.37×10^{8}	1.61×10^{8}	1.13×10^{8}	1.27×10^{8}	1.13×10^{8}	1.27×10^{8}	6.48×10^{7}	7.64×10^{7}	3.82×10^{7}	1.86×10^{7}	6.13×10^{7}	6.15×10^{7}	3.31×10^{7}	2.14×10^{7}
256	Acetylpyrazine	5.10×10^{7}	7.33×10^{7}	7.91×10^{7}	9.96×10^{7}	7.50×10^{7}	7.36×10^{7}	6.20×10^{7}	7.67×10^{7}	7.78×10^{6}	1.36×10^{7}	5.60×10^{6}	2.33×10^{6}	8.41×10^{6}	7.95×10^{6}	4.70×10^{6}	2.79×10^{6}
257	2-Methyl-3-isopropylpyrazine	1.84×10^{6}	3.72×10^{6}	3.76×10^{6}	4.14×10^{6}	2.85×10^{6}	3.70×10^{6}	3.92×10^{6}	3.92×10^{6}	6.55×10^{5}	3.10×10^{6}	1.63×10^{6}	6.33×10^{5}	1.97×10^{6}	2.18×10^{6}	1.62×10^{6}	7.97×10^{5}
258	Isobutylpyrazine	4.01×10^{6}	4.57×10^{6}	4.26×10^{6}	5.43×10^{6}	2.77×10^{6}	3.21×10^{6}	2.57×10^{6}	5.29×10^{6}	1.58×10^{6}	3.82×10^{6}	1.47×10^{6}	1.01×10^{6}	2.52×10^{6}	2.27×10^{6}	1.18×10^{6}	7.85×10^{5}
259	Isopropenylpyrazine	5.66×10^{7}	5.91×10^{7}	4.34×10^{7}	7.49×10^{7}	5.11×10^{7}	5.10×10^{7}	3.84×10^{7}	6.01×10^{7}	2.07×10^{7}	3.42×10^{7}	1.68×10^{7}	8.20×10^{6}	2.19×10^{7}	1.65×10^{7}	8.42×10^{6}	9.29×10^{6}
260	2,6-Diethylpyrazine	1.01×10^{8}	3.11×10^{7}	2.38×10^{7}	3.59×10^{7}	7.36×10^{7}	3.28×10^{7}	5.91×10^{7}	5.64×10^{7}	3.81×10^{7}	6.84×10^{7}	3.41×10^{7}	2.30×10^{7}	4.05×10^{7}	4.42×10^{7}	3.94×10^{7}	3.29×10^{7}
261	2-Isopropyl-3-methoxypyrazine	2.78×10^{4}	7.73×10^{5}	7.27×10^{5}	4.00×10^{5}	2.30×10^{5}	4.89×10^{5}	4.55×10^{5}	4.44×10^{5}	2.29×10^{4}	1.06×10^{6}	5.28×10^{5}	2.32×10^{5}	8.63×10^{5}	5.27×10^{5}	3.88×10^{5}	3.31×10^{5}

262	6,7-Dihydro-5*H*-cyclopentapyrazine	8.97×10^{7}	9.29×10^{7}	7.70×10^{7}	1.17×10^{8}	8.63×10^{7}	9.16×10^{7}	6.00×10^{7}	1.01×10^{8}	1.18×10^{7}	1.63×10^{7}	9.83×10^{6}	2.29×10^{6}	1.28×10^{7}	2.27×10^{7}	5.62×10^{6}	4.61×10^{6}
263	2-Acetyl-3-methylpyrazine	1.53×10^{8}	1.48×10^{8}	1.67×10^{8}	1.84×10^{8}	1.86×10^{8}	1.59×10^{8}	1.54×10^{8}	1.85×10^{8}	3.07×10^{7}	3.54×10^{7}	1.94×10^{7}	9.72×10^{6}	2.52×10^{7}	2.47×10^{7}	1.41×10^{7}	1.10×10^{7}
264	2-Isobutyl-3-methylpyrazine	1.71×10^{7}	2.01×10^{7}	1.82×10^{7}	2.52×10^{7}	1.34×10^{7}	1.42×10^{7}	1.06×10^{7}	2.60×10^{7}	1.25×10^{7}	1.85×10^{7}	8.92×10^{6}	5.21×10^{6}	1.12×10^{7}	1.04×10^{7}	4.59×10^{6}	6.36×10^{6}
265	5*H*-5-Methyl-6,7-dihydrocyclopentapyrazine	7.58×10^{7}	7.22×10^{7}	6.51×10^{7}	7.18×10^{7}	6.66×10^{7}	8.22×10^{7}	5.23×10^{7}	6.66×10^{7}	2.15×10^{7}	2.76×10^{7}	1.27×10^{7}	6.43×10^{6}	1.99×10^{7}	1.89×10^{7}	9.28×10^{6}	7.26×10^{6}
266	2-Methyl-6-(1-propenyl)pyrazine (isomer)	2.17×10^{6}	2.66×10^{6}	2.22×10^{6}	4.46×10^{6}	2.00×10^{6}	2.38×10^{6}	1.62×10^{6}	5.23×10^{6}	2.40×10^{6}	2.92×10^{6}	1.71×10^{6}	9.11×10^{5}	1.98×10^{6}	1.29×10^{6}	7.55×10^{5}	6.94×10^{5}
267	2,3-Diethyl-5-methylpyrazine	8.40×10^{7}	9.16×10^{7}	7.54×10^{7}	3.97×10^{7}	4.65×10^{7}	7.75×10^{7}	6.85×10^{7}	2.22×10^{7}	5.95×10^{7}	8.40×10^{7}	3.69×10^{7}	2.53×10^{7}	7.61×10^{7}	6.37×10^{7}	3.26×10^{7}	4.33×10^{7}
268	2-Methyl-6-(1-propenyl)pyrazine (isomer)	3.37×10^{7}	3.16×10^{7}	3.12×10^{7}	4.34×10^{7}	2.98×10^{7}	3.10×10^{7}	2.19×10^{7}	3.69×10^{7}	2.11×10^{7}	2.21×10^{7}	1.15×10^{7}	5.71×10^{6}	1.44×10^{7}	1.52×10^{7}	8.22×10^{6}	6.92×10^{6}
269	2-Acetyl-3-ethylpyrazine	1.44×10^{7}	1.46×10^{7}	1.71×10^{7}	2.09×10^{7}	1.37×10^{7}	1.48×10^{7}	1.05×10^{7}	1.95×10^{7}	1.41×10^{7}	1.64×10^{7}	1.21×10^{7}	3.63×10^{6}	1.28×10^{7}	1.06×10^{7}	4.83×10^{6}	7.01×10^{6}
270	2-Isoamylpyrazine	4.10×10^{6}	4.28×10^{6}	4.09×10^{6}	5.83×10^{6}	3.25×10^{6}	3.73×10^{6}	2.60×10^{6}	5.96×10^{6}	4.73×10^{6}	5.47×10^{6}	2.55×10^{6}	2.22×10^{6}	3.84×10^{6}	9.07×10^{5}	1.07×10^{6}	1.41×10^{6}
271	2-Isobutyl-3-methoxypyrazine	2.43×10^{6}	1.01×10^{7}	8.50×10^{6}	7.09×10^{6}	4.83×10^{6}	1.04×10^{7}	1.43×10^{7}	7.32×10^{6}	3.07×10^{6}	1.20×10^{7}	6.56×10^{6}	2.61×10^{6}	5.15×10^{6}	9.44×10^{6}	8.75×10^{6}	2.47×10^{6}
272	2-Butyl-3-methylpyrazine	1.25×10^{6}	1.30×10^{6}	9.24×10^{5}	1.53×10^{6}	1.02×10^{6}	1.06×10^{6}	7.68×10^{5}	1.38×10^{6}	1.34×10^{6}	1.30×10^{6}	6.84×10^{5}	3.22×10^{5}	9.53×10^{5}	7.12×10^{5}	3.53×10^{5}	3.65×10^{5}
273	2,5-Dimethyl-3-isobutylpyrazine	5.04×10^{6}	7.21×10^{6}	6.15×10^{6}	9.72×10^{6}	4.94×10^{6}	4.72×10^{6}	3.61×10^{6}	1.05×10^{7}	6.06×10^{6}	8.02×10^{6}	3.88×10^{6}	1.42×10^{6}	5.84×10^{6}	3.83×10^{6}	1.99×10^{6}	2.89×10^{6}
274	1-Methylpyrrolo(1,2-a)pyrazine	1.28×10^{7}	1.30×10^{7}	1.29×10^{7}	1.71×10^{7}	1.17×10^{7}	1.17×10^{7}	1.06×10^{7}	1.53×10^{7}	6.34×10^{6}	7.01×10^{6}	3.52×10^{6}	1.64×10^{6}	5.18×10^{6}	5.34×10^{6}	3.02×10^{6}	1.85×10^{6}
275	6,7-Dihydro-2,5-dimethyl-5*H*-cyclopentapyrazine	1.28×10^{7}	2.13×10^{7}	2.02×10^{7}	2.72×10^{7}	1.89×10^{7}	1.83×10^{7}	1.38×10^{7}	2.54×10^{7}	7.67×10^{6}	7.11×10^{6}	4.45×10^{6}	1.57×10^{6}	7.40×10^{6}	6.43×10^{6}	2.88×10^{6}	2.33×10^{6}
276	2,5-Diethyl-3,6-dimethylpyrazine	5.53×10^{6}	7.45×10^{6}	6.95×10^{6}	9.70×10^{6}	5.88×10^{6}	5.74×10^{6}	4.09×10^{6}	9.89×10^{6}	6.12×10^{6}	8.06×10^{6}	4.35×10^{6}	2.89×10^{6}	5.22×10^{6}	4.48×10^{6}	2.37×10^{6}	3.04×10^{6}
277	2-Methyl-6-isopentylpyrazine	1.44×10^{7}	1.53×10^{7}	1.39×10^{7}	2.25×10^{7}	1.13×10^{7}	1.10×10^{7}	8.44×10^{6}	2.35×10^{7}	1.83×10^{7}	1.83×10^{7}	1.07×10^{7}	7.31×10^{6}	1.14×10^{7}	9.66×10^{6}	5.20×10^{6}	7.20×10^{6}
278	2,6-Dimethyl-3(2-methyl-1-butyl)pyrazine	6.92×10^{5}	9.77×10^{5}	8.26×10^{5}	1.41×10^{6}	6.22×10^{5}	6.37×10^{5}	4.52×10^{5}	1.42×10^{6}	1.00×10^{6}	1.42×10^{6}	6.44×10^{5}	4.34×10^{5}	8.22×10^{5}	6.68×10^{5}	2.78×10^{5}	4.63×10^{5}
279	2,5-Dimethyl-3-isoamylpyrazine	5.30×10^{6}	8.34×10^{6}	6.87×10^{6}	1.20×10^{7}	5.74×10^{6}	5.40×10^{6}	4.29×10^{6}	1.19×10^{7}	7.15×10^{6}	6.67×10^{6}	3.50×10^{6}	3.88×10^{6}	4.77×10^{6}	3.05×10^{6}	1.69×10^{6}	3.45×10^{6}
280	2,3-Dimethyl-5-isopentylpyrazine	1.03×10^{6}	8.48×10^{5}	8.74×10^{5}	1.82×10^{6}	8.99×10^{5}	7.45×10^{5}	8.76×10^{5}	1.46×10^{6}	1.89×10^{6}	1.42×10^{6}	5.76×10^{5}	4.32×10^{5}	5.31×10^{5}	5.39×10^{5}	3.62×10^{5}	2.79×10^{5}
	**Pyridines**	5.75×10^{8} (22.9)	6.26×10^{8} (22.0)	6.17×10^{8} (36.1)	5.99×10^{8} (18.3)	4.77×10^{8} (41.2)	7.51×10^{8} (11.0)	4.19×10^{8} (44.4)	5.11×10^{8} (40.1)	6.57×10^{8} (17.1)	7.77×10^{8} (8.7)	3.31×10^{8} (46.8)	2.34×10^{8} (10.2)	6.12×10^{8} (16.2)	6.26×10^{8} (11.0)	4.23×10^{8} (20.0)	2.75×10^{8} (8.5)
281	Pyridine	4.33×10^{8}	4.72×10^{8}	4.70×10^{8}	4.32×10^{8}	3.48×10^{8}	5.82×10^{8}	2.78×10^{8}	3.84×10^{8}	6.25×10^{8}	7.43×10^{8}	3.17×10^{8}	2.25×10^{8}	5.79×10^{8}	6.02×10^{8}	4.10×10^{8}	2.68×10^{8}
282	2-Methylpyridine	2.85×10^{7}	3.08×10^{7}	3.02×10^{7}	2.93×10^{7}	1.69×10^{7}	3.27×10^{7}	2.37×10^{7}	1.94×10^{7}	5.27×10^{6}	2.14×10^{6}	9.72×10^{5}	7.89×10^{5}	2.05×10^{6}	1.80×10^{6}	7.29×10^{5}	5.24×10^{5}
283	2,6-Dimethylpyridine	3.83×10^{6}	4.63×10^{6}	4.93×10^{6}	5.25×10^{6}	2.96×10^{6}	6.54×10^{6}	5.11×10^{6}	4.22×10^{6}	n.d.	n.d.	n.d.	n.d.	n.d.	n.d.	n.d.	n.d.
284	2-Ethylpyridine	6.72×10^{6}	6.46×10^{6}	6.57×10^{6}	6.63×10^{6}	3.77×10^{6}	7.18×10^{6}	5.65×10^{6}	4.66×10^{6}	2.22×10^{6}	3.10×10^{6}	8.90×10^{5}	7.38×10^{5}	2.37×10^{6}	2.37×10^{6}	1.22×10^{6}	7.25×10^{5}
285	3-Ethylpyridine	5.15×10^{7}	5.54×10^{7}	4.42×10^{7}	5.77×10^{7}	4.32×10^{7}	5.67×10^{7}	5.05×10^{7}	4.51×10^{7}	7.30×10^{6}	7.96×10^{6}	1.99×10^{6}	2.69×10^{6}	1.24×10^{7}	5.60×10^{6}	1.47×10^{6}	1.54×10^{6}
286	3-Vinylpyridine	9.51×10^{5}	2.54×10^{6}	3.09×10^{6}	2.39×10^{6}	2.45×10^{6}	1.67×10^{6}	1.13×10^{6}	1.85×10^{6}	2.94×10^{6}	2.17×10^{6}	7.18×10^{5}	3.90×10^{5}	1.22×10^{6}	7.75×10^{5}	4.22×10^{4}	2.75×10^{5}
287	2-Acetylpyridine	4.20×10^{7}	3.98×10^{7}	4.34×10^{7}	4.88×10^{7}	4.23×10^{7}	4.93×10^{7}	4.33×10^{7}	3.85×10^{7}	1.07×10^{7}	1.46×10^{7}	7.99×10^{6}	3.43×10^{6}	1.20×10^{7}	1.06×10^{7}	7.69×10^{6}	3.59×10^{6}
288	Methyl 3-pyridinecarboxylate	8.19×10^{6}	1.32×10^{7}	1.29×10^{7}	1.51×10^{7}	1.58×10^{7}	1.36×10^{7}	1.04×10^{7}	1.23×10^{7}	2.13×10^{6}	3.59×10^{6}	1.28×10^{6}	2.98×10^{5}	2.11×10^{6}	2.30×10^{6}	1.46×10^{6}	5.38×10^{5}
289	2-Pentylpyridine	1.20×10^{6}	1.17×10^{6}	1.00×10^{6}	1.71×10^{6}	2.18×10^{6}	1.67×10^{6}	6.00×10^{5}	1.51×10^{6}	9.64×10^{5}	7.27×10^{5}	2.91×10^{5}	2.90×10^{5}	1.17×10^{6}	7.24×10^{5}	1.40×10^{5}	3.30×10^{5}

	**Pyrroles**	8.61×10^{8} (2.9)	8.63×10^{8} (5.4)	9.04×10^{8} (8.6)	9.02×10^{8} (8.8)	8.23×10^{8} (7.6)	9.96×10^{8} (4.2)	8.19×10^{8} (6.4)	7.23×10^{8} (29.5)	9.19×10^{8} (3.6)	9.81×10^{8} (8.4)	4.78×10^{8} (22.6)	3.28×10^{8} (17.9)	7.00×10^{8} (7.4)	7.19×10^{8} (12.5)	4.99×10^{8} (19.4)	2.93×10^{8} (5.6)
290	1-Methylpyrrole	4.38×10^{7}	6.33×10^{7}	6.77×10^{7}	4.10×10^{7}	1.24×10^{7}	5.81×10^{7}	3.78×10^{7}	2.91×10^{7}	1.21×10^{8}	2.12×10^{8}	1.04×10^{8}	8.48×10^{7}	1.33×10^{8}	1.31×10^{8}	1.07×10^{8}	5.87×10^{7}
291	Pyrrole	3.70×10^{7}	7.56×10^{7}	6.13×10^{7}	7.65×10^{7}	8.29×10^{6}	4.98×10^{7}	2.47×10^{7}	5.32×10^{7}	1.67×10^{8}	1.45×10^{8}	4.68×10^{7}	5.37×10^{7}	7.08×10^{7}	7.80×10^{7}	4.15×10^{7}	4.73×10^{7}
292	1-Ethylpyrrole	1.14×10^{7}	2.23×10^{7}	2.11×10^{7}	1.32×10^{7}	3.72×10^{6}	1.90×10^{7}	9.72×10^{6}	7.73×10^{6}	6.17×10^{7}	7.36×10^{7}	3.65×10^{7}	2.44×10^{7}	6.17×10^{7}	5.45×10^{7}	2.55×10^{7}	1.46×10^{7}
293	2,5-Dimethylpyrrole	7.71×10^{6}	1.93×10^{7}	1.59×10^{7}	1.20×10^{7}	1.44×10^{6}	1.36×10^{7}	5.53×10^{6}	6.16×10^{6}	3.30×10^{7}	4.73×10^{7}	1.33×10^{7}	1.31×10^{7}	2.44×10^{7}	2.27×10^{7}	1.57×10^{7}	7.86×10^{6}
294	2-Ethyl-4-methylpyrrole	4.42×10^{6}	5.56×10^{6}	3.99×10^{6}	3.63×10^{6}	9.66×10^{5}	3.15×10^{6}	3.74×10^{6}	2.81×10^{6}	6.49×10^{6}	1.28×10^{7}	5.20×10^{6}	4.38×10^{6}	8.99×10^{6}	4.51×10^{6}	4.39×10^{6}	2.78×10^{6}
295	1-Acetylpyrrole	3.35×10^{6}	3.52×10^{6}	2.93×10^{6}	3.26×10^{6}	2.49×10^{6}	2.64×10^{6}	2.87×10^{6}	3.05×10^{6}	3.12×10^{6}	3.43×10^{6}	1.75×10^{6}	8.66×10^{5}	2.80×10^{6}	2.61×10^{6}	1.49×10^{6}	1.01×10^{6}
296	3-Ethyl-2,4-dimethylpyrrole	1.35×10^{7}	1.60×10^{7}	1.49×10^{7}	1.43×10^{7}	5.52×10^{6}	1.31×10^{7}	9.47×10^{6}	1.28×10^{7}	1.35×10^{7}	2.32×10^{7}	1.17×10^{7}	8.62×10^{6}	1.62×10^{7}	1.53×10^{7}	8.31×10^{6}	6.23×10^{6}
297	1-Methyl-2-formylpyrrole	2.89×10^{8}	2.36×10^{8}	2.83×10^{8}	3.00×10^{8}	2.75×10^{8}	3.21×10^{8}	3.01×10^{8}	2.33×10^{8}	2.64×10^{8}	1.98×10^{8}	1.46×10^{8}	8.66×10^{7}	1.89×10^{8}	1.89×10^{8}	1.56×10^{8}	9.13×10^{7}
298	1-Ethyl-2-formylpyrrole	8.15×10^{7}	7.12×10^{7}	5.97×10^{7}	7.21×10^{7}	4.66×10^{7}	7.79×10^{7}	5.96×10^{7}	5.37×10^{7}	8.16×10^{7}	7.45×10^{7}	2.61×10^{7}	1.42×10^{7}	4.70×10^{7}	6.00×10^{7}	3.46×10^{7}	1.78×10^{7}
299	2-Acetyl-1-methylpyrrole	1.60×10^{8}	1.53×10^{8}	1.57×10^{8}	1.31×10^{8}	1.60×10^{8}	1.93×10^{8}	1.77×10^{8}	1.21×10^{8}	1.32×10^{8}	1.56×10^{8}	7.51×10^{7}	3.18×10^{7}	1.23×10^{8}	1.37×10^{8}	8.62×10^{7}	3.53×10^{7}
300	2-Acetylpyrrole	2.09×10^{8}	1.97×10^{8}	2.17×10^{8}	2.36×10^{8}	3.07×10^{8}	2.45×10^{8}	1.88×10^{8}	2.01×10^{8}	3.61×10^{7}	3.55×10^{7}	1.17×10^{7}	5.29×10^{6}	2.29×10^{7}	2.40×10^{7}	1.83×10^{7}	1.01×10^{7}
	**Sulfur compounds**	1.46×10^{8} (17.7)	1.65×10^{8} (34.9)	1.62×10^{8} (32.2)	1.25×10^{8} (3.1)	5.11×10^{7} (17.6)	1.90×10^{8} (50.1)	1.08×10^{8} (34.0)	9.87×10^{7} (16.4)	3.76×10^{8} (6.7)	4.42×10^{8} (9.2)	2.07×10^{8} (9.9)	1.99×10^{8} (38.7)	3.33×10^{8} (34.2)	3.32×10^{8} (30.9)	2.13×10^{8} (19.1)	1.35×10^{8} (7.0)
301	Dimethyl disulfide	8.61×10^{6}	1.60×10^{7}	1.91×10^{7}	6.47×10^{6}	1.54×10^{6}	1.63×10^{7}	6.92×10^{6}	4.77×10^{6}	6.03×10^{7}	6.61×10^{7}	3.01×10^{7}	3.17×10^{7}	4.92×10^{7}	4.77×10^{7}	2.30×10^{7}	1.83×10^{7}
302	Methylthio-2-propanone	4.78×10^{5}	1.06×10^{6}	1.27×10^{6}	1.04×10^{6}	3.29×10^{4}	1.22×10^{6}	4.76×10^{5}	3.51×10^{5}	1.42×10^{6}	9.60×10^{5}	4.27×10^{5}	3.58×10^{5}	7.28×10^{5}	7.33×10^{5}	3.71×10^{5}	2.08×10^{5}
303	2-Furylmethylsulfide	1.87×10^{5}	3.36×10^{5}	5.64×10^{5}	2.94×10^{5}	1.59×10^{4}	3.21×10^{5}	2.54×10^{5}	1.56×10^{5}	1.24×10^{6}	1.81×10^{6}	7.28×10^{5}	4.75×10^{5}	1.13×10^{6}	1.11×10^{6}	5.04×10^{5}	3.14×10^{5}
304	2-Furfurylthiol	2.16×10^{5}	1.38×10^{6}	1.57×10^{6}	9.52×10^{5}	1.33×10^{5}	6.76×10^{5}	1.72×10^{5}	1.59×10^{5}	5.69×10^{6}	1.08×10^{7}	1.93×10^{6}	2.77×10^{6}	3.86×10^{6}	5.67×10^{6}	3.01×10^{6}	1.08×10^{6}
305	3-(Methylthio)propanal (Methional)	1.66×10^{5}	6.88×10^{5}	7.37×10^{5}	7.46×10^{5}	7.13×10^{3}	8.24×10^{5}	1.74×10^{5}	1.04×10^{5}	8.45×10^{5}	8.50×10^{5}	1.73×10^{5}	2.33×10^{5}	6.25×10^{5}	7.24×10^{5}	4.40×10^{5}	1.55×10^{5}
306	1-(Methylthio)-2-butanone	2.16×10^{5}	6.68×10^{5}	7.58×10^{5}	6.99×10^{5}	7.57×10^{4}	6.50×10^{5}	2.69×10^{5}	1.08×10^{6}	8.47×10^{5}	8.51×10^{5}	2.78×10^{5}	2.33×10^{5}	6.11×10^{5}	5.98×10^{5}	2.50×10^{5}	2.11×10^{5}
307	Dimethyl trisulfide	1.00×10^{6}	4.22×10^{6}	3.71×10^{6}	6.97×10^{5}	4.27×10^{6}	3.60×10^{6}	8.68×10^{6}	7.34×10^{5}	1.52×10^{7}	3.69×10^{7}	1.79×10^{7}	8.47×10^{6}	2.40×10^{7}	2.01×10^{7}	8.32×10^{6}	5.66×10^{6}
308	3-Mercapto-3-methyl-1-butanol	2.16×10^{6}	7.34×10^{6}	8.87×10^{6}	7.04×10^{6}	1.52×10^{6}	1.39×10^{7}	4.84×10^{6}	2.13×10^{6}	4.49×10^{6}	4.90×10^{6}	9.67×10^{5}	7.87×10^{5}	3.46×10^{6}	5.06×10^{6}	2.61×10^{6}	4.40×10^{5}
309	2-Furfuryl methyl sulfide	9.20×10^{7}	1.06×10^{8}	9.55×10^{7}	7.41×10^{7}	2.07×10^{7}	1.17×10^{8}	6.11×10^{7}	6.12×10^{7}	2.00×10^{8}	2.48×10^{8}	1.23×10^{8}	1.29×10^{8}	2.00×10^{8}	1.96×10^{8}	1.45×10^{8}	9.23×10^{7}
310	3-Mercapto-3-methylbutyl formate	2.13×10^{6}	3.29×10^{6}	3.86×10^{6}	3.12×10^{6}	1.95×10^{6}	5.70×10^{6}	3.47×10^{6}	1.44×10^{6}	5.34×10^{6}	7.01×10^{6}	2.84×10^{6}	1.88×10^{6}	6.11×10^{6}	9.42×10^{6}	5.38×10^{6}	1.47×10^{6}
311	3-Mercapto-3-methylbutyl acetate	3.86×10^{6}	1.53×10^{6}	4.81×10^{6}	4.49×10^{6}	2.74×10^{6}	7.96×10^{6}	3.23×10^{6}	4.57×10^{6}	1.10×10^{7}	9.89×10^{6}	2.23×10^{6}	1.93×10^{6}	9.22×10^{6}	9.03×10^{6}	6.54×10^{6}	1.15×10^{6}
312	Furfuryl methyl disulfide	3.54×10^{7}	2.31×10^{7}	2.13×10^{7}	2.51×10^{7}	1.81×10^{7}	2.23×10^{7}	1.83×10^{7}	2.19×10^{7}	6.88×10^{7}	5.46×10^{7}	2.60×10^{7}	2.06×10^{7}	3.39×10^{7}	3.55×10^{7}	1.79×10^{7}	1.38×10^{7}
	**Terpenic compounds**	8.77×10^{7} (4.0)	1.88×10^{8} (1.5)	2.49×10^{8} (3.5)	2.02×10^{8} (5.9)	1.87×10^{8} (19.7)	3.42×10^{8} (2.0)	3.18×10^{8} (3.2)	1.64×10^{8} (23.9)	2.83×10^{7} (17.1)	7.68×10^{7} (20.9)	7.41×10^{7} (22.7)	3.26×10^{7} (22.0)	9.97×10^{7} (13.7)	1.19×10^{8} (17.0)	8.86×10^{7} (23.3)	2.63×10^{7} (27.7)
	Monoterpenes																
313	α-Pinene	3.65×10^{6}	5.82×10^{6}	4.40×10^{6}	4.23×10^{6}	4.05×10^{6}	7.60×10^{6}	6.29×10^{6}	2.78×10^{6}	3.83×10^{5}	7.88×10^{5}	6.39×10^{5}	3.19×10^{5}	7.66×10^{5}	8.92×10^{5}	4.36×10^{5}	1.77×10^{5}

314	β-Pinene	1.87×10^{6}	2.23×10^{6}	9.72×10^{5}	1.14×10^{6}	1.51×10^{6}	2.09×10^{6}	1.44×10^{6}	9.36×10^{5}	2.51×10^{5}	4.92×10^{5}	3.31×10^{5}	3.01×10^{5}	7.31×10^{5}	9.21×10^{5}	5.59×10^{5}	1.76×10^{5}
315	2,6-Dimethyl-2,6-octadiene (isomer)	9.35×10^{5}	5.46×10^{6}	7.16×10^{6}	3.99×10^{6}	2.81×10^{6}	9.56×10^{6}	9.14×10^{6}	3.49×10^{6}	5.32×10^{4}	5.80×10^{5}	4.99×10^{5}	1.90×10^{5}	6.69×10^{5}	8.92×10^{5}	5.36×10^{5}	1.06×10^{5}
316	β-Myrcene	4.02×10^{6}	2.13×10^{7}	2.83×10^{7}	1.91×10^{7}	1.38×10^{7}	4.19×10^{7}	3.75×10^{7}	1.48×10^{7}	2.35×10^{5}	2.95×10^{6}	2.15×10^{6}	1.24×10^{6}	3.95×10^{6}	4.92×10^{6}	3.17×10^{6}	8.27×10^{5}
317	2,6-Dimethyl-2,6-octadiene (isomer)	1.66×10^{6}	1.13×10^{7}	1.53×10^{7}	9.34×10^{6}	6.85×10^{6}	2.09×10^{7}	1.91×10^{7}	7.61×10^{6}	3.16×10^{4}	5.37×10^{5}	1.00×10^{6}	4.32×10^{5}	1.39×10^{6}	2.14×10^{6}	1.28×10^{6}	3.02×10^{5}
318	α-Phellandrene	4.75×10^{5}	1.62×10^{6}	1.90×10^{6}	2.98×10^{6}	1.27×10^{6}	3.07×10^{6}	2.57×10^{6}	1.32×10^{6}	1.67×10^{5}	1.15×10^{5}	1.61×10^{5}	1.82×10^{5}	3.45×10^{5}	4.82×10^{5}	3.07×10^{5}	7.23×10^{4}
319	α-Terpinene	9.37×10^{5}	2.91×10^{6}	3.66×10^{6}	3.72×10^{6}	1.59×10^{6}	4.83×10^{6}	4.83×10^{6}	2.33×10^{6}	1.36×10^{5}	4.91×10^{5}	4.82×10^{5}	2.05×10^{5}	6.42×10^{5}	6.36×10^{5}	4.48×10^{5}	7.51×10^{4}
320	*p*-Cymene	3.98×10^{6}	7.02×10^{6}	6.20×10^{6}	7.79×10^{6}	3.59×10^{6}	4.40×10^{6}	2.80×10^{6}	7.41×10^{6}	1.20×10^{6}	2.53×10^{6}	1.69×10^{6}	1.63×10^{6}	3.11×10^{6}	2.34×10^{6}	1.51×10^{6}	1.46×10^{6}
321	Limonene	2.10×10^{7}	3.02×10^{7}	3.56×10^{7}	3.06×10^{7}	2.59×10^{7}	4.16×10^{7}	3.93×10^{7}	2.48×10^{7}	2.16×10^{6}	4.70×10^{6}	7.56×10^{6}	6.02×10^{6}	1.30×10^{7}	1.40×10^{7}	1.12×10^{7}	6.06×10^{6}
322	β-Ocimene (isomer)	1.33×10^{6}	2.73×10^{6}	3.42×10^{6}	3.16×10^{6}	2.96×10^{6}	4.22×10^{6}	4.75×10^{6}	2.45×10^{6}	6.97×10^{4}	3.85×10^{5}	3.00×10^{5}	1.41×10^{5}	4.63×10^{5}	5.07×10^{5}	3.93×10^{5}	7.84×10^{4}
323	β-Ocimene (isomer)	3.52×10^{6}	1.52×10^{7}	2.02×10^{7}	1.37×10^{7}	1.46×10^{7}	2.84×10^{7}	2.76×10^{7}	1.18×10^{7}	3.97×10^{5}	2.55×10^{6}	2.18×10^{6}	9.79×10^{5}	3.21×10^{6}	3.77×10^{6}	2.60×10^{6}	6.80×10^{5}
324	γ-Terpinene	8.63×10^{5}	2.35×10^{6}	2.94×10^{6}	2.35×10^{6}	2.17×10^{6}	3.86×10^{6}	3.79×10^{6}	2.07×10^{6}	7.37×10^{4}	3.11×10^{5}	4.45×10^{5}	2.83×10^{5}	8.63×10^{5}	9.93×10^{5}	7.29×10^{5}	3.17×10^{5}
325	α-Terpinolene	2.69×10^{6}	6.84×10^{6}	9.12×10^{6}	6.83×10^{6}	6.97×10^{6}	1.41×10^{7}	1.39×10^{7}	6.23×10^{6}	6.29×10^{5}	1.91×10^{6}	1.57×10^{6}	7.15×10^{5}	2.14×10^{6}	2.74×10^{6}	1.92×10^{6}	5.22×10^{5}
326	*p*-Cymenene	5.63×10^{6}	7.71×10^{6}	6.20×10^{6}	7.87×10^{6}	6.04×10^{6}	6.93×10^{6}	1.91×10^{6}	6.66×10^{6}	3.00×10^{6}	3.46×10^{6}	4.19×10^{6}	1.50×10^{6}	3.40×10^{5}	2.92×10^{6}	1.66×10^{6}	1.27×10^{6}
327	Cosmene	6.05×10^{5}	1.05×10^{6}	1.63×10^{6}	1.35×10^{6}	1.34×10^{6}	1.18×10^{6}	1.19×10^{6}	1.43×10^{6}	1.37×10^{5}	5.46×10^{5}	2.48×10^{5}	2.22×10^{5}	5.24×10^{5}	3.17×10^{5}	2.21×10^{5}	1.52×10^{5}
328	*Allo*-ocimene	8.44×10^{5}	3.99×10^{6}	4.75×10^{6}	3.33×10^{6}	3.87×10^{6}	8.41×10^{6}	7.05×10^{6}	3.35×10^{6}	2.93×10^{5}	6.81×10^{5}	6.66×10^{5}	3.05×10^{5}	1.03×10^{6}	1.25×10^{6}	8.88×10^{5}	2.36×10^{5}
	Monoterpenoids																
329	Linalool oxide (isomer)	1.28×10^{7}	2.39×10^{7}	3.56×10^{7}	2.48×10^{7}	3.04×10^{7}	5.22×10^{7}	4.91×10^{7}	2.35×10^{7}	4.68×10^{6}	1.54×10^{7}	1.22×10^{7}	3.54×10^{6}	1.44×10^{7}	2.12×10^{7}	1.59×10^{7}	4.01×10^{6}
330	Linalool oxide (isomer)	7.23×10^{6}	1.44×10^{7}	2.37×10^{7}	2.93×10^{7}	1.76×10^{7}	3.23×10^{7}	3.06×10^{7}	1.39×10^{7}	2.13×10^{6}	4.79×10^{6}	6.67×10^{6}	2.40×10^{6}	4.82×10^{6}	8.04×10^{6}	7.72×10^{6}	4.89×10^{6}
331	Linalool	5.38×10^{6}	1.20×10^{7}	2.52×10^{7}	1.46×10^{7}	2.52×10^{7}	3.37×10^{7}	3.61×10^{7}	1.60×10^{7}	1.31×10^{6}	1.96×10^{7}	1.97×10^{7}	7.43×10^{6}	2.57×10^{7}	3.12×10^{7}	2.42×10^{7}	1.55×10^{5}
332	α-Terpineol	1.20×10^{6}	1.55×10^{6}	2.47×10^{6}	2.40×10^{6}	3.84×10^{6}	4.63×10^{6}	4.84×10^{6}	2.10×10^{6}	2.54×10^{6}	2.97×10^{6}	3.32×10^{6}	9.96×10^{5}	1.04×10^{7}	4.98×10^{6}	3.76×10^{6}	8.75×10^{5}
333	Safranal	7.60×10^{5}	8.93×10^{5}	6.50×10^{5}	5.60×10^{5}	5.66×10^{5}	5.64×10^{5}	6.73×10^{5}	1.08×10^{6}	1.42×10^{6}	1.33×10^{6}	6.57×10^{5}	3.90×10^{5}	9.61×10^{5}	1.03×10^{6}	6.41×10^{5}	3.15×10^{5}
334	*p*-Menth-1-en-9-al	3.69×10^{6}	4.96×10^{6}	6.51×10^{6}	5.44×10^{6}	6.90×10^{6}	1.02×10^{7}	1.13×10^{7}	5.04×10^{6}	6.10×10^{6}	7.37×10^{6}	6.03×10^{6}	2.86×10^{6}	9.05×10^{6}	1.21×10^{7}	8.07×10^{6}	2.10×10^{6}
	Sesquiterpenes																
335	α-Cubebene	6.73×10^{4}	6.38×10^{4}	6.61×10^{4}	1.33×10^{5}	7.09×10^{4}	1.00×10^{5}	4.27×10^{4}	8.70×10^{4}	9.76×10^{4}	1.89×10^{5}	1.11×10^{5}	3.50×10^{4}	1.61×10^{5}	1.08×10^{5}	7.22×10^{4}	2.47×10^{5}
336	α-Copaene	1.08×10^{6}	1.36×10^{6}	1.50×10^{6}	1.66×10^{6}	1.50×10^{6}	1.60×10^{6}	7.80×10^{5}	1.53×10^{6}	n.d.	n.d.	n.d.	n.d.	n.d.	n.d.	n.d.	n.d.
337	Longifolene	2.03×10^{5}	2.51×10^{5}	3.09×10^{5}	1.59×10^{5}	3.32×10^{5}	1.67×10^{6}	3.73×10^{5}	2.78×10^{5}	2.14×10^{5}	1.00×10^{6}	6.66×10^{5}	1.17×10^{5}	5.85×10^{5}	5.35×10^{5}	2.04×10^{5}	3.54×10^{5}
338	β-Caryophyllene	3.46×10^{5}	1.90×10^{5}	5.68×10^{5}	2.48×10^{5}	2.98×10^{5}	2.02×10^{6}	3.73×10^{5}	1.50×10^{5}	1.62×10^{5}	8.68×10^{5}	2.17×10^{5}	5.30×10^{4}	2.09×10^{5}	3.00×10^{5}	1.55×10^{5}	2.00×10^{5}
339	α-Humulene	2.18×10^{5}	2.15×10^{5}	1.97×10^{5}	2.01×10^{5}	2.03×10^{5}	2.15×10^{5}	1.91×10^{5}	2.13×10^{5}	1.33×10^{5}	1.12×10^{5}	1.29×10^{5}	4.04×10^{4}	1.16×10^{5}	7.72×10^{4}	3.99×10^{4}	4.31×10^{5}
340	α-Muurolene	9.60×10^{4}	7.01×10^{4}	6.34×10^{4}	5.27×10^{4}	5.50×10^{4}	6.61×10^{4}	3.63×10^{4}	6.23×10^{4}	9.45×10^{4}	4.92×10^{4}	6.46×10^{4}	9.26×10^{3}	5.90×10^{4}	4.53×10^{4}	6.27×10^{4}	4.69×10^{4}

341	δ-Cadinene	5.69×10^{5}	7.85×10^{5}	5.83×10^{5}	1.03×10^{6}	4.64×10^{5}	2.00×10^{5}	5.79×10^{4}	8.85×10^{5}	1.92×10^{5}	9.36×10^{4}	1.10×10^{5}	9.41×10^{4}	9.30×10^{4}	2.15×10^{4}	1.00×10^{4}	1.47×10^{5}
	**Norisoprenoids**	9.90×10^{6} (10.2)	1.54×10^{7} (22.0)	1.85×10^{7} (15.2)	1.75×10^{7} (7.5)	1.56×10^{7} (4.2)	1.30×10^{7} (3.7)	1.34×10^{7} (9.8)	1.68×10^{7} (24.0)	1.52×10^{7} (8.1)	1.69×10^{7} (19.3)	9.36×10^{6} (20.1)	6.31×10^{6} (30.5)	1.23×10^{7} (7.0)	9.65×10^{6} (6.1)	5.88×10^{6} (10.6)	5.44×10^{6} (17.0)
342	Vitispirane (C_{13})	6.76×10^{5}	1.49×10^{6}	1.50×10^{6}	1.78×10^{6}	8.84×10^{5}	1.03×10^{6}	9.48×10^{5}	1.79×10^{6}	6.05×10^{5}	7.18×10^{5}	5.09×10^{5}	3.72×10^{5}	8.66×10^{5}	3.33×10^{5}	9.93×10^{5}	3.84×10^{5}
343	Theaspirane (C_{13})	1.55×10^{6}	2.52×10^{6}	1.92×10^{6}	2.94×10^{6}	1.59×10^{6}	1.19×10^{6}	5.70×10^{5}	3.34×10^{6}	3.17×10^{6}	2.54×10^{6}	1.17×10^{6}	1.10×10^{6}	1.40×10^{6}	1.06×10^{6}	2.66×10^{5}	1.05×10^{6}
344	1,2-Dihydro-1,1,6-trimethylnaphthalene (TDN) (C_{13})	6.50×10^{5}	9.07×10^{5}	8.86×10^{5}	1.07×10^{6}	8.62×10^{5}	9.82×10^{5}	9.76×10^{5}	1.13×10^{6}	5.78×10^{5}	6.28×10^{5}	3.89×10^{5}	2.01×10^{5}	4.38×10^{5}	4.31×10^{5}	2.53×10^{5}	1.70×10^{5}
345	β-Damascenone (C_{13})	5.83×10^{6}	7.89×10^{6}	7.13×10^{6}	9.25×10^{6}	7.39×10^{6}	6.37×10^{6}	5.52×10^{6}	8.36×10^{6}	9.44×10^{6}	1.12×10^{7}	5.80×10^{6}	3.96×10^{6}	7.93×10^{6}	6.28×10^{6}	3.63×10^{6}	3.40×10^{6}
346	α-Ionone (C_{13})	6.74×10^{4}	7.66×10^{4}	4.71×10^{4}	8.34×10^{4}	5.40×10^{4}	6.66×10^{4}	6.31×10^{4}	7.39×10^{4}	1.14×10^{5}	1.19×10^{5}	4.66×10^{4}	3.55×10^{4}	5.25×10^{4}	5.65×10^{4}	3.78×10^{4}	2.46×10^{4}
347	Geranyl acetone (C_{13})	1.13×10^{6}	2.51×10^{6}	7.05×10^{6}	2.42×10^{6}	4.78×10^{6}	3.34×10^{6}	5.28×10^{6}	2.12×10^{6}	1.30×10^{6}	1.65×10^{6}	1.45×10^{6}	6.45×10^{5}	1.56×10^{6}	1.50×10^{6}	6.96×10^{5}	4.13×10^{5}
	**Thiazoles**	1.14×10^{8} (9.7)	1.64×10^{8} (26.2)	1.32×10^{8} (5.4)	1.79×10^{8} (4.6)	8.22×10^{7} (34.0)	1.13×10^{8} (1.4)	9.31×10^{7} (10.3)	1.30×10^{8} (31.9)	1.17×10^{8} (3.8)	1.57×10^{8} (11.2)	7.17×10^{7} (32.0)	3.98×10^{7} (15.9)	1.13×10^{8} (7.1)	1.09×10^{8} (18.3)	5.10×10^{7} (17.3)	4.25×10^{7} (8.2)
348	Thiazole	7.51×10^{6}	9.78×10^{6}	9.78×10^{6}	9.89×10^{6}	3.69×10^{6}	7.95×10^{6}	6.17×10^{6}	7.77×10^{6}	8.42×10^{6}	5.62×10^{6}	4.23×10^{6}	2.07×10^{6}	6.44×10^{6}	5.79×10^{6}	3.39×10^{6}	2.15×10^{6}
349	2-Methylthiazole	2.08×10^{6}	2.82×10^{6}	2.94×10^{6}	2.97×10^{6}	1.01×10^{6}	2.40×10^{6}	1.63×10^{6}	2.01×10^{6}	3.28×10^{6}	3.26×10^{6}	1.45×10^{6}	1.08×10^{6}	2.90×10^{6}	2.88×10^{6}	1.22×10^{6}	1.00×10^{6}
350	4-Methylthiazole	1.64×10^{7}	2.89×10^{7}	2.80×10^{7}	2.95×10^{7}	1.03×10^{7}	2.18×10^{7}	1.86×10^{7}	2.03×10^{7}	2.43×10^{7}	3.39×10^{7}	1.49×10^{7}	8.07×10^{6}	2.47×10^{7}	2.10×10^{7}	1.07×10^{7}	9.48×10^{6}
351	5-Methylthiazole	2.02×10^{5}	6.96×10^{5}	7.02×10^{5}	7.54×10^{5}	1.21×10^{5}	3.03×10^{5}	2.22×10^{5}	3.31×10^{5}	1.27×10^{6}	1.76×10^{6}	5.67×10^{5}	4.24×10^{5}	9.52×10^{5}	7.96×10^{5}	2.54×10^{5}	4.31×10^{5}
352	2,4-Dimethylthiazole	8.57×10^{6}	1.21×10^{7}	1.08×10^{7}	1.23×10^{7}	3.74×10^{6}	7.95×10^{6}	5.87×10^{6}	9.29×10^{6}	8.31×10^{6}	1.41×10^{7}	5.93×10^{6}	3.34×10^{6}	9.51×10^{6}	8.27×10^{6}	3.37×10^{6}	3.65×10^{6}
353	2,5-Dimethylthiazole	4.50×10^{6}	7.61×10^{6}	4.68×10^{6}	5.76×10^{6}	3.91×10^{6}	5.29×10^{6}	3.07×10^{6}	5.68×10^{6}	4.86×10^{6}	6.79×10^{6}	3.06×10^{6}	1.88×10^{6}	4.64×10^{6}	5.60×10^{6}	1.97×10^{6}	1.59×10^{6}
354	4,5-Dimethylthiazole	5.20×10^{6}	6.17×10^{6}	6.00×10^{6}	6.87×10^{6}	2.92×10^{6}	4.55×10^{6}	3.58×10^{6}	5.79×10^{6}	5.67×10^{6}	8.53×10^{6}	3.78×10^{6}	1.92×10^{6}	5.73×10^{6}	5.33×10^{6}	2.23×10^{6}	2.00×10^{6}
355	5-Ethylthiazole	3.07×10^{6}	3.70×10^{6}	3.81×10^{6}	4.30×10^{6}	2.06×10^{6}	2.94×10^{6}	2.41×10^{6}	3.39×10^{6}	5.14×10^{6}	6.54×10^{6}	3.16×10^{6}	1.68×10^{6}	4.79×10^{6}	4.41×10^{6}	2.15×10^{6}	1.75×10^{6}
356	2-Ethyl-4-methylthiazole	3.92×10^{6}	4.52×10^{6}	3.77×10^{6}	5.35×10^{6}	2.27×10^{6}	3.32×10^{6}	2.58×10^{6}	4.38×10^{6}	2.81×10^{6}	6.11×10^{6}	2.22×10^{6}	1.68×10^{6}	3.88×10^{6}	2.64×10^{6}	1.24×10^{6}	1.82×10^{6}
357	4-Propylthiazole	2.03×10^{6}	9.22×10^{5}	2.43×10^{5}	5.61×10^{5}	1.03×10^{5}	5.84×10^{5}	3.61×10^{5}	6.09×10^{5}	6.41×10^{5}	6.17×10^{5}	4.14×10^{5}	1.98×10^{5}	6.23×10^{5}	5.11×10^{5}	1.93×10^{5}	2.30×10^{5}
358	5-Ethyl-2-methylthiazole	4.11×10^{6}	5.54×10^{6}	2.54×10^{6}	1.06×10^{7}	2.10×10^{6}	3.04×10^{6}	1.33×10^{6}	3.91×10^{6}	4.43×10^{6}	6.94×10^{6}	2.95×10^{6}	2.17×10^{6}	3.90×10^{6}	3.81×10^{6}	1.82×10^{6}	1.99×10^{6}
359	2-Isopropyl-4-methylthiazole	9.86×10^{5}	1.30×10^{6}	1.10×10^{6}	1.66×10^{6}	5.14×10^{5}	6.09×10^{5}	5.05×10^{5}	1.74×10^{6}	5.67×10^{5}	1.20×10^{6}	6.87×10^{5}	3.07×10^{5}	7.94×10^{5}	6.57×10^{5}	2.55×10^{5}	4.19×10^{5}
360	5-Ethyl-4-methylthiazole	6.42×10^{6}	5.01×10^{6}	1.30×10^{6}	1.36×10^{7}	3.22×10^{6}	1.44×10^{6}	2.58×10^{6}	6.63×10^{5}	9.88×10^{6}	1.34×10^{7}	5.08×10^{6}	3.36×10^{6}	8.99×10^{6}	1.26×10^{7}	4.60×10^{6}	3.71×10^{6}
361	2-Acetylthiazole	1.91×10^{7}	2.02×10^{7}	2.02×10^{7}	2.35×10^{7}	1.59×10^{7}	1.93×10^{7}	1.83×10^{7}	1.87×10^{7}	1.54×10^{7}	1.77×10^{7}	8.33×10^{6}	3.87×10^{6}	1.35×10^{7}	1.36×10^{7}	7.93×10^{6}	4.29×10^{6}
362	4-Ethyl-2,5-dimethylthiazole	7.09×10^{5}	1.08×10^{6}	6.75×10^{5}	1.01×10^{6}	4.54×10^{5}	6.29×10^{5}	5.59×10^{5}	9.69×10^{5}	6.71×10^{5}	1.01×10^{6}	5.39×10^{5}	2.67×10^{5}	7.43×10^{5}	7.06×10^{5}	3.10×10^{5}	3.10×10^{5}
363	5-Ethyl-2,4-dimethylthiazole	6.96×10^{6}	2.17×10^{7}	3.74×10^{6}	6.63×10^{6}	3.94×10^{6}	4.01×10^{6}	2.59×10^{6}	5.69×10^{6}	3.75×10^{6}	3.85×10^{6}	1.93×10^{6}	1.20×10^{6}	3.47×10^{6}	3.19×10^{6}	8.62×10^{5}	8.57×10^{5}
364	2-Acetyl-4-methylthiazole	1.73×10^{7}	2.66×10^{7}	2.67×10^{7}	3.79×10^{7}	2.06×10^{7}	2.10×10^{7}	1.72×10^{7}	3.27×10^{7}	1.06×10^{7}	1.89×10^{7}	8.99×10^{6}	4.61×10^{6}	1.17×10^{7}	1.12×10^{7}	5.30×10^{6}	5.34×10^{6}
365	2-Propanoyl-thiazole	5.19×10^{6}	5.29×10^{6}	5.52×10^{6}	6.33×10^{6}	5.38×10^{6}	5.35×10^{6}	5.56×10^{6}	5.77×10^{6}	3.25×10^{6}	3.66×10^{6}	2.00×10^{6}	8.59×10^{5}	3.68×10^{6}	3.13×10^{6}	1.84×10^{6}	1.05×10^{6}
366	Benzothiazole	n.d.	n.d.	n.d.	n.d.	n.d.	n.d.	n.d.	n.d.	4.12×10^{6}	3.13×10^{6}	1.49×10^{6}	7.74×10^{5}	2.31×10^{6}	2.51×10^{6}	1.39×10^{6}	4.24×10^{5}

	**Tiophene compounds**	2.07×10^{8} (14.6)	2.48×10^{8} (2.5)	2.40×10^{8} (9.8)	2.33×10^{8} (3.4)	1.63×10^{8} (10.5)	2.18×10^{8} (9.4)	1.91×10^{8} (8.7)	1.86×10^{8} (9.8)	3.35×10^{8} (4.3)	3.44×10^{8} (7.5)	1.72×10^{8} (21.8)	1.13×10^{8} (26.7)	2.69×10^{8} (15.3)	2.55×10^{8} (15.3)	1.41×10^{8} (22.8)	8.21×10^{7} (4.1)
367	Thiophene	5.47×10^{6}	6.96×10^{6}	8.02×10^{6}	2.84×10^{6}	5.41×10^{5}	6.91×10^{6}	3.76×10^{6}	2.23×10^{6}	4.98×10^{7}	3.97×10^{7}	2.06×10^{7}	2.26×10^{7}	3.34×10^{7}	2.96×10^{7}	1.25×10^{7}	1.25×10^{7}
368	2-Methylthiophene	9.50×10^{6}	1.50×10^{7}	1.62×10^{7}	7.83×10^{6}	2.13×10^{6}	1.52×10^{7}	6.67×10^{6}	3.59×10^{6}	4.22×10^{7}	3.47×10^{7}	2.20×10^{7}	1.91×10^{7}	3.93×10^{7}	2.82×10^{7}	1.36×10^{7}	7.83×10^{6}
369	3-Methylthiophene	2.49×10^{6}	3.98×10^{6}	3.92×10^{6}	2.30×10^{6}	5.58×10^{5}	3.31×10^{6}	2.43×10^{6}	1.38×10^{6}	1.31×10^{7}	2.04×10^{7}	8.93×10^{6}	3.81×10^{6}	8.78×10^{6}	7.16×10^{6}	3.04×10^{6}	2.26×10^{6}
370	2-Ethylthiophene	1.31×10^{6}	1.85×10^{6}	1.88×10^{6}	1.44×10^{6}	3.91×10^{5}	1.56×10^{6}	1.10×10^{6}	7.60×10^{5}	1.77×10^{6}	1.57×10^{6}	1.11×10^{6}	8.97×10^{5}	2.50×10^{6}	2.31×10^{6}	7.86×10^{5}	6.00×10^{5}
371	2,5-Dimethylthiophene	1.88×10^{6}	1.90×10^{6}	1.87×10^{6}	1.99×10^{6}	1.58×10^{5}	2.25×10^{6}	1.58×10^{6}	1.71×10^{6}	1.16×10^{6}	1.76×10^{6}	1.09×10^{6}	9.24×10^{5}	2.06×10^{6}	1.49×10^{6}	8.38×10^{5}	4.81×10^{5}
372	2,4-Dimethylthiophene	1.93×10^{6}	3.52×10^{6}	3.13×10^{6}	2.70×10^{6}	8.15×10^{5}	2.88×10^{6}	2.08×10^{6}	1.54×10^{6}	3.01×10^{6}	5.25×10^{6}	2.33×10^{6}	1.92×10^{6}	4.09×10^{6}	4.56×10^{6}	1.54×10^{6}	1.10×10^{6}
373	2,3-Dimethylthiophene	1.67×10^{6}	1.56×10^{6}	1.64×10^{6}	1.24×10^{6}	4.34×10^{5}	1.54×10^{6}	1.02×10^{6}	7.80×10^{5}	2.67×10^{6}	3.14×10^{6}	1.81×10^{6}	1.65×10^{6}	3.26×10^{6}	2.48×10^{6}	1.14×10^{6}	6.85×10^{5}
374	2-Vinylthiophene	5.69×10^{6}	6.50×10^{6}	5.94×10^{6}	5.48×10^{6}	1.75×10^{6}	4.33×10^{6}	3.33×10^{6}	4.38×10^{6}	1.04×10^{7}	1.47×10^{7}	7.23×10^{6}	5.30×10^{6}	1.08×10^{7}	8.96×10^{6}	5.17×10^{6}	3.88×10^{6}
375	3-Methoxythiophene	1.50×10^{5}	1.27×10^{5}	1.14×10^{5}	1.03×10^{5}	3.93×10^{4}	7.15×10^{4}	6.66×10^{4}	1.15×10^{5}	6.18×10^{5}	7.09×10^{5}	2.62×10^{5}	1.30×10^{5}	3.66×10^{5}	2.99×10^{5}	1.19×10^{5}	1.20×10^{5}
376	3-Thiophanone	3.11×10^{6}	5.57×10^{6}	6.74×10^{6}	5.83×10^{6}	6.79×10^{5}	6.43×10^{6}	2.53×10^{6}	2.75×10^{6}	4.26×10^{6}	4.16×10^{6}	1.40×10^{6}	1.52×10^{6}	2.72×10^{6}	2.43×10^{6}	1.13×10^{6}	1.26×10^{6}
377	2-Isopropylthiophene	6.18×10^{6}	6.39×10^{6}	5.90×10^{6}	5.63×10^{6}	2.26×10^{6}	5.27×10^{6}	4.18×10^{6}	4.58×10^{6}	2.85×10^{6}	4.55×10^{6}	2.63×10^{6}	2.03×10^{6}	4.82×10^{6}	4.29×10^{6}	2.08×10^{6}	1.38×10^{6}
378	2,3,4-Trimethylthiophene	8.49×10^{5}	9.85×10^{5}	1.00×10^{6}	8.55×10^{5}	3.69×10^{5}	9.18×10^{5}	6.64×10^{5}	5.53×10^{5}	5.86×10^{5}	7.86×10^{5}	4.61×10^{5}	2.07×10^{5}	6.66×10^{5}	5.44×10^{5}	2.50×10^{5}	9.73×10^{4}
379	Dihydro-2-methyl-3(2H)-thiophenone	1.71×10^{7}	2.78×10^{7}	2.79×10^{7}	2.72×10^{7}	6.77×10^{6}	2.66×10^{7}	1.89×10^{7}	1.63×10^{7}	3.08×10^{7}	3.53×10^{7}	1.50×10^{7}	1.09×10^{7}	2.71×10^{7}	3.06×10^{7}	2.02×10^{7}	9.16×10^{6}
380	3-Thiophenecarboxaldehyde	1.78×10^{6}	2.21×10^{6}	2.63×10^{6}	2.53×10^{6}	2.16×10^{6}	1.89×10^{6}	2.00×10^{6}	2.10×10^{6}	1.86×10^{6}	2.28×10^{6}	9.64×10^{5}	4.80×10^{5}	1.91×10^{6}	1.55×10^{6}	9.97×10^{5}	2.89×10^{5}
381	Dihydro-2(3H)-thiophenone	9.24×10^{6}	7.76×10^{6}	7.53×10^{6}	6.97×10^{6}	5.20×10^{6}	8.31×10^{6}	7.12×10^{6}	6.59×10^{6}	5.48×10^{6}	5.49×10^{6}	2.81×10^{6}	1.79×10^{6}	3.54×10^{6}	4.09×10^{6}	2.10×10^{6}	1.81×10^{6}
382	2-Thiophenecarboxaldehyde	5.00×10^{7}	5.29×10^{7}	5.85×10^{7}	5.80×10^{7}	5.83×10^{7}	5.20×10^{7}	5.71×10^{7}	4.40×10^{7}	7.15×10^{7}	6.42×10^{7}	3.06×10^{7}	1.53×10^{7}	5.09×10^{7}	5.25×10^{7}	3.12×10^{7}	1.57×10^{7}
383	3-Methyl-2-thiophenecarboxaldehyde	1.10×10^{7}	1.20×10^{7}	1.27×10^{7}	1.38×10^{7}	1.02×10^{7}	1.05×10^{7}	1.03×10^{7}	9.29×10^{6}	1.54×10^{7}	1.99×10^{7}	1.04×10^{7}	4.64×10^{6}	1.49×10^{7}	1.40×10^{7}	8.13×10^{6}	4.69×10^{6}
384	3-Acetylthiophene	2.81×10^{7}	1.67×10^{7}	2.09×10^{7}	3.02×10^{7}	1.83×10^{7}	1.92×10^{7}	2.18×10^{7}	2.24×10^{7}	1.42×10^{7}	1.69×10^{7}	8.37×10^{6}	3.64×10^{6}	1.34×10^{7}	1.24×10^{7}	8.55×10^{6}	3.64×10^{6}
385	2-Acetylthiophene	2.89×10^{7}	4.26×10^{7}	3.32×10^{7}	3.21×10^{7}	3.29×10^{7}	2.87×10^{7}	2.51×10^{7}	3.62×10^{7}	3.31×10^{7}	3.31×10^{7}	2.08×10^{7}	8.58×10^{6}	2.72×10^{7}	2.56×10^{7}	1.31×10^{7}	9.32×10^{6}
386	2,5-Diethylthiophene	2.71×10^{6}	3.20×10^{6}	2.82×10^{6}	2.80×10^{6}	1.96×10^{6}	2.99×10^{6}	2.54×10^{6}	2.74×10^{6}	1.25×10^{6}	1.55×10^{6}	1.16×10^{6}	5.96×10^{5}	1.50×10^{6}	1.35×10^{6}	8.18×10^{5}	5.44×10^{5}
387	Methyl-2-thiophene carboxylate	2.30×10^{6}	2.33×10^{6}	2.18×10^{6}	2.48×10^{6}	1.75×10^{6}	2.07×10^{6}	1.89×10^{6}	2.53×10^{6}	3.07×10^{6}	4.27×10^{6}	2.27×10^{6}	9.24×10^{5}	2.78×10^{6}	3.73×10^{6}	1.48×10^{6}	9.66×10^{5}
388	5-Methyl-2-thiophenecarboxaldehyde	7.81×10^{6}	7.44×10^{6}	7.96×10^{6}	8.52×10^{6}	7.30×10^{6}	6.91×10^{6}	6.91×10^{6}	7.45×10^{6}	1.03×10^{7}	1.03×10^{7}	5.56×10^{6}	2.05×10^{6}	7.07×10^{6}	6.39×10^{6}	3.46×10^{6}	2.23×10^{6}
389	2-Pentylthiophene	1.40×10^{6}	9.20×10^{5}	1.09×10^{6}	1.49×10^{6}	1.20×10^{6}	1.25×10^{6}	7.20×10^{5}	1.06×10^{6}	3.51×10^{5}	3.48×10^{5}	3.36×10^{5}	2.11×10^{5}	3.76×10^{5}	2.40×10^{5}	9.38×10^{4}	1.30×10^{5}
390	2-Propionylthiophene	6.30×10^{6}	1.78×10^{7}	5.80×10^{6}	8.31×10^{6}	6.22×10^{6}	7.18×10^{6}	7.17×10^{6}	1.08×10^{7}	1.51×10^{7}	1.85×10^{7}	3.55×10^{6}	4.28×10^{6}	5.07×10^{6}	1.01×10^{7}	8.79×10^{6}	1.39×10^{6}

The designations Dec, 1-6, and Sup refer to each blend type under study: Blend Dec – decaffeinated coffee blend, blends with labelled intensities (Blend 1 – intensity 5, Blend 2 – intensity 9, Blend 3 – intensity 10), selected origin blends (Blend 4 – Jamaica, Blend 5 – Tanzania, Blend 6 – Ethiopia), and Blend Sup – blend supplemented with natural extracts of ginseng and guarana. The GC × GC peak areas were given in arbitrary units. For the chemical families the RSD (relative standard deviation, %) is also shown. n.d. - not detected in the conditions used.
